# Spread of the Emerging Viral Hemorrhagic Septicemia Virus Strain, Genotype IVb, in Michigan, USA

**DOI:** 10.3390/v4050734

**Published:** 2012-05-03

**Authors:** Mohamed Faisal, Megan Shavalier, Robert K. Kim, Elena V. Millard, Michelle R. Gunn, Andrew D. Winters, Carolyn A. Schulz, Alaa Eissa, Michael V. Thomas, Martha Wolgamood, Gary E. Whelan, James Winton

**Affiliations:** 1 Department of Pathobiology and Diagnostic Investigation, College of Veterinary Medicine, Michigan State University, East Lansing, MI 48824, USA; Email: shavali1@cvm.msu.edu (M.S.); kimrober@cvm.msu.edu (R.K.K.); millarde@cvm.msu.edu (E.V.M.); gunnmich@cvm.msu.edu (M.R.G.); 2 Department of Fisheries and Wildlife, College of Agriculture and Natural Resources, Michigan State University, East Lansing, MI 48824, USA; Email: winter28@msu.edu (A.D.W.); schulzc2@msu.edu (C.A.S.); 3 Department of Fish Diseases and Management, Cairo University, Cairo, Egypt; Email: aeissa2005@gmail.com; 4 Michigan Department of Natural Resources, State of Michigan Government, Lansing, MI 48909, USA; Email: thomasm4@michigan.gov (M.V.T.); wolgamoodm@michigan.gov (M.W.); whelang@michigan.gov (G.E.W.); 5 United States Geological Survey-Western Fisheries Research Center, Seattle, WA 98115, USA; Email: jwinton@usgs.gov

**Keywords:** Michigan, viral hemorrhagic septicemia virus, Laurentian Great Lakes, emerging disease

## Abstract

In 2003, viral hemorrhagic septicemia virus (VHSV) emerged in the Laurentian Great Lakes causing serious losses in a number of ecologically and recreationally important fish species. Within six years, despite concerted managerial preventive measures, the virus spread into the five Great Lakes and to a number of inland waterbodies. In response to this emerging threat, cooperative efforts between the Michigan Department of Natural Resources (MI DNR), the Michigan State University Aquatic Animal Health Laboratory (MSU-AAHL), and the United States Department of Agriculture-Animal and Plant Health Inspection Services (USDA-APHIS) were focused on performing a series of general and VHSV-targeted surveillances to determine the extent of virus trafficking in the State of Michigan. Herein we describe six years (2005–2010) of testing, covering hundreds of sites throughout Michigan’s Upper and Lower Peninsulas. A total of 96,228 fish representing 73 species were checked for lesions suggestive of VHSV and their internal organs tested for the presence of VHSV using susceptible cell lines. Of the 1,823 cases tested, 30 cases from 19 fish species tested positive for VHSV by tissue culture and were confirmed by reverse transcriptase polymerase chain reaction (RT-PCR). Gene sequence analyses of all VHSV isolates retrieved in Michigan demonstrated that they belong to the emerging sublineage “b” of the North American VHSV genotype IV. These findings underscore the complexity of VHSV ecology in the Great Lakes basin and the critical need for rigorous legislation and regulatory guidelines in order to reduce the virus spread within and outside of the Laurentian Great Lakes watershed.

## 1. Introduction

One of the most serious pathogens of finfish is the viral hemorrhagic septicemia virus (VHSV), a member of the genus *Novirhabdovirus*, family Rhabdoviridae, order Mononegavirales. The virus is recognized for its devastating effects on over 80 marine and freshwater fish species in the Northern Hemisphere and is a World Organization for Animal Health (OIE) reportable fish pathogen [1].

For five decades after the first description of the disease in Germany in the 1930s [2], VHSV has been a major cause of severe losses in European rainbow trout (*Oncorhynchus mykiss*) farms. In the 1980s, VHSV emerged in the Pacific Northwest region of North America, causing fatal disease in fish from marine and brackish environments of Washington [3,4], Alaska [5,6], and California [7]. Further epizootiological investigations in Europe demonstrated the widespread presence of VHSV in the marine environments of the Baltic Sea [8], Scotland [9], the English Channel [10], Kattegat, Skagerrak and North Sea [11], Japan [12], and Korea [13].

The disease can take acute, subacute, or chronic courses [14]. VHSV is believed to target the endothelial lining of blood vessels [15,16], causing petechial and/or ecchymotic hemorrhaging of cutaneous and subcutaneous skin layers, muscle, and internal organs, and subsequent anemia and severe gill pallor. Clinical signs and outcome of the infection vary depending on VHSV genotype, fish species, age, stress level, temperature and other environmental factors [17]. VHSV-infected fish suffer severe tissue alterations such as necrotic degeneration of the kidneys, spleen, liver, and intestine [18,19,20,21,22,23,24,25].

The VHSV virion has a bullet-shaped capsid and is encased in an envelope. The virus has a single, negative-stranded ribonucleic acid (RNA) genome comprised of 11,184 nucleotides, and contains six open reading frames in the order 3'-N-P-M-G-NV-L-5' [26,27]. These genes are separated by conserved gene junctions with di-nucleotide gene spacers. The N (nucleocapsid) gene encodes 38–41 kDa proteins that are arranged tightly around the viral RNA genome forming the N-RNA complex which serves as the template for both transcription and replication [28]. The viral RNA-dependent RNA polymerase is a two subunit complex that consists of a large (157–190 kDa) subunit L [29], and a non-catalytic cofactor, the phosphoprotein P [30]. P also acts as a chaperone of nascent RNA-free N by forming an N(0)-P complex that prevents N from binding to cellular RNAs and from polymerizing in the absence of RNA [30]. The M gene encodes the 19 kDa matrix protein believed to act as a bridge between the viral envelope and nucleocapsid in rhabdoviruses [28] and plays a regulatory role in viral transcription, replication, production, and budding in rhabdoviruses [31]. The G gene encodes the 72–80 kDa major surface glycoprotein antigen located on the envelope surface and is believed to be important for virus attachment to susceptible cells [28,32]. The NV gene encodes a nonstructural protein that may be involved in the virulence [33]. 

Phylogenetic typing, based on sequences of the G and N genes, is the most commonly used method for VHSV strain identification. Studies of hundreds of isolates retrieved from infected fish worldwide have identified four distinct VHSV genotypes, designated I-IV [34,35]. Each of the genotypes has a distinct geographic and host range. Genotype I strains are classified into five sublineages (a–e) that mainly infect farmed rainbow trout in Europe and a few marine fish species from Germany, the English Channel, Baltic Sea, Skagerrak and Kattegat. Genotype II strains exist in the Baltic Sea. Genotype III includes a number of marine isolates collected in coastal waters of the North Sea around Ireland and the United Kingdom. Genotype IV isolates occur primarily in North America, but also in Japan and Korea [13,36,37].

In 2003, a virus was isolated from samples of kidney and spleen tissues of adult muskellunge (*Esox masquinongy*) collected from the Grosse Pointe Yacht Club site, Lake St. Clair, Michigan by the Michigan State University Aquatic Animal Health Laboratory (MSU-AAHL) [38]. Electron microscopy revealed that the virus exhibits the characteristic bullet shape morphology of rhabdoviruses. Further characterization of this isolate, including phylogenetic analysis of the glycoprotein gene, demonstrated that the Great Lakes virus is most closely related to the North American and Japanese genotype (IVa) of VHSV. Because of distinct differences in gene sequence, this isolate was placed into a new sublineage within genotype IV, designated VHSV-IVb [38]. The expansive and highly adaptable nature of this VHSV isolate to the freshwater environment of the Great Lakes was soon realized when reports came detailing the isolations of the virus from mass mortality events in different parts of the Laurentian Great Lakes basin [39,40] (reviewed in [23,24]).

The three objectives of this manuscript are to describe the efforts made in the State of Michigan to determine the cause of fish kills and to monitor the spread of the emerging VHSV strain in wild and propagated fish populations in Michigan since its first appearance in Lake St. Clair; to provide an overview of the managerial measures adopted by state and federal agencies in response to VHSV emergence; and to summarize the new research findings gained on the biology of VHSV-IVb since its emergence in the Laurentian Great Lakes. This study represents the most extensive disease targeted surveillance ever conducted in the Great Lakes basin.

## 2. Results and Discussion

### 2.1. VHSV Spread in Michigan 2003–2010

#### 2.1.1. 2003–2005

VHSV-IVb has been present in the Laurentian Great Lakes basin since at least 2003. Prior annual testing of propagated fish and wild fish surveys conducted in Michigan since 1970 never detected VHSV or other rhabdoviruses. The virus was isolated by the MSU-AAHL from muskellunge collected from the western part of Lake St. Clair, Michigan in 2003 [38]. Attempts to isolate VHSV again from Lake St. Clair fish in 2004 were unsuccessful.

It appears VHSV may have been present in eastern Canada as early as 2000. Gagné *et al.* [41] reported its isolation from both mummichog (*Fundulus heteroclitus*) and three-spined stickleback (*Gasterosteus aculeatus aculeatus*) collected from Ruisseau George Collette near Bouctouche, New Brunswick (NB), Canada following epizootic events in the river. Subsequent isolations of this same VHSV strain also occurred in 2002 and 2004 from mortality events that involved striped bass (*Morone saxatilis*) in the Miramichi Bay, Baie du Vin, NB [41]. The same authors isolated VHSV from a single sea run brown trout (*Salmo trutta trutta*) that was found dead in the French River, Nova Scotia (NS), Canada [41]. Gagné *et al.* [41] suggested that VHSV may have existed off the Atlantic coast of North America prior to its emergence in the Great Lakes basin. More recent phylogenetic analysis, however, suggested that the Atlantic Canadian VHSV strain may be slightly distinct from both VHSV genotypes IVa and IVb [42].

In April 2005, a large fish kill was reported from the Bay of Quinte, Lake Ontario, Canada that primarily involved tens of thousands of freshwater drum(*Aplodinotus grunniens*) [39]*.* The authors also noted that large numbers of round goby (*Neogobius melanostomus*) and a few muskellunge were involved in the mortality episode. VHSV-IVb was isolated from the dead fish and visualized within the tissues of affected drum by electron microscopy. This was the first report of VHSV-IVb associated with a major mortality event in the Laurentian Great Lakes.

**Figure 1:**
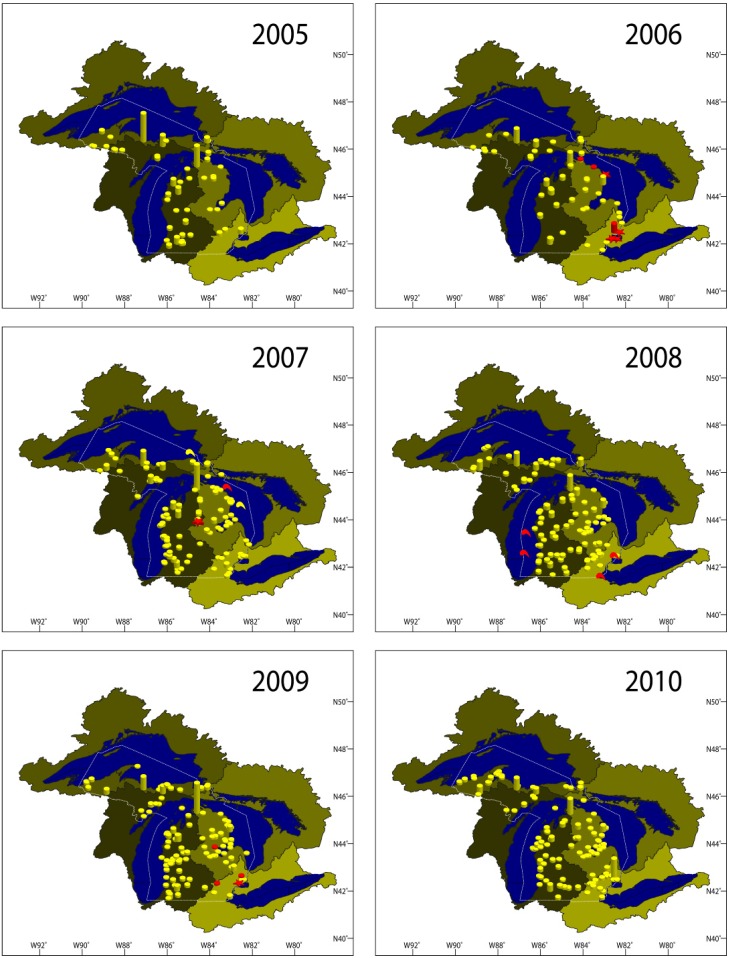
A map of the State of Michigan showing sites from which samples were collected for the viral hemorrhagic septicemia virus testing divided by year (2005–2010). Yellow symbols indicate negative sites, red symbols indicate positive sites, red stars indicate positive sites with fish kills, and comma-like shapes indicate sites where macroinvertebrates were sampled. The different color shades outline the watershed of each of the Great Lakes.

In 2005, the MSU-AAHL tested 11,334 fish for VHSV representing 30 species and grouped into 231 cases. For purpose of this manuscript, a case is defined as a group of fish of the same species, source, and date of submission. For hatchery-raised fish, all fish in one case are also of the same age and strain. Scientific names of all fish species tested in this study are given in Supplementary Table S1. Geographic locations, fish species, and numbers are given in Figure 1 and Supplementary Table S2. Wild fish cases tested for VHSV included 62 pre-transfer inspections, coolwater broodstock inspections, and general pathogen surveys from 25 sites throughout Michigan (21 species) and six diagnostic cases associated with mortality episodes. Three Michigan aquaculture facilities submitted three species that were tested for VHSV. Samples were also collected from 733 feral fish captured at weirs as they were returning to spawn in six Michigan rivers. Species included steelhead (*Oncorhynchus mykiss*), coho salmon (*O. kisutch*), chinook salmon (*O. tshawytscha*), and Atlantic salmon (*Salmo salar*). Six Michigan state fish hatcheries and the Lake Superior State Aquatic Research Laboratory (LSSU-ARL) submitted 135 cases of broodstock and production fish for health inspections and diagnostic investigations that were tested for VHSV. Lake whitefish (*Coregonus clupeaformis*) collected from four sites in northern Lakes Michigan and Huron (15 cases, 419 fish) were also tested for VHSV. No VHSV was detected in Michigan during this year.

#### 2.1.2. 2006

**Figure 2:**
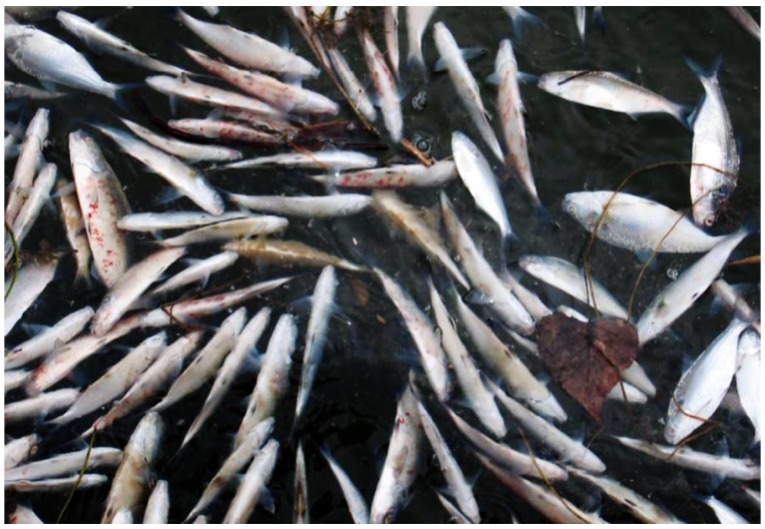
Widespread gizzard shad mortality in Lake St. Clair, Michigan that took place in March 2006. The viral hemorrhagic septicemia virus (genotype IVb) was isolated from affected fish.

**Figure 3:**
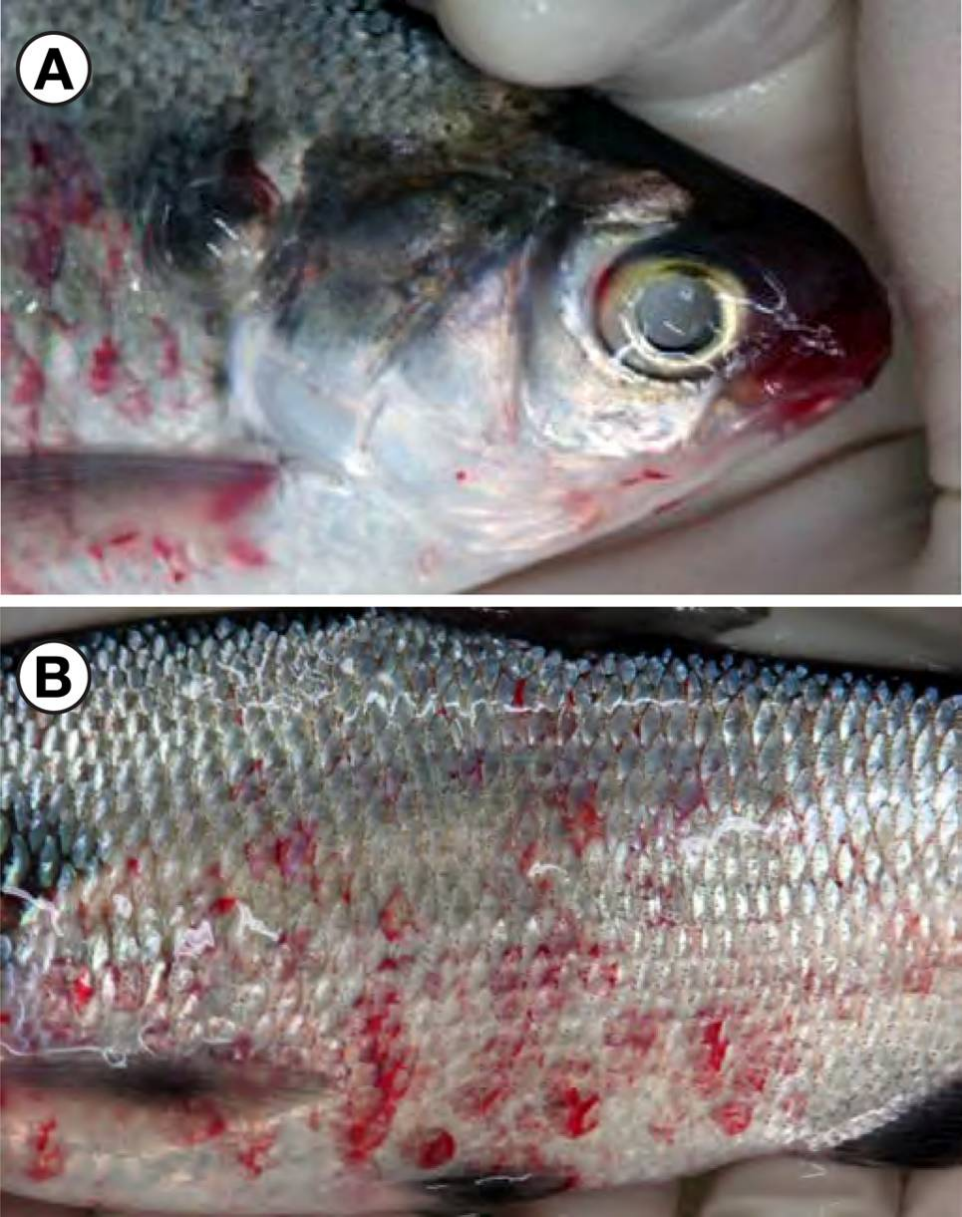
Gizzard shad infected with the viral hemorrhagic septicemia virus. Notice the petechial hemorrhages around the mouth (**A**) and sides of the body (**B**).

During 2006, multiple fish kills occurred in the Laurentian Great Lakes basin that were associated with VHSV-IVb (Table 1). During early March 2006, thousands of dead and dying gizzard shad (*Dorosoma cepedianum*) were found along the St. Clair River and associated canals in the Marine City and Algonac areas, Lake St. Clair, and the Detroit River, MI (Figure 2). The fish exhibited pale gills and petechial and ecchymotic hemorrhages on both sides of the body, around the mouth, eyes, and vent (Figure 3). Laboratory analysis revealed the presence of VHSV-IVb in the tissues and sera of gizzard shad.

**Table 1:** Cases from which the viral hemorrhagic septicemia virus, genotype IVb, was isolated and confirmed in Michigan (2005–2010).

Date of sampling	Location	Wild species collected	Purpose of investigation	Latitude	Longitude
3/14/06	Lake St. Clair	gizzard shad	mortalities	42.631389	−82.5375
4/26/06	Lake St. Clair	muskellunge	mortalities	42.343	−82.902
		yellow perch	mortalities	42.343	−82.902
5/17/06	Lake St. Clair	northern pike	mortalities	42.632	−82.776
		muskellunge	mortalities	42.632	−82.776
		shorthead redhorse	mortalities	42.632	−82.776
		freshwater drum	mortalities	42.632	−82.776
		rockbass	mortalities	42.632	−82.776
		silver redhorse	mortalities	42.632	−82.776
5/22/06	Lake St. Clair	freshwater drum	mortalities	42.632	−82.776
		rockbass	mortalities	42.632	−82.776
		northern pike	mortalities	42.632	−82.776
		muskellunge	mortalities	42.632	−82.776
5/24/06	Lake St. Clair	freshwater drum	mortalities	42.632	−82.776
		muskellunge	mortalities	42.632	−82.776
7/10/06	Lake St. Clair	muskellunge	mortalities	42.333333	−82.666667
8/18/06	Lake Huron, Cheboygan	lake whitefish	research	45.694798^*^	−84.355815
9/28/06	Lake Huron, Swan River Weir	feral spawning chinook salmon	inspection	45.402803	−83.734867
10/6/06	Lake Huron, Thunder Bay	lake whitefish	mortalities	45.05	−83.2
		walleye	mortalities	45.05	−83.2
12/13/06	Lake St. Clair	spottail shiner	surveillance	42.632^*^	−82.776
		emerald shiner	surveillance	42.632^*^	−82.776
5/2/07	Budd Lake, Clare County	black crappie	mortalities	44.01585	−84.788083
		bluegill	mortalities	44.01585	−84.788083
		pumpkinseed	mortalities	44.01585	−84.788083
		largemouth bass	mortalities	44.01585	−84.788083
5/11/09	Baseline Lake, Livingston County	brown bullhead	surveillance	42.42724	−83.89926
5/18/09	Lake St. Clair	spawning muskellunge	inspection	42.6157^*^	−82.757
6/4/09	Lake St. Clair	smallmouth bass	mortalities	42.475507	−82.879249
12/16/09	Bait Facility, Bay County	spottail shiner	inspection	43.932527	−84.001776

* coordinates approximated.

Later in the spring of 2006, another episode of mortalities was observed in Lake St. Clair and the Detroit River. The primary species involved were yellow perch (*Perca flavescens*) and muskellunge. VHSV-IVb was isolated from muskellunge collected from Lake St. Clair near the head of the Detroit River and from yellow perch collected from Lake St. Clair near Huron Point. A minimum of 2,000 mortalities were estimated in muskellunge ranging in size from 760–1260 mm total length. Estimating the total number of dead yellow perch was not feasible because they were small in size (100–250 mm) and widely scattered throughout the lake. Within the same time frame, hundreds of dead and dying fish in shoreline canals located in Harrison Township along Lake St. Clair were reported. Affected fish species included pumpkinseed (*Lepomis gibbosus*), bluegill (*L. macrochirus*), black crappie (*Pomoxis nigromaculatus*), largemouth bass (*Micropterus salmoides*), yellow perch, and common carp (*Cyprinus carpio*).

**Figure 4:**
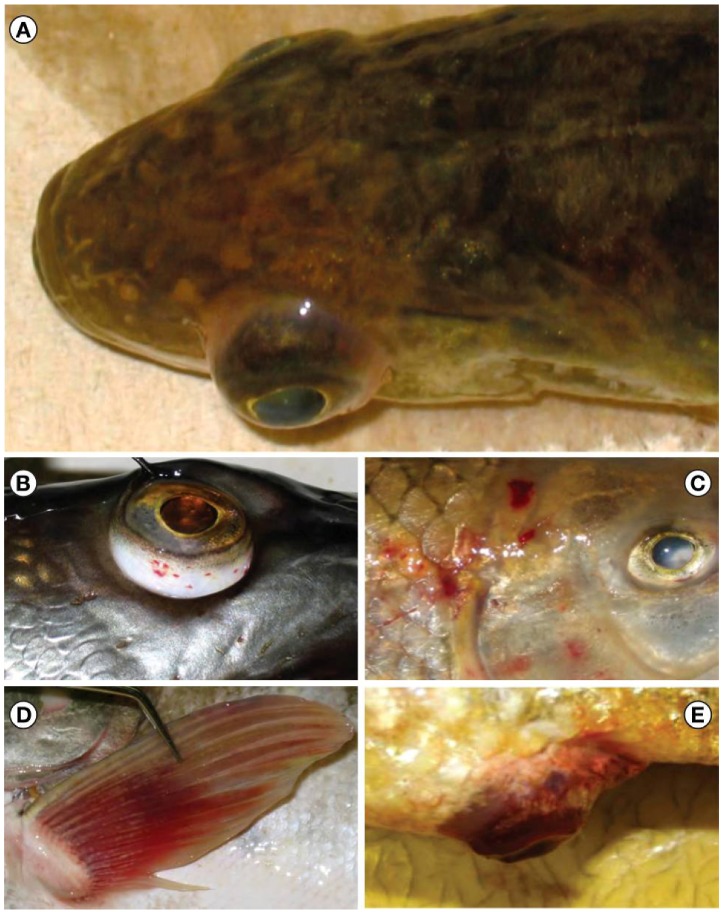
External gross lesions observed on fish from Lake St. Clair during the viral hemorrhagic septicemia virus die off in 2006. Among the signs were exophthalmia (**A**, yellow perch), petechial hemorrhages around the eyes (**B**, muskellunge), ecchymotic hemorrhages on the sides of the body (**C**, freshwater drum) and at the base of fins (**D**, muskellunge), and hemorrhagic protruded vent (**E**, freshwater drum).

**Figure 5:**
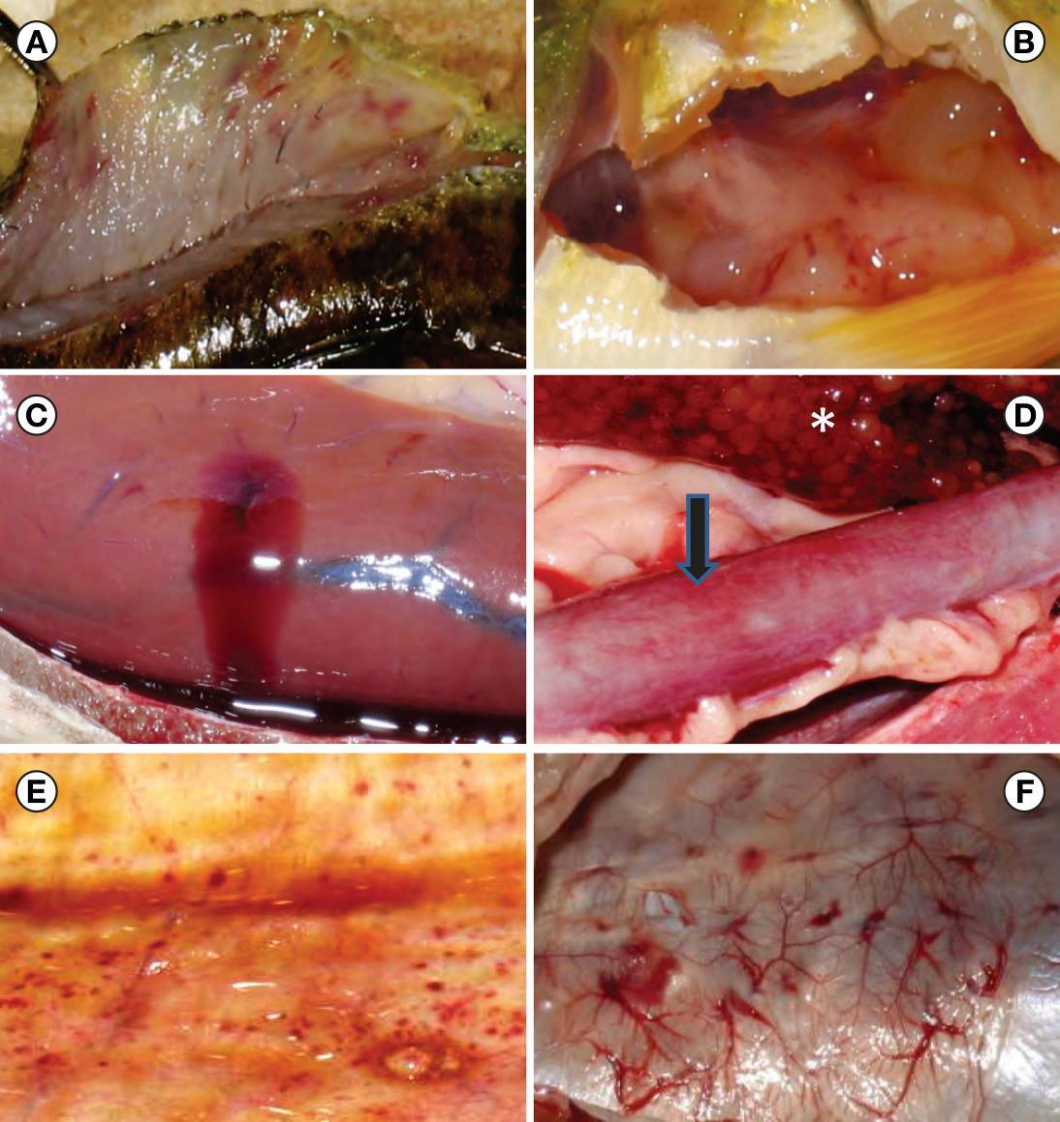
Internal gross lesions observed in Lake St. Clair fish during the viral hemorrhagic septicemia virus die off in 2006. Among the signs were hemorrhages in the musculature (**A**, yellow perch), petechial hemorrhages in the visceral adipose tissue (**B**, yellow perch), liver bleeding (**C**, northern pike), hemorrhagic enteritis (arrow) and hemorrhages within the ovary (asterix) of a ripe muskellunge (**D**), petechial hemorrhages and membrane thickening of the swimbladder (**E**, muskellunge), and several areas of congestion in the vasculature of the swimbladder (**F**, freshwater drum).

These fish kill episodes coincided with a sharp warming of Lake St. Clair water in late March and April of 2006. By mid-May, reports of dead fish had ceased. Therefore, MI DNR initiated a trap net survey in Anchor Bay, Lake St. Clair and fish were collected at three occasions between May 17 and 24, 2006. Apparently healthy and clinically ill representatives of six species were analyzed for VHSV. The virus was found in muskellunge, freshwater drum, northern pike (*Esox lucius*), rock bass (*Ambloplites rupestris*), silver redhorse (*Moxostoma anisurum*), and shorthead redhorse (*M. macrolepidotum*). Infected fish exhibited external hemorrhages at different sites of the body and at the base of fins, exophthalmia, and protruded vents (Figure 4). Internally, fish exhibited congestion and hemorrhages in organs such as the liver, swimbladder and intestine (Figure 5). In July of that year, the virus was found again in a Lake St. Clair muskellunge caught during a monthly fishing tournament. This fish suffered extensive hemorrhaging within the mouth, musculature, swimbladder, stomach, intestine, and ovaries.

During autumn of 2006, VHSV was isolated from fish at multiple sites in Lake Huron, further extending the geographic range of the virus in Michigan waters. VHSV was isolated from a lake whitefish collected in August from a site near Cheboygan in northern Lake Huron, which exhibited severe dermal hemorrhages and splenic congestion. In September, the virus was isolated from the ovarian fluid of an apparently healthy chinook salmon returning to spawn at the Swan River Weir (Lake Huron watershed). VHSV-IVb was also found to be associated with two small fish kills in Thunder Bay in October involving lake whitefish and walleye (*Sander vitreus*) that displayed hemorrhagic lesions consistent with VHSV. 

Alarmed by these reports, the MI DNR, MSU-AAHL, and USDA-APHIS collaboratively initiated a statewide, multiyear survey in December of 2006 to follow-up on these fish kill investigations and to determine the spread of VHSV-IVb. Spottail shiners (*Notropis hudsonius*) and emerald shiners (*N. atherinoides*) were collected from eight sites in Lakes Huron, Erie, and St. Clair. Moreover, walleye and emerald shiners were collected from three Michigan rivers. From these targeted surveillance samples, the virus was recovered only from spottail shiners and emerald shiners collected from Lake St. Clair.

Other VHSV testing involved 38 cases of wild fish for coolwater broodstock screening, health inspections, general pathogen surveys, and mortality episodes; 49 cases from 11 Michigan aquaculture facilities; and 90 cases from Michigan state fish hatcheries, LSSU-ARL, and salmonid spawning runs. This is in addition to 12 cases of lake whitefish in northern Lakes Michigan and Huron (353 fish) tested for VHSV. Findings of all VHSV-tested cases in 2006 are displayed in Figure 1 and Supplementary Table S3.

Outside of Michigan, a number of VHSV-IVb associated fish kills were observed in New York State. In May 2006, Groocock *et al.* [40] reported widespread mortality among round goby in New York state waters of Lake Ontario and the St. Lawrence River that was associated with the presence of VHSV-IVb in the tissues. In August 2006, VHSV-IVb was found associated with a mortality event that involved walleye in Conesus Lake, an inland lake of New York in the Lake Ontario drainage. This was the first record that VHSV had spread into inland lakes (reviewed in [24]). Later in 2006 (August–December), Frattini *et al.* [43] surveyed 1,011 apparently healthy fish representing 20 species from 19 different bodies of water throughout New York State for the presence of VHSV. The virus was isolated from bluntnose minnows (*Pimephales notatus*) from the St. Lawrence River and emerald shiners from Lake Erie and the Niagara River. Site prevalence by conventional methods in positive sites ranged from 25% to 100%. VHSV was also detected in tissue samples from eight species using a quantitative reverse transcriptase polymerase chain reaction (qRT-PCR) assay. These species included bluntnose minnow, coho salmon, emerald shiner, pumpkinseed, smallmouth bass (*Micropterus dolomieui*), walleye, white perch (*Morone americana*) and yellow perch. These positive detections were collected from Chautauqua Lake, Lake Erie, Niagara River, Oneida Lake, Otisco Lake, Red Lake, Salmon River, Seneca Lake, St. Lawrence River, Upper Hudson River and Whitney Point Reservoir. By the end of 2006, the geographic range of VHSV-IVb extended from the Lower Great Lakes Basin to northern Lake Huron.

#### 2.1.3. 2007

A total of 14,201 fish (307 cases, 42 species) were tested for the presence of VHSV (Figure 1 and Supplementary Table S4). VHSV-IVb was isolated from fish in four cases (Table 1), all of which were sampled in May. Budd Lake (Lake Huron Basin, Clare County, MI, USA) experienced a mortality event that involved several thousand black crappie, bluegill, pumpkinseed, and largemouth bass along with a few muskellunge exhibiting hemorrhagic signs consistent with VHSV. VHSV-IVb was isolated and confirmed from these species. This alarmed managers, as this was the first indication that VHSV had spread to an inland lake in Michigan. Budd Lake is isolated from both Lakes Huron and Michigan. Interestingly, 309 fish collected from the same sites in Budd Lake 35 days following the mortality episode, including bluegill, pumpkinseed, and largemouth bass, tested negative for VHSV. 

Samples tested for VHSV in 2007 included 18 cases from aquaculture and bait collection facilities, and 109 cases from LSSU-ARL, Michigan state fish hatcheries, and gamete collecting weirs. MI DNR also surveyed wild fish from 59 sites throughout Michigan’s Upper and Lower Peninsulas, including 27 sites in Lakes Superior, Michigan, Huron, Erie, and St. Clair; 24 inland lakes and ponds; and eight rivers. Other wild fish samples came from 22 cases that involved coolwater broodstock screening, health inspections, and mortality episodes. Besides the Budd Lake isolation, VHSV was not detected in any other fish tested in Michigan that year.

By the early spring of 2007, VHSV-IVb was detected again in New York and Ohio. Kane-Sutton *et al.* [44] reported the isolation of the virus from spawning yellow perch in central Lake Erie basin. VHSV then emerged west of Lake Michigan in the State of Wisconsin. The Wisconsin Department of Natural Resources (WI DNR) reported large numbers of dead freshwater drum in Lake Winnebago and Little Lake Butte des Morts from which VHSV-IVb was isolated (WI DNR News Release May 18, 2007). Concerns regarding spread of the virus into Lake Michigan were soon justified when VHSV was isolated from smallmouth bass, lake whitefish, and brown trout from Wisconsin waters of Green Bay and Lake Michigan (Wisconsin DNR News Release May 24, 2007). VHSV-IVb was also isolated from *Diporeia* spp. collected from Michigan waters of Lake Huron in August of this year, demonstrating for the first time that invertebrates are also capable of harboring the virus [45].

#### 2.1.4. 2008

A total of 15,895 fish (350 cases of 48 different species) were tested for VHSV (Figure 1, Supplementary Table S5). VHSV-targeted surveillance accounted for 167 cases (7,672 fish, 33 species) from 14 Great Lakes sites within Lakes Superior, Michigan, Huron, Erie, and St. Clair; 37 inland lakes and ponds; 10 rivers; and one creek. Other wild populations were tested from 15 sites for routine health inspections and from two sites following mortality episodes. Private facilities accounted for 58 cases: 47 from six Michigan aquaculture facilities and 11 cases of emerald and spottail shiners from four bait collection facilities. Michigan state fish hatcheries and the LSSU-ARL submitted 74 cases of broodstock and production lots for fish health inspections and diagnostics. Feral spawning steelhead, chinook salmon, coho salmon, and Atlantic salmon were inspected at four Michigan weirs. None of the fish collected in 2008 tested positive for VHSV. However, VHSV-IVb was again isolated from *Diporeia* spp. collected from Lakes Michigan and Ontario in April of 2008 [45] and from piscicolid leeches (*Myzobdella lugubris*) collected from Lake St. Clair in May and from western Lake Erie, MI in June [46]. These reports support the potential role that macro-invertebrates could have in VHSV spread and persistence in the ecosystem.

During this same year, VHSV was isolated from Skaneateles Lake, New York. Affected species involved smallmouth bass, rock bass and lake trout (*Salvelinus namaycush*) (New York Department of Environmental Conservation Press Release June 19, 2008). VHSV was also isolated from rainbow trout caught from the Little Salmon River, bluegill from the Seneca-Cayuga Canal, and bluegill collected from a privately owned pond in Ransomville, New York. Another notable isolation of VHSV in 2008 was from sea lamprey (*Petromyzon marinus*) collected at multiple sites within the Cheboygan River, Greek Creek, and Ocqueoc River (La Crosse Fish Health Center, Fish Health Newsletter Winter 2008).

VHSV-IVb continued its spread in Lake Michigan in the early summer months of 2008. A mass mortality of round goby took place in Lake Michigan near Milwaukee (WI DNR Press Release June 5, 2008). The virus was also isolated from yellow perch collected during a survey in Lake Michigan (WI DNR Press Release June 13, 2008). Soon thereafter, the Illinois DNR announced in a press release dated July 2, 2008 that VHSV-IVb was isolated from rock bass and round goby from Lake Michigan. Surprisingly, the U.S. Fish and Wildlife Service La Crosse Fish Health Center announced in a press release dated June 17, 2008 that the virus was isolated and confirmed in ovarian fluid of muskellunge collected in April from Clear Fork Reservoir, an inland lake located in north central Ohio. This isolation marked the first occurrence of VHSV outside the Great Lakes basin, a matter that raised concerns for the possible virus spread to other waterbodies in North America.

#### 2.1.5. 2009

A total of 21,775 fish (51 species) were tested for VHSV in Michigan (Figure I, Supplementary Table S6). In 2009, the Michigan Department of Agriculture (MDA) and the United States Department of Agriculture-Animal and Plant Health Inspection Services (USDA-APHIS) worked with the MSU‑AAHL on a VHSV initiative for Michigan’s private aquaculture industry. Altogether, 17 of the 18 aquaculture facilities that submitted samples to the MSU-AAHL for VHSV testing in 2009 participated in this initiative. Aquaculture and bait collection facilities accounted for 83 cases submitted to MSU-AAHL during this year. LSSU-ARL and Michigan state fish hatcheries submitted 109 cases for fish health inspections and diagnostics, including 50 cases of broodstock and 59 cases of production fish. Five cases of salmonid returning spawners were tested from four Michigan weirs. Wild fish sampled for MI DNR VHSV-targeted surveillance included 143 cases (7,092 fish, 36 species) from 20 Great Lakes sites throughout Lakes Superior, Michigan, Huron, Erie, and St. Clair; 27 inland lakes and ponds; seven rivers; and five creeks. Additionally, VHSV testing in wild populations included 15 cases of mortality episodes at six sites and 18 coolwater broodstock inspection cases from seven sites.

Of these sampling events, VHSV-IVb was isolated from four cases in Michigan (Table 1). The virus was isolated in May from brown bullhead (*Ameiurus nebulosus*) collected from another inland lake, Baseline Lake in Livingston County, MI. Also in May, VHSV was isolated from a gamete sample collected non-lethally from a Lake St. Clair muskellunge during routine broodstock inspections. Just a few weeks later in June, VHSV was isolated from a mortality episode in Lake St. Clair involving primarily smallmouth bass. Stress due to spawning and a concurrent *Flavobacterium columnare* infection likely contributed to the smallmouth bass die-off. In December, VHSV-IVb was isolated from spottail shiners collected from Saginaw Bay, Lake Huron. Also in December, the virus was isolated and confirmed by MSU-AAHL from lake herring (*Coregonus artedi*) collected from areas near the Apostle Islands in Lake Superior, Wisconsin. This constituted the first isolation of VHSV-IVb from Lake Superior.

VHSV was also detected by molecular tests in Lake Superior during this year from fish collected near Paradise and Skanee, Michigan, in the Duluth-Superior Harbor, and at the mouth of the St. Louis River near Superior, Wisconsin. Lake Superior was the last of the five Great Lakes to be invaded by VHSV-IVb. Of the molecular detections, only the yellow perch collected near Paradise, MI were confirmed to have VHSV by genome sequencing done by the United States Geologic Survey (USGS)-Western Fisheries Center. Positive isolations of VHSV were also reported from smallmouth bass in Sturgeon Bay, a smaller bay within Green Bay, Wisconsin (WI DNR Press Release June 12, 2009).

#### 2.1.6. 2010

A total of 357 cases were tested for the virus in 2010, representing 51 fish species (23,522 fish) collected throughout the Upper and Lower Peninsulas of Michigan (Figure 1, Supplementary Table S7). MI DNR’s intensive VHSV-targeted surveillance continued in 2010 with 138 cases from 17 sites in Lakes Michigan, Huron, Erie, and St. Clair; 22 inland lakes and ponds; nine rivers; and one creek. In Lake St. Clair, 25 additional sampling events were conducted on 16 species for ongoing VHSV research. Other testing was performed on fish from inland lakes and rivers (14 cases) due to mortality episodes, unusual lesions, and oil spill responses. Additional fish health inspections were conducted on wild populations in 22 sites for pre-transfer inspections, coolwater broodstock inspections, and general pathogen surveillance. Private facilities submitted 62 cases from nine aquaculture facilities and four bait collection facilities. LSSU-ARL and the six Michigan state fish hatcheries submitted 74 cases of 11 species of captive broodstock and production lots. Feral salmonid returning spawners were also tested for VHSV from four Michigan weirs. VHSV was not detected in any of these cases, nor was it reported from any other areas inside or outside the Great Lakes watershed.

#### 2.1.7. Results Summary 2003–2010

**Figure 6:**
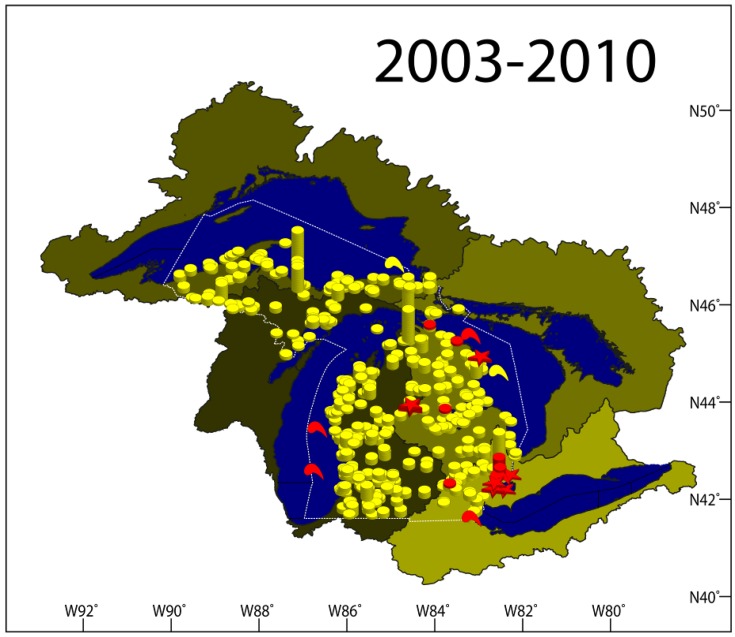
A map of the State of Michigan showing all sites from which samples were collected for viral hemorrhagic septicemia virus testing (2005–2010). Yellow symbols indicate negative sites, red symbols indicate positive sites, red stars indicate positive sites with fish kills, and comma-like shapes indicate sites where macroinvertebrates were sampled. The different color shades outline the watershed of each of the Great Lakes.

**Figure 7:**
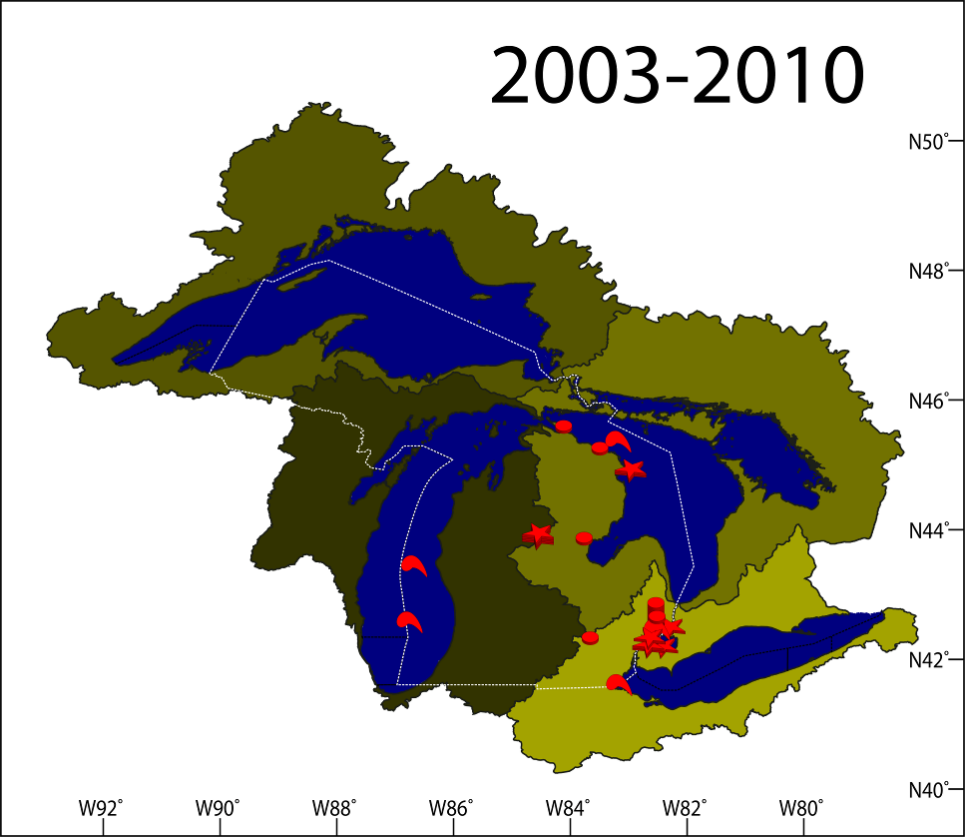
A map of the State of Michigan showing sites where the viral hemorrhagic septicemia virus has been isolated in the period between 2003 and 2010. Red symbols indicate positive sites, red stars indicate positive sites with fish kills, and red comma-like shapes indicate sites with positive macroinvertebrates. The different color shades outline the watershed of each of the Great Lakes.

Following the initial VHSV-IVb isolation in 2003, the multi-year surveillance efforts from 2005 to 2010 were performed on 96,228 fish (1,823 cases, 73 species) covering a considerable number of waterbodies in Michigan’s Upper and Lower Peninsula (Figure 6). The surveillance was designed such that all Great Lakes watersheds, key inland lakes, and propagated fish are tested annually. The surveillance also ensured that enzootic waterbodies, such as Lake St. Clair, were heavily monitored (Figure 7).

Only during 30 cases were we able to isolate VHSV-IVb from fish in Michigan. As displayed in Figure 7, the virus is concentrated on the eastern side of the Lower Peninsula and has never been isolated from fish in Michigan waters of Lakes Michigan or Superior, or from their watersheds within Michigan. This is surprising for three reasons. First, VHSV has been isolated from fish in Lake Michigan and its watershed in both Wisconsin and Illinois. Second, VHSV was isolated on two occasions from *Diporeia* spp. collected from the middle of Lake Michigan. Last, VHSV was isolated from Lake Superior at the Apostle Islands site, which is very close to the Michigan border in the lake. In the same context, Michigan’s Upper Peninsula continues to be VHSV-free despite its proximity to many VHSV-positive sites in the Lower Peninsula. This pattern of virus spreading suggests that the virus may have been transmitted to western Lake Michigan watershed via an external source (e.g., bait or boats used in recreational fishing) and not through the upstream movement of fish infected with the virus. Another possibility would be the efficiency of measures undertaken by state and federal agencies that prevented the virus from getting access to hatcheries and aquaculture facilities, thereby slowing its spread in the wild. In the future, it will be important to use the data from this study to better understand the trafficking of this emerging strain within the basin (Figure 7).

### 2.2. State and Federal Managerial Response to VHSV Emergence

The emergence of VHSV in the Laurentian Great Lakes created initial confusion among state, provincial, and federal fisheries agencies regarding the extent of the response that should be initiated in the Great Lakes region. The mass mortality episodes in 2005 and 2006 prompted the fisheries agencies to develop containment and control measures through the Great Lakes Fishery Commission—Great Lakes Fish Health Committee (GLFHC). Specific recommendations were developed concerning VHSV testing in hatcheries and wild fish surveillance. Biosecurity measures were implemented in hatcheries and for sampling crews which included stringent equipment disinfection and restriction of broodstock collection locations, fish culture and stocking locations and fish transfers. Regulations were also implemented that affected the angler and bait industries, as well as the commercial fish processing industry. Public outreach and key research needs were also addressed by the GLFHC.

In October 2006, these recommendations were adopted by the Great Lakes Fishery Commission—Council of Lake Committees and forwarded onto the fisheries agencies for action. By May 2007, the affected states and the Province of Ontario had implemented all of the regulations that were relevant to each agency and these regulations remain largely in place at this time. The adopted measures significantly limited fisheries management options and likely reduced some fisheries until many of the research gaps were filled in 2011. States where VHSV had not yet been detected put plans into place to implement these measures should they find that waters in their state harbor the virus.

With respect to fish culture, the MI DNR implemented enhanced screening of all wild broodstocks and their eggs, in particular for coolwater fish (e.g., walleye). Coolwater fish, for which no information on the effectiveness of egg disinfection was available, had extensive VHSV testing conducted on pre‑spawn adults, all adults used in spawning, fry, and spring fingerlings. All wild and captive coldwater broodstocks (e.g., salmon) continued to be tested for VHSV as they have been since the 1970s. All eggs are disinfected using iodophor at 50 ppm for 30 minutes during water hardening, then for 10 minutes at 100 ppm just prior to moving the eggs into the hatchery. While no real adjustment was made in coldwater fish production programming, significant changes occurred for coolwater fish production. Wild broodstock use was restricted, which included the termination of northern pike fry rearing as there is no feasible timely method to test northern pike fry prior to stocking. The MI DNR began using only unaffected inland wild broodstocks for their programs. Hatcheries were reconfigured to reduce VHSV transmission risk; discharge into the environment from rearing ponds was only authorized if the hatchery was located in a VHSV-positive zone. Fish stocking was limited to land‑locked bodies of water or to waters already known to be affected. 

These restrictions caused a reduction of approximately 80% in walleye stocking and, after several years, began to reduce fisheries in some locations reliant on this stocking. Restrictions on the walleye program were greatly lifted in 2011 as a result of research done on walleye susceptibility, VHSV transmission, and the effectiveness of disinfection protocols. The disinfection methods used for salmonids were fully implemented for walleye as research by the USGS Upper Midwest Environmental Center showed these methods to be effective against superficial infections of VHSV on walleye and northern pike [47].

MI DNR implemented restrictive regulations for anglers and for the bait industry, which relies on wild caught baitfish for a substantial amount of its sales. For the first time, a baitfish certification program was put into effect for wild-caught baitfish species that were known to be susceptible to VHSV. Imported baitfish were required to be certified free of VHSV before they were brought into the state. Certified VHSV-negative baitfish could be used statewide for 14 days and uncertified bait could only be used in specific areas and only for 3 days. The movement of live fish from one waterbody to another was prohibited and anglers and boaters were required to empty live wells and bilges upon leaving a body of water. The use of salmon eggs as chum to enhance the catch rates for anglers was prohibited, although this regulation is under review at this time and will likely be modified to allow the use of disinfected VHSV-free eggs for this purpose. Finally, to bring the public into partnership on disease management, all of the jurisdictions implemented extensive public outreach efforts across agencies, including the Great Lakes Fishery Commission, MI DNR, the U.S. Fish and Wildlife Service and USDA-APHIS. Wild fish transfers by MI DNR personnel could only be done with certified VHSV-free fish, and required additional testing to be completed prior to any transfer. Initially, Great Lakes research vessel movement between lakes was restricted, though by 2008 this restriction was replaced with enhanced disinfection of all vessels and gear. A review of fishway operations in Michigan was conducted, but due to risk of interfering with salmonid movement from the Great Lakes, fishways remained open. It should be noted that most of these fishways are concentrated in Lake Michigan tributary streams, and fish from Michigan waters of Lake Michigan have not been found with VHSV. Commercial fishing operations were reviewed and most of the processing plants for these fisheries were located on the same Great Lake from which the fish were captured. All of the processing plants were found to handle their wastes with a low risk of VHSV transmission.

All of the Great Lakes fisheries agencies and the GLFHC recognized the significant information gaps they had on this new emergent pathogen. As a group, they successfully advocated for and obtained additional funds from a range of sources to address iodophor disinfection effectiveness, pathogen susceptibility, likelihood of vertical transmission, rapid VHSV testing methods, and understanding the disease course in susceptible species. During the period from 2007 to 2011, MI DNR conducted VHSV surveillance in approximately 175 locations using 6,000 to 10,000 fish annually, partially funded by USDA-APHIS. Similar, although usually lesser, efforts were also conducted by many of the other Great Lakes fisheries agencies. By 2011, these efforts began to provide key information to Great Lakes fisheries managers which allowed them to refine their containment approaches and make reasonable changes in their VHSV management strategies.

Parallel to the regional fisheries agencies response, U.S. federal agencies implemented additional containment measures that targeted the movement of fish from the Great Lakes VHSV infected region. In October 2006, USDA-APHIS issued an emergency order that prohibited the interstate transfer of live fish of 37 species of both game and baitfish among the Great Lakes states and banned importation of live fish from the Canadian Provinces of Quebec and Ontario. VHSV-susceptible species of live fish originating outside of the prohibited areas were allowed to move through or to the affected states and provinces. The emergency order was released pursuant to the U.S. Animal Health Protection Act with the stated intent “*to prevent the spread of viral hemorrhagic septicemia (VHS) into aquaculture facilities*” [48].

As the result of additional information on VHSV and after meeting with affected parties in the Great Lakes region, the order was amended by USDA-APHIS in November 2006. The amended emergency order still banned the movement of live fish on the susceptible species list from Quebec and Ontario into the United States, except for salmonids moved using Title 50 provisions, Code of Federal Regulations, Sections 16.13 (a) (3) and 16.13 (b). The conditions for the interstate movement of VHSV-susceptible species varied depending on whether the live fish are being transported for slaughter, research or other purposes. If fish were moved for slaughter; they must be intended for human consumption and slaughtered at a facility that discharges waste water into a municipal sewage system, a non-discharging, settling pond, or a discharging settling pond that first disinfects its wastes. The slaughter facility must compost offal and carcasses and must have proper USDA transport documentation. To move VHSV-susceptible species for other purposes between states, the fish must be transported with documentation (from appropriate state, tribal, or federal authorities of aquatic animal health) stating that the fish have tested negative for VHSV using appropriate American Fisheries Society Blue Book or OIE testing methods.

A further significant emergency order amendment occurred in April 2007. This amendment allowed the international movement of angler-caught live fish that were to be released as long as these activities occurred in the same geographically distinct water. Other minor amendments were put into place in April 2008. USDA-APHIS began the VHSV rule promulgation process in 2008 and as this process is still ongoing, the emergency order provisions are the U.S. federal regulations at this time.

### 2.3. Research Efforts Made to Better Understand VHSV-IVb Biology and Ecology

Several landmark studies on the pathogenesis, ecology, diagnosis, and risk factors of VHSV-IVb were initiated as a result of the virus emergence in the Great Lakes basin. In the area of VHSV pathogenesis, Kim and Faisal [20,21,22,49] designed a series of experimental infection studies with a range of Great Lakes fish species. These studies provided evidence that the new VHSV sublineage can cause the same clinical signs and pathology observed in field samples during fish kill episodes, thereby fulfilling River’s postulates. This is important since infected wild fish often harbored concurrent infections and/or were sampled during or after stressful events, such as temperature fluctuations or spawning periods. Experimental infection studies also confirmed field observations that VHSV-IVb has a wide host range [20,21].

Experimental studies also reported on interspecies differences in susceptibility to VHSV-IVb that resulted in classification of representative Great Lakes fish species into highly susceptible, moderately susceptible, and resistant. For example, the median lethal dose of infection (LD_50_) of VHSV-IVb by intraperitoneal injection was as little as 2.2 plaque forming units (pfu) for muskellunge, which is the lowest ever reported for any fish species with any of the fish pathogenic viruses, attesting to the higher virulence of the emerging VHSV strain [22]. Salmonids, such as lake trout, Atlantic salmon, chinook salmon, and coho salmon are relatively resistant to infection [20,21]. However, Weeks *et al.* [50] demonstrated that one of the salmonid species, the lake herring (*Coregonus artedii*), is highly susceptible to VHSV.

Experimental infection studies also shed light on important aspects of VHSV-IVb disease ecology. Depending on the dose and route of exposure, the disease course of VHSV-IVb, like that for other VHSV genotypes, can run one of three forms: acute, subacute, or chronic [22]. Even with one of the most susceptible fish species, the muskellunge, some individual fish can recover and survive from VHSV infection. In this context, Kim and Faisal [49] demonstrated that fish surviving a single encounter with VHSV-IVb continue to shed the virus in great concentration into the surrounding waters for an extended period of time (15 weeks post-infection). The authors also found that if surviving fish that ceased shedding the virus were exposed to stress (e.g., handling stress), virus shedding can resume for up to an additional 15 weeks. These findings suggest that recovered fish play an important role in maintaining VHSV in the ecosystem.

Macroinvertebrates also appear to play an active role in VHSV-IVb ecology. As stated above, VHSV was isolated from the amphipod *Diporeia* spp. collected from sediments at depths of ~150 meters. Amphipods such as *Diporeia* spp. may serve as a reservoir for VHSV, as they are an important food source to many Great Lakes fish species. This was the first report of any fish‑pathogenic virus being associated with amphipods. In the same context, leeches may act as vectors for VHSV transmission to host fish. Leeches such as *M. lugubris* penetrate mucus membranes causing ulcers on parasitized fish which could serve as potential portals of entry for VHSV [51].

A number of diagnostic assays with increased sensitivity and specificity have been developed for the direct and indirect diagnosis of VHSV. For example, Hope *et al.* [52] developed a single step quantitative reverse-transcriptase polymerase chain reaction (qRT-PCR) assay that amplifies a 100‑base-pair segment from both the genomic negative strand and the mRNA positive strand of the N gene of VHSV-IVb. This assay proved to be both rapid and sensitive with an analytical capability of detecting a single copy of viral RNA. Hope *et al.* then used the enhanced diagnostic assay to detect VHSV in 1,428 fish caught from the Great Lakes region between 2006 and 2007. The new qRT-PCR assay detected viral RNA in 24% of the fish whereas only 5% were positive by cell culture.

Bain *et al.* [53] used the newly developed qRT-PCR assay [52] to determine the spread of the virus within the Great Lakes basin. The virus was found in 21 out of 30 sites tested. These findings indicated that VHSV is widely dispersed in the basin and that its distribution is not correlated with shipping activities or ports. Most recently, Garver *et al.* [54] developed and validated another qRT-PCR assay that amplifies a sequence from representative isolates of all VHSV genotypes (I, II, III and IV). This assay detects as few as 100 copies of VHSV nucleoprotein RNA with high specificity. When the newly developed assay was evaluated on experimentally infected Atlantic salmon, it revealed a diagnostic sensitivity of ≥93% and specificity of 100%.

An indirect diagnostic assay for the detection of VHSV-IVb antibodies has also recently been reported [55]. Millard and Faisal used a complement-dependent 50% plaque neutralization assay to detect and titrate neutralizing antibodies against VHSV-IVb in sera of experimentally infected muskellunge. The assay was then applied to compare the neutralizing antibody responses of 13 fish species collected from Lake St. Clair [56]. Neutralizing antibodies were detected in four Lake St. Clair fish species, with muskellunge and northern pike having the highest seroprevalences. On most sampling occasions, antibodies were detected even when the virus was not, suggesting that these fish have survived a relatively recent VHSV infection. The authors suggested the incorporation of serological testing into current VHSV surveillance efforts in the Great Lakes to determine the immune status of populations and to provide a more thorough understanding of virus distribution.

The genetic diversity of VHSV-IVb isolates was determined by comparing partial glycoprotein (G) gene sequences (669 nucleotides) of 108 isolates collected between 2003 and 2009. These isolates represented 31 species and 37 sites throughout the Great Lakes basin. Overall, the genetic diversity of VHSV in the Great Lakes region was found to be extremely low, with a maximum of 1.05% sequence dissimilarity within the 669 nucleotides region [42]. The 108 isolates were distributed among 11 unique sequence types (vcG001–vcG011) and there appears to be an association between sequence type and geographic location within the basin. For example, the vcG001 sequence type, representative of the index isolate reported by Elsayed *et al.* [38], was the most widespread in the basin, while type vcG002 was more prevalent in the easternmost sub-regions of the Great Lakes basin and the St. Lawrence Seaway [42].

Based on experience with VHSV in Europe and North America, an international panel of fish health experts identified risk factors that have played a role in the emergence and spread of VHSV in the Great Lakes basin. These factors included the presence of VHSV-susceptible species, water temperature, hydrologic connectivity and proximity to known VHSV-positive areas, untested shipments of live or frozen fish from infected zones, insufficient regulatory infrastructure for fish health oversight, and uncontrolled exposure to fomites associated with boats and equipment or fish wastes from known VHSV-positive areas [17]. These assumptions were proven correct in a number of situations. When Kane-Sutton *et al.* [44] sampled spawning yellow perch during the spring, summer, and fall of 2007 and 2008 in the central basin of Lake Erie, they determined that VHSV infection is widespread only during the spawning season of this species. Successful isolation of the virus could only occur during a three week period starting in the last week of spawning to early June. The authors attributed this to water temperature (between 12 and 18 °C), spawning stress, and the higher yellow perch densities during spawning. This finding was corroborated by Eckerlin *et al.* [57] who found a strong temporal variation in prevalence of VHSV-IVb with peaks in prevalence corresponding to the smallmouth bass spawning period and a temperature range of 10–14 °C. Data from Michigan corroborate those of Kane-Sutton *et al.* [44] and Eckerlin *et al.* [57] in that the majority of VHSV mortalities took place in the spring and early summer (Table 1), confirming water temperature and spawning activity are major risk factors for VHSV disease.

Data from other VHSV genotypes also point to stress, age, and water temperature as potential controlling factors for VHSV infection. At temperatures between 9 and 12 °C, VHSV is most pathogenic [58], and this pathogenicity is greatly diminished at or above 20 °C [59]. Fish age at infection was found to determine the course and outcome of VHSV infection. Infected fry and juvenile fish experience much higher mortality rate than older fish [60,61,62]. On the other hand, fish at any age can become infected [63]. While VHSV-IVb shares many characteristics with other VHSV strains, the wide host range of this sublineage [64] suggests that some of its biological characteristics may be different, a matter that requires additional studies on the virulence mechanisms and factors that predispose fish to infection.

## 3. Materials and Methods

### 3.1. Fish and Sample Processing

Between the years of 2005 and 2010, a total of 96,228 fish representing 73 species, were collected from Michigan waters and tested for the presence of VHSV. Fish were collected from Lakes St. Clair, Erie, Huron, Michigan, and Superior, 120 inland lakes and ponds, 35 rivers, 14 creeks, six state-operated fish hatcheries, six gamete collection weirs, and 26 private aquaculture farms and bait collection facilities. All sites were within the geographical boundaries of the State of Michigan. The fish were collected by field veterinarians, MI DNR personnel, or personnel of the MSU-AAHL. Wild fish were collected by a variety of methods including various types of netting (e.g., trawl, seine, trap, fyke and hand nets), electrofishing, and angling. The fish were delivered to the MSU-AAHL either alive, freshly dead on ice, or frozen at −80 °C. In some cases, fish arrived frozen at −20 °C if collecting entities did not have access to −80 °C temperatures. Specific dates, locations, species, and numbers of fish sampled are displayed in Supplementary Tables S1–S7 and Table 1.

Once received, fish were examined for the presence of lesions. If delivered alive, the fish were sacrificed by an overdose (0.25 mg mL^−1^) of tricaine methanesulfonate (MS-222; Argent Chemical Laboratories, Redmond, WA, USA). Blood was collected from select live fish by caudal venipuncture, and total length and weight were recorded. Fish were then dissected under aseptic conditions and examined for the presence of internal lesions. Kidney and spleen tissues were collected for virus isolation. Samples were processed within 24 hours of collection or frozen immediately at −20 °C until processing could take place.

Tissues were diluted 1:4 (weight to volume) with Earle’s salt-based minimal essential medium (EMEM; Invitrogen, Carlsbad, CA, USA) supplemented with 10% BD Bacto^TM^ tryptose phosphate broth (TPB; Becton, Dickinson and Company, Sparks, MD, USA), 12 mM tris buffer (Sigma-Aldrich, St. Louis, MO, USA), penicillin (100 IU mL^−1^) and streptomycin (100 µg mL^−1^; Invitrogen), gentamicin sulfate (0.1 mg mL^−1^; Sigma-Aldrich), and amphotericin B (2.5 µg mL^−1^; Lonza, Walkersville, MD, USA). Samples were then homogenized in a Biomaster Stomacher (Wolf Laboratories Ltd. Pocklington, York, UK) at a high speed for 4 minutes, followed by centrifugation at 5,000 rpm for 30 minutes at 4 °C. Supernatants were then used for virus isolation on susceptible cell lines.

### 3.2. Cell Lines

The cell lines *Epithelioma papulosum cyprini* (EPC) [65], fathead minnow (FHM) [66], and chinook salmon embryo (CHSE-214) [67] were used throughout this study. Cell lines were maintained in 150 cm^2^ tissue culture flasks (Corning, Lowell, MA, USA) at 25 °C (EPC and FHM) and 15 °C (CHSE). Growth medium for EPC cells was MEM-10SB, which is a formulation of EMEM supplemented with 2 mM L-glutamine (Invitrogen), 10% fetal bovine serum (FBS; Hyclone, Logan, UT, USA), 10% TPB, and buffered with sodium bicarbonate (7.5% w/v; Sigma-Aldrich). FHM cells were grown cultured in MEM with Hanks’ salts (Invitrogen), L-glutamine (2 mM) and 10% FBS. CHSE cells were grown in Eagle’s MEM (with Earle’s salts, nonessential amino acids, and sodium pyruvate; ATCC, Manassas, VA, USA), L-glutamine (2 mM) and 10% FBS. 

### 3.3. Virus Isolation

Virus isolation attempts followed the protocols outlined in the American Fisheries Society Blue Book [68] with slight modifications. Cells were allowed to grow on flat bottom 96-well plates, 24-well plates or 25 cm^2^ flasks (all from Corning). The growth medium used for plates (100 µL per well) was MEM-5-T, a formulation for open cultures made of EMEM supplemented with 5% FBS, 10% TPB, 2 mM L-glutamine, penicillin (100 IU mL^−1^), streptomycin (100 µg mL^−1^), gentamycin sulfate (0.1 mg mL^−1^), amphotericin B (2.5 µg mL^−1^) and 12 mM tris buffer. Tissue homogenate supernatants were inoculated (30 µL per well) in triplicate on well plates or 1 mL per flask. After inoculation of 25 cm^2^ closed flasks, growth media was replaced with 4 mL of maintenance media (with only 2% FBS). Inoculated cell cultures were incubated at 15 °C and monitored for the formation of cytopathic effects (CPE) for up to 14 days. A second passage was performed for each sample and observed for 14 additional days.

### 3.4. Virus Identification and PCR Confirmation

When CPE was present, total RNA was extracted from inoculated cell culture supernatant using Trizol® LS Reagent (Invitrogen) or the QIAamp® Viral RNA Mini Kit (Qiagen Inc., Valencia, CA, USA) following manufacturer’s instructions. Reverse transcription was performed using AffinityScript™ Multiple Temperature Reverse Transcriptase (Agilent Technologies, Santa Clara, CA, USA) following the manufacturer’s manual. As per the Office of International Epizootics recommendations (OIE 2006), a primer set was used to amplify an 811 base pair sequence of the nucleocapsid gene: 5'-GGG-GAC-CCC-AGA-CTG-T-3' (forward primer) and 5'-TCT-CTG-TCA-CCT-TGA-TCC-3' (reverse primer). Viral RNA (5 μL), 50 pmol of each primer, 25 μL of Choice^TM^*Taq* Mastermix DNA Polymerase (Denville Scientific Inc., South Plainfield, NJ, USA) or GoTaq® Green Master Mix (Promega, Fitchburg, WI, USA) and nuclease-free water were combined in each reaction tube to create a final volume of 50 μL. An inactivation of the reverse transcriptase was performed in a Mastercycler Personal Thermal Cycler (Eppendorf, Hamburg, Germany) by subjecting the mixture to 94 °C for 2 minutes, followed by 30 cycles of PCR (denaturation for 30 s at 94 °C, annealing for 30 s at 52 °C, and polymerization at 68 °C for 1 minute). The polymerization was finalized by maintaining the mixture for a period of 7 minutes at 68 °C. The products were visualized by UV illumination following gel electrophoresis.

Representative isolates were sent for confirmation and additional sequence analysis to Dr. James Winton at the United States Geological Survey (Western Fisheries Research Center, Seattle, WA, USA), and Dr. Janet Warg (National Veterinary Services Laboratories, Ames, IA, USA).

## 4. Conclusions

Findings of multiyear VHSV-IVb-targeted surveillance and research efforts have shed light on important aspects of VHSV-IVb epidemiology and biology. The novel VHSV sublineage infects, and has the potential to cause losses in, a large number of ecologically and recreationally important fish species in the Great Lakes region, most notably muskellunge, bluegill, yellow perch, and freshwater drum. Regulations put in place by federal and state agencies have likely minimized the virus spread since VHSV has only been detected in a few inland lakes, and its spread within the five Great Lakes has been relatively slow. In Michigan, the virus-positive sites seem to be focused around Lake St. Clair and, to a lesser extent, Lake Huron and its watershed. It seemed that the most significant die-off events were evident as the virus initially moved into new bodies of water where it encountered a large population of naïve hosts. It is also possible that the die-off events prompted the discovery of the virus in those areas. Mortality events may have been triggered or exacerbated by stressful events such as spawning, high fish densities, and temperature fluctuations, as seen in Lake St. Clair, where VHSV has persisted over eight years, periodically causing mortalities.

The fact that VHSV has been isolated from western Lake Michigan, but not from eastern Lake Michigan, points to the possibility that the virus has not spread westward, but rather eastwards. This observation underscores the efforts made in the State of Michigan to hinder the spread of the virus in both directions (westward and eastward). Also, spread to inland lakes throughout the state seems to have occurred more slowly than expected in 2006, also pointing to successful efforts by fisheries agencies, anglers and boaters. The isolation of VHSV from leeches and *Diporeia* spp. attests to the complexity of VHSV transmission and spread between different biotic components of the ecosystem. Further studies on how the virus is maintained in ecosystems are warranted, as are continued efforts to keep the virus from entering state hatcheries and commercial aquaculture facilities in Michigan and elsewhere.

## Supplementary Files

Supplementary File 1: PDF-Document (PDF, 357 KB)
