# Supplementary material for: Spread of the Emerging Viral Hemorrhagic Septicemia Virus Strain, Genotype IVb, in Michigan, USA
Source: Viruses. 2012 May 3;4(5):734–60. doi: 10.3390/v4050734 (PMC3386630; doi:10.3390/v4050734)
Supplement: Supplementary File 1: — PDF-Document (PDF, 357 KB) [file viruses-04-00734-s001.pdf]

Supplementary Table S1. Scientific names of fish species tested for viral hemorrhagic septicemia virus in this study.

**Common Name**	**Scientific Name**
alewife	*Alosa pseudoharengus*
American brook lamprey	*Lampetra appendix*
Atlantic salmon	*Salmo salar*
black crappie	*Pomoxis nigromaculatus*
blacknose dace	*Rhinichthys atratulus*
bluegill	*Lepomis macrochirus*
bluntnose minnow	*Pimephales notatus*
brook stickleback	*Culaea inconstans*
brook trout	*Salvelinus fontinalis*
brown bullhead	*Ameiurus nebulosus*
brown trout	*Salmo trutta*
central stoneroller	*Campostoma anomalum*
channel catfish	*Ictalurus punctatus*
chinook salmon	*Oncorhynchus tshawytscha*
coho salmon	*Oncorhynchus kisutch*
common carp	*Cyprinus carpio*
common shiner	*Luxilus cornutus*
common white sucker	*Catostomus commersoni*
creek chub	*Semotilus atromaculatus*
emerald shiner	*Notropis atherinoides*
fathead minnow	*Pimephales promelas*
freshwater drum	*Aplodinotus grunniens*
gizzard shad	*Dorosoma cepedianum*
golden rainbow trout	*Oncorhynchus mykiss*
golden redhorse sucker	*Moxostoma erythrurum*
golden shiner	*Notemigonus crysoleucas*
green sunfish	*Lepomis cyanellus*
horneyhead chub	*Nocomis biguttatus*
hybrid bluegill	*Lepomis macrochirus* X *Lepomis cyanellus*
johnny darter	*Etheostoma nigrum*
koi	*Cyprinus carpio*
lake herring	*Coregonus artedi*
lake sturgeon	*Acipenser fulvescens*
lake trout	*Salvelinus namaycush*
lake whitefish	*Coregonus clupeaformis*
largemouth bass	*Micropterus salmoides*

**Common Name**	**Scientific Name**
longnose gar	*Lepisosteus osseus*
longnose sucker	*Catostomus catostomus*
mottled sculpin	*Cottus bairdii*
muskellunge	*Esox masquinongy*
northern pike	*Esox lucius*
northern redbelly dace	*Phoxinus eos*
pumpkinseed	*Lepomis gibbosus*
quillback	*Carpiodes cyprinus*
rainbow darter	*Etheostoma caeruleum*
rainbow smelt	*Osmerus mordax*
rainbow trout	*Oncorhynchus mykiss*
redear sunfish	*Lepomis microlophus*
rockbass	*Ambloplites rupestris*
round goby	*Neogobius melanostomus*
sand shiner	*Notropis stramineus*
sculpin	*Cottus* sp.
sea lamprey	*Petromyzon marinus*
shorthead redhorse	*Moxostoma macrolepidotum*
silver redhorse	*Moxostoma anisurum*
smallmouth bass	*Micropterus dolomieu*
smelt	*Osmerus eperlanus*
splake	*Salvelinus namaycush* X *Salvelinus fontinalis*
spotfin shiner	*Notropis spilopterus*
spottail shiner	*Notropis hudsonius*
steelhead	*Oncorhynchus mykiss*
stickleback	species not recorded
trout perch	*Percopsis omiscomaycus*
walleye	*Sander vitreus*
warmouth	*Lepomis gulosus*
western blacknose dace	*Rhinichthys obtusus*
white bass	*Morone chrysops*
white crappie	*Pomoxis annularis*
white perch	*Morone americana*
yellow bullhead	*Ameiurus nebulosus*
yellow perch	*Perca flavescens*

Supplementary Table S2. List of viral hemorrhagic septicemia testing performed in Michigan in 2005 showing site locations, fish species, specimen tested, and test results. K: kidneys, S: spleen, OF: ovarian fluid.

Date	Location	Species & Rearing Condition	Tissue	VHSV Results
1/3/05	Oden State Fish Hatchery	hatchery spawning brown trout	OF	negative
1/7/05	Lake Huron, DeTour Village	wild lake whitefish	K/S	negative
1/9/05	Platte River State Fish Hatchery	hatchery propagated chinook salmon	K/S	negative
1/11/05	Marquette State Fish Hatchery	hatchery fingerling lake trout	K/S	negative
	Marquette State Fish Hatchery	hatchery fingerling splake	K/S	negative
1/12/05	Marquette State Fish Hatchery	hatchery fingerling brook trout	K/S	negative
1/21/05	Marquette State Fish Hatchery	hatchery brook trout	K/S	negative
1/26/05	Oden State Fish Hatchery	hatchery fingerling brown trout	K/S	negative
1/27/05	Platte River State Fish Hatchery	hatchery propagated fingerling coho salmon	K/S	negative
	Platte River State Fish Hatchery	hatchery propagated fingerling chinook salmon	K/S	negative
	Harrietta State Fish Hatchery	hatchery fingerling brown trout	K/S	negative
	Harrietta State Fish Hatchery	hatchery fingerling rainbow trout	K/S	negative
2/2/05	Wolf Lake State Fish Hatchery	hatchery propagated fingerling chinook salmon	K/S	negative
2/3/05	Wolf Lake State Fish Hatchery	hatchery propagated fingerling steelhead	K/S	negative
2/4/05	Wolf Lake State Fish Hatchery	hatchery propagated fingerling chinook salmon	K/S	negative
	Lake Michigan, Big Bay de Noc	wild lake whitefish	K/S	negative
2/7/05	Marquette State Fish Hatchery	hatchery fingerling splake	K/S	negative
	Marquette State Fish Hatchery	hatchery fingerling brook trout	K/S	negative
	Marquette State Fish Hatchery	hatchery fingerling lake trout	K/S	negative
2/8/05	Thompson State Fish Hatchery	hatchery propagated fingerling chinook salmon	K/S	negative
	Thompson State Fish Hatchery	hatchery propagated fingerling steelhead	K/S	negative
	Thompson State Fish Hatchery	hatchery fingerling brown trout	K/S	negative
	Thompson State Fish Hatchery	hatchery fingerling Atlantic salmon	K/S	negative
	Thompson State Fish Hatchery	hatchery fingerling rainbow trout	K/S	negative
	Lake Superior State University	hatchery fingerling Atlantic salmon	K/S	negative
	Lake Superior State University	hatchery brook trout	K/S	negative

Supplementary Table S2. *Cont.*

Date	Location	Species & Rearing Condition	Tissue	VHSV Results
2/9/05	Oden State Fish Hatchery	hatchery fingerling rainbow trout	K/S	negative
2/12/05	Lake Michigan, Naubinway	wild lake whitefish	K/S	negative
2/17/05	Marquette State Fish Hatchery	hatchery brook trout	K/S	negative
2/23/05	Wolf Lake State Fish Hatchery	hatchery propagated chinook salmon	K/S	negative
3/3/05	Marquette State Fish Hatchery	hatchery lake trout	K/S	negative
3/4/05	Oden State Fish Hatchery	hatchery spawning rainbow trout	OF	negative
3/17/05	Oden State Fish Hatchery	hatchery fingerling brown trout	K/S	negative
	Oden State Fish Hatchery	hatchery fingerling rainbow trout	K/S	negative
3/21/05	Lake Huron, Cheboygan	wild lake whitefish	K/S	negative
3/29/05	Harrietta State Fish Hatchery	hatchery rainbow trout	K/S	negative
4/6/05	Tittabawasee River, Midland County	wild spawning walleye	K/S, OF/milt	negative
4/8/05	Muskegon River, Newaygo County	wild walleye	K/S	negative
4/11/05	Little Manistee River Weir	feral spawning steelhead	K/S, OF/milt	negative
	Platte River State Fish Hatchery	hatchery propagated chinook salmon	K/S	negative
4/20/05	Oden State Fish Hatchery	hatchery rainbow trout	K/S	negative
4/22/05	Oden State Fish Hatchery	hatchery brown trout	K/S	negative
5/10/05	Eel Lake, Gogebic County	wild muskellunge	K/S	negative
	Oden State Fish Hatchery	hatchery rainbow trout	K/S	negative
	Oden State Fish Hatchery	hatchery brown trout	K/S	negative
	Aquaculture Facility 22, Wexford County	aquaculture-raised brook trout	K/S	negative
5/11/05	Thompson State Fish Hatchery	hatchery propagated chinook salmon	K/S	negative
	Big Lake, Oakland County	wild largemouth bass	K/S	negative
	Big Lake, Oakland County	wild bluegill	K/S	negative
	Big Lake, Oakland County	wild minnow sp.	K/S	negative
5/17/05	Marquette State Fish Hatchery	hatchery brook trout	K/S	negative
	Thompson State Fish Hatchery	hatchery brown trout	K/S	negative

Supplementary Table S2. *Cont.*

Date	Location	Species & Rearing Condition	Tissue	VHSV Results
5/18/05	Woodland Lake, Livingston County	wild largemouth bass	K/S	negative
	Woodland Lake, Livingston County	wild black crappie	K/S	negative
	Woodland Lake, Livingston County	wild bluegill	K/S	negative
	Woodland Lake, Livingston County	wild minnow sp.	K/S	negative
5/19/05	Marquette State Fish Hatchery	hatchery brook trout	K/S	negative
5/20/05	Lake St. Clair, Michigan waters	wild muskellunge	K/S	negative
	Lake St. Clair, Michigan waters	wild walleye	K/S	negative
5/23/05	Lake St. Clair, Michigan waters	wild walleye	K/S	negative
	Lake St. Clair, Michigan waters	wild northern pike	K/S	negative
5/24/05	Lake Michigan, Big Bay de Noc	wild lake whitefish	K/S	negative
	Platte River State Fish Hatchery	hatchery propagated coho salmon	K/S	negative
5/25/05	Lake St. Clair, Michigan waters	wild muskellunge	K/S	negative
	Lake St. Clair, Michigan waters	wild channel catfish	K/S	negative
	Lake St. Clair, Michigan waters	wild smallmouth bass	K/S	negative
6/1/05	Lake Michigan, Naubinway	wild lake whitefish	K/S	negative
	Pine Lake, Barry County	wild largemouth bass	K/S	negative
	Pine Lake, Barry County	wild bluegill	K/S	negative
	Pine Lake, Barry County	wild walleye	K/S	negative
6/2/05	Lake Huron, Cheboygan	wild lake whitefish	K/S	negative
6/7/05	Lake Huron, DeTour Village	wild lake whitefish	K/S	negative
6/8/05	Murray Lake, Kent County	wild largemouth bass	K/S	negative
	Murray Lake, Kent County	wild bluegill	K/S	negative
	Murray Lake, Kent County	wild black crappie	K/S	negative
	Murray Lake, Kent County	wild minnow sp.	K/S	negative
6/9/05	Murray Lake, Kent County	wild golden redhorse sucker	K/S	negative
	Lake Superior State University	hatchery Atlantic salmon	K/S	negative

Supplementary Table S2. *Cont.*

Date	Location	Species & Rearing Condition	Tissue	VHSV Results
6/21/05	Thompson State Fish Hatchery	hatchery fingerling walleye	K/S	negative
	Gourdneck Lake, Kalamazoo County	wild largemouth bass	K/S	negative
	Gourdneck Lake, Kalamazoo County	wild bluegill	K/S	negative
	Gourdneck Lake, Kalamazoo County	wild brown bullhead	K/S	negative
7/7/05	Marquette State Fish Hatchery	hatchery lake trout	K/S	negative
	Lake Superior State University	hatchery Atlantic salmon	K/S	negative
	Thompson State Fish Hatchery	hatchery brown trout	K/S	negative
7/12/05	Hunt Creek, spring-fed, Montmorency County	wild brown trout	K/S	negative
	Hunt Creek, spring-fed, Montmorency County	wild rainbow trout	K/S	negative
	Gilchrist Creek, spring-fed, Montmorency County	wild brown trout	K/S	negative
	Gilchrist Creek, spring-fed, Montmorency County	wild mottled sculpin	K/S	negative
	Gilchrist Creek, spring-fed, Montmorency County	wild blacknose dace	K/S	negative
7/19/05	Lake Huron, Bay City	wild walleye	K/S	negative
7/26/05	Marquette State Fish Hatchery	hatchery fingerling lake trout	K/S	negative
	Marquette State Fish Hatchery	hatchery splake	K/S	negative
7/28/05	Thompson State Fish Hatchery	hatchery brown trout	K/S	negative
8/2/05	Silver Creek, Silver Creek Trout Pond, Cass County	wild brown trout	K/S	negative
	Silver Creek, Silver Creek Trout Pond, Cass County	wild mottled sculpin	K/S	negative
	Silver Creek, Silver Creek Trout Pond, Cass County	wild common white sucker	K/S	negative
	Silver Creek, Silver Creek Trout Pond, Cass County	wild blacknose dace	K/S	negative
8/4/05	Big Bass Lake, Otsego County	wild largemouth bass	K/S	negative
	Harrietta State Fish Hatchery	hatchery fingerling brown trout	K/S	negative
	Harrietta State Fish Hatchery	hatchery rainbow trout	K/S	negative
8/9/05	Lake Michigan, Big Bay de Noc	wild lake whitefish	K/S	negative
	Thompson State Fish Hatchery	hatchery Atlantic salmon	K/S	negative
	Lake Superior State University	hatchery fingerling Atlantic salmon	K/S	negative
8/11/05	Oden State Fish Hatchery	hatchery fingerling brown trout	K/S	negative
	Oden State Fish Hatchery	hatchery fingerling rainbow trout	K/S	negative

Supplementary Table S2. *Cont.*

Date	Location	Species & Rearing Condition	Tissue	VHSV Results
8/18/05	Marquette State Fish Hatchery	hatchery fingerling brook trout	K/S	negative
	Marquette State Fish Hatchery	hatchery fingerling lake trout	K/S	negative
	Marquette State Fish Hatchery	hatchery fingerling splake	K/S	negative
	Fawn Lake, Schoolcraft County	wild channel catfish	K/S	negative
8/23/05	Wolf Lake State Fish Hatchery	hatchery muskellunge	K/S	negative
	Wolf Lake State Fish Hatchery	hatchery propagated steelhead	K/S	negative
	Lake Michigan, Naubinway	wild lake whitefish	K/S	negative
	Lake Huron, DeTour Village	wild lake whitefish	K/S	negative
8/24/05	Canadian Lakes, Mecosta County	wild largemouth bass	K/S	negative
8/26/05	Lake Huron, Cheboygan	wild lake whitefish	K/S	negative
8/31/05	Wolf Lake State Fish Hatchery	hatchery fingerling lake sturgeon	K/S	negative
9/1/05	Lake Superior, Ontonagon	wild brook trout	K/S	negative
	Lake Superior, Ontonagon	wild mottled sculpin	K/S	negative
	Lake Superior, Ontonagon	wild blacknose dace	K/S	negative
	Lake Superior, Ontonagon	wild common white sucker	K/S	negative
9/2/05	Oden State Fish Hatchery	hatchery brown trout	K/S	negative
9/7/05	Austin Lake, Kalamazoo County	wild largemouth bass	K/S	negative
	Austin Lake, Kalamazoo County	wild yellow perch	K/S	negative
	Austin Lake, Kalamazoo County	wild bluegill	K/S	negative
	Austin Lake, Kalamazoo County	wild brown bullhead	K/S	negative
	Mason County Walleye Pond, Mason County	wild walleye	K/S	negative
9/9/05	Wolf Lake State Fish Hatchery	hatchery fingerling lake sturgeon	K/S	negative
9/14/05	Lake Huron, Saginaw Bay	wild yellow perch	K/S	negative
	Lake Huron, Saginaw Bay	wild walleye	K/S	negative
9/22/05	Taylor Lake, Barry County	wild black crappie	K/S	negative
	Taylor Lake, Barry County	wild bluegill	K/S	negative
	Sun Lake, Gogebic County	wild largemouth bass	K/S	negative
	Sun Lake, Gogebic County	wild bluegill	K/S	negative

Supplementary Table S2. *Cont.*

Date	Location	Species & Rearing Condition	Tissue	VHSV Results
9/28/05	Bob Lake, Houghton County	wild yellow perch	K/S	negative
	Gaylord Lake, Gogebic County	wild largemouth bass	K/S	negative
9/30/05	Oden State Fish Hatchery	hatchery brown trout	K/S	negative
10/3/05	Swan River Weir	feral spawning chinook salmon	K/S, OF/milt	negative
10/4/05	Little Manistee River Weir	feral spawning chinook salmon	K/S, OF/milt	negative
10/5/05	Little Manistee River Weir	feral spawning chinook salmon	K/S, OF/milt	negative
	Thompson State Fish Hatchery	hatchery brown trout	K/S	negative
	Iron River, Gibbs City, Iron County	wild brook trout	K/S	negative
	Iron River, Gibbs City, Iron County	wild mottled sculpin	K/S	negative
	Iron River, RV Park, Iron County	wild brook trout	K/S	negative
	Iron River, RV Park, Iron County	wild mottled sculpin	K/S	negative
	Iron River, East Siding, Iron County	wild brook trout	K/S	negative
	Iron River, East Siding, Iron County	wild mottled sculpin	K/S	negative
	Oden State Fish Hatchery	hatchery spawning brown trout	K/S	negative
10/6/05	Little Manistee River Weir	feral spawning chinook salmon	K/S, OF/milt	negative
10/11/05	Lake Macatawa, Ottawa County	wild white bass	K/S	negative
	Lake Macatawa, Ottawa County	wild white perch	K/S	negative
10/12/05	Thompson State Fish Hatchery	hatchery fingerling Atlantic salmon	K/S	negative
	Thompson State Fish Hatchery	hatchery fingerling brown trout	K/S	negative
	Thompson State Fish Hatchery	hatchery propagated fingerling steelhead	K/S	negative
10/14/05	Medusa Creek Weir	feral spawning chinook salmon	K/S, OF/milt	negative
	Boardman River Weir	feral spawning chinook salmon	K/S, OF/milt	negative
10/18/05	Platte River Weir	feral spawning coho salmon	K/S, OF/milt	negative
10/24/05	Oden State Fish Hatchery	hatchery spawning brown trout	K/S	negative
10/25/05	Oden State Fish Hatchery	hatchery spawning brown trout	K/S	negative
10/26/05	Oden State Fish Hatchery	hatchery spawning brown trout	K/S	negative
	Lake Huron, DeTour Village	wild lake whitefish	K/S	negative
	Covert Creek, Van Buren County	wild lamprey	K/S	negative

Supplementary Table S2. *Cont.*

Date	Location	Species & Rearing Condition	Tissue	VHSV Results
10/27/05	Oden State Fish Hatchery	hatchery spawning brown trout	K/S	negative
	Marquette State Fish Hatchery	hatchery spawning brook trout	K/S	negative
10/28/05	Oden State Fish Hatchery	hatchery spawning brown trout	K/S	negative
	Lake Michigan, Naubinway	wild lake whitefish	K/S	negative
10/29/05	Oden State Fish Hatchery	hatchery spawning brown trout	K/S	negative
10/30/05	Oden State Fish Hatchery	hatchery spawning rainbow trout	K/S	negative
10/31/05	Oden State Fish Hatchery	hatchery spawning rainbow trout	K/S	negative
11/1/05	Wolf Lake State Fish Hatchery	hatchery propagated steelhead	K/S	negative
11/2/05	Marquette State Fish Hatchery	hatchery spawning brook trout	K/S	negative
	Lake Macatawa, Ottawa County	wild white bass	K/S	negative
11/3/05	Marquette State Fish Hatchery	hatchery spawning brook trout	K/S	negative
	Marquette State Fish Hatchery	hatchery spawning lake trout	K/S	negative
	Lake Michigan, Big Bay de Noc	wild lake whitefish	K/S	negative
11/9/05	Oden State Fish Hatchery	hatchery spawning brown trout	K/S	negative
	Platte River Weir	feral spawning coho salmon	K/S, OF/milt	negative
	Marquette State Fish Hatchery	hatchery spawning brook trout	K/S	negative
11/18/05	Lake Superior State University	feral spawning Atlantic salmon	K/S, OF/milt	negative
11/23/05	Marquette State Fish Hatchery	hatchery spawning brook trout	OF/milt	negative
11/30/05	Marquette State Fish Hatchery	hatchery spawning brook trout	OF/milt	negative
12/1/05	Aquaculture Facility 33, Chippewa County	aquaculture-raised lake herring	K/S	negative
12/6/05	Marquette State Fish Hatchery	hatchery spawning brook trout	OF/milt	negative
	Marquette State Fish Hatchery	hatchery lake trout	K/S	negative
	Aquaculture Facility 13, Antrim County	aquaculture-raised rainbow trout	K/S	negative
12/7/05	Oden State Fish Hatchery	hatchery fingerling brown trout	K/S	negative
	Oden State Fish Hatchery	hatchery fingerling rainbow trout	K/S	negative

Supplementary Table S3. List of viral hemorrhagic septicemia testing performed in Michigan in 2006 showing site locations, fish species, specimen tested, and test results. K: kidneys, S: spleen, OF: ovarian fluid, SB: swimbladder.

Date	Location	Species & Rearing Condition	Tissue	VHSV Results
1/4/06	Aquaculture Facility 16, Wexford County	aquaculture-raised rainbow trout	K/S	negative
1/11/06	Aquaculture Facility 16, Wexford County	aquaculture-raised rainbow trout	K/S	negative
1/12/06	Lake Huron, DeTour Village	wild lake whitefish	K/S	negative
	Lake Huron, Cheboygan	wild lake whitefish	K/S	negative
1/31/06	Lake Michigan, Big Bay de Noc	wild lake whitefish	K/S	negative
2/1/06	Marquette State Fish Hatchery	hatchery fingerling brook trout	K/S	negative
	Marquette State Fish Hatchery	hatchery fingerling lake trout	K/S	negative
	Marquette State Fish Hatchery	hatchery fingerling splake	K/S	negative
2/2/06	Thompson State Fish Hatchery	hatchery fingerling Atlantic salmon	K/S	negative
	Thompson State Fish Hatchery	hatchery fingerling brown trout	K/S	negative
	Thompson State Fish Hatchery	hatchery propagated fingerling chinook salmon	K/S	negative
	Thompson State Fish Hatchery	hatchery fingerling rainbow trout	K/S	negative
	Thompson State Fish Hatchery	hatchery propagated fingerling steelhead	K/S	negative
	Lake Superior State University	hatchery fingerling Atlantic salmon	K/S	negative
	Oden State Fish Hatchery	hatchery fingerling brown trout	K/S	negative
	Oden State Fish Hatchery	hatchery fingerling rainbow trout	K/S	negative
2/3/06	Harrietta State Fish Hatchery	hatchery fingerling brown trout	K/S	negative
	Harrietta State Fish Hatchery	hatchery fingerling rainbow trout	K/S	negative
	Platte River State Fish Hatchery	hatchery propagated fingerling coho salmon	K/S	negative
	Platte River State Fish Hatchery	hatchery propagated fingerling chinook salmon	K/S	negative
2/10/06	Wolf Lake State Fish Hatchery	hatchery propagated fingerling chinook salmon	K/S	negative
	Wolf Lake State Fish Hatchery	hatchery propagated fingerling steelhead	K/S	negative
2/16/06	Marquette State Fish Hatchery	hatchery lake trout	K/S	negative
2/23/06	Oden State Fish Hatchery	hatchery spawning brown trout	OF	negative
	Oden State Fish Hatchery	hatchery spawning rainbow trout	OF	negative
3/8/06	Harrietta State Fish Hatchery	hatchery brown trout	K/S	negative
3/14/06	Lake St. Clair, Michigan waters	wild gizzard shad	K/S	VHSV positive

Supplementary Table S3. *Cont.*

Date	Location	Species & Rearing Condition	Tissue	VHSV Results
3/20/06	Oden State Fish Hatchery	hatchery rainbow trout	K/S	negative
3/28/06	Lake Michigan, Naubinway	wild lake whitefish	K/S	negative
4/5/06	Wolf Lake State Fish Hatchery	hatchery propagated steelhead	K/S	negative
	Platte River State Fish Hatchery	hatchery propagated chinook salmon	K/S	negative
4/11/06	Hudson Lake, Washtenaw County	wild spawning muskellunge	blood, OF/milt	negative
4/12/06	Thornapple Lake, Barry County	wild spawning muskellunge	blood, OF/milt	negative
4/13/06	Little Manistee River Weir	feral spawning steelhead	K/S, OF/milt	negative
4/26/06	Lake St. Clair	wild muskellunge	K/S	VHSV positive
	Lake St. Clair	wild yellow perch	K/S	VHSV positive
4/27/06	Oden State Fish Hatchery	hatchery brown trout	K/S	negative
5/2/06	Platte River State Fish Hatchery	hatchery propagated chinook salmon	K/S	negative
5/4/06	Thompson State Fish Hatchery	hatchery brown trout	K/S	negative
5/12/06	Golden Lake, Iron County	wild common white sucker	K/S	negative
	Brule Lake, Iron County	wild walleye	K/S	negative
5/16/06	Harrietta State Fish Hatchery	hatchery brown trout	K/S	negative
5/17/06	Lake St. Clair, Michigan waters	wild northern pike	K/S, SB, skin lesions	VHSV positive
	Lake St. Clair, Michigan waters	wild muskellunge	K/S, eyes	VHSV positive
	Lake St. Clair, Michigan waters	wild shorthead redhorse	K/S, skin lesions	VHSV positive
	Lake St. Clair, Michigan waters	wild freshwater drum	K/S, SB, skin lesions	VHSV positive
	Lake St. Clair, Michigan waters	wild rockbass	K/S, skin lesions	VHSV positive
	Lake St. Clair, Michigan waters	wild silver redhorse	K/S, skin lesions	VHSV positive
5/22/06	Lake St. Clair, Michigan waters	wild freshwater drum	K/S, SB, skin lesions, ascitic fluid	VHSV positive
	Lake St. Clair, Michigan waters	wild silver redhorse	K/S	negative
	Lake St. Clair, Michigan waters	wild rockbass	K/S, skin lesions, eyes	VHSV positive
	Lake St. Clair, Michigan waters	wild northern pike	K/S, eyes	VHSV positive
	Lake St. Clair, Michigan waters	wild muskellunge	K/S, SB, skin lesions, liver, eyes	VHSV positive
	Lake St. Clair, Michigan waters	wild freshwater drum	K/S	negative

Supplementary Table S3. *Cont.*

Date	Location	Species & Rearing Condition	Tissue	VHSV Results
5/24/06	Lake St. Clair, Michigan waters	wild freshwater drum	K/S, SB, skin lesions, ascitic fluid	VHSV positive
	Lake St. Clair, Michigan waters	wild muskellunge	K/S, SB, skin lesions, eyes, testes	VHSV positive
5/25/06	Lake Michigan, Big Bay de Noc	wild lake whitefish	K/S	negative
5/31/06	Lake Huron, DeTour Village	wild lake whitefish	K/S	negative
6/2/06	Aquaculture Facility 16, Wexford County	aquaculture-raised rainbow trout	K/S	negative
6/9/06	Camp 8 Lake, Luce County	wild smallmouth bass	K/S	negative
6/13/06	Lake Superior State University	hatchery Atlantic salmon	K/S	negative
6/14/06	Gilchrist Creek, spring-fed, Oscoda County	wild brown trout	K/S	negative
	Gilchrist Creek, spring-fed, Montmorency County	wild brown trout	K/S	negative
	Gilchrist Creek, spring-fed, Montmorency County	wild mottled sculpin	K/S	negative
	Gilchrist Creek, spring-fed, Montmorency County	wild common white sucker	K/S	negative
	Gilchrist Creek, spring-fed, Oscoda County	wild blacknose dace	K/S	negative
6/19/06	Lake Huron, Cheboygan	wild lake whitefish	K/S	negative
6/21/06	Oden State Fish Hatchery	hatchery rainbow trout	K/S	negative
6/28/06	Lake Michigan, Naubinway	wild lake whitefish	K/S	negative
6/29/06	Square Lake Walleye Rearing Pond, Delta County	hatchery walleye	K/S	negative
7/6/06	Wolf Lake State Fish Hatchery	hatchery propagated fingerling steelhead	K/S	negative
7/10/06	Lake St. Clair	wild muskellunge	K/S, SB, skin lesions, musculature, eyes, liver, ovaries, stomach, intestine	VHSV positive
7/11/06	Thousand Island Lake, Gogebic County	wild lake herring	K/S	negative
7/17/06	Wolf Lake State Fish Hatchery	hatchery fingerling lake sturgeon	K/S	negative

Supplementary Table S3. *Cont.*

Date	Location	Species & Rearing Condition	Tissue	VHSV Results
7/18/06	Wolf Lake State Fish Hatchery	hatchery fingerling muskellunge	K/S	negative
7/26/06	Marquette State Fish Hatchery	hatchery fingerling brook trout	K/S	negative
7/27/06	Marquette State Fish Hatchery	hatchery fingerling brook trout	K/S	negative
8/3/06	Harrietta State Fish Hatchery	hatchery brown trout	K/S	negative
	Wolf Lake State Fish Hatchery	hatchery propagated steelhead	K/S	negative
8/14/06	Sturgeon River, Baraga County	wild brown trout	K/S	negative
8/15/06	Lake Huron, DeTour Village	wild lake whitefish	K/S	negative
8/18/06	Lake Michigan, Big Bay de Noc	wild lake whitefish	K/S	negative
	Lake Huron, Cheboygan	wild lake whitefish	K/S, SB	VHSV positive
	Lake Michigan, Naubinway	wild lake whitefish	K/S	negative
8/21/06	Marquette State Fish Hatchery	hatchery brook trout	K/S	negative
	Marquette State Fish Hatchery	hatchery lake trout	K/S	negative
8/22/06	Salmon Trout River, Marquette County	wild brook trout	K/S	negative
8/28/06	Aquaculture Facility 33, Chippewa County	aquaculture-raised walleye	K/S	negative
8/30/06	Harrietta State Fish Hatchery	hatchery brown trout	K/S	negative
9/7/06	Marquette State Fish Hatchery	hatchery brook trout	K/S	negative
9/14/06	Thompson State Fish Hatchery	hatchery brown trout	K/S	negative
9/21/06	Little Manistee River Weir	feral spawning chinook salmon	K/S, OF/milt	negative
9/28/06	Swan River Weir	feral spawning chinook salmon	K/S, OF/milt, SB	VHSV positive
10/2/06	Aquaculture Facility 16, Wexford County	aquaculture-raised rainbow trout	K/S	negative
10/3/06	Oden State Fish Hatchery	hatchery brown trout	K/S	negative
10/6/06	Lake Huron, Thunder Bay	wild lake whitefish	K/S, SB	VHSV positive
	Lake Huron, Thunder Bay	wild walleye	K/S, SB	VHSV positive
10/12/06	Lake Superior State University	hatchery fingerling Atlantic salmon	K/S	negative
10/13/06	Lake Milakokia, Mackinac County	wild northern pike	K/S	negative
10/19/06	Platte River Weir	feral spawning coho salmon	K/S, OF/milt	negative
10/26/06	Platte River Weir	feral spawning coho salmon	K/S, OF/milt	negative

Supplementary Table S3. *Cont.*

Date	Location	Species & Rearing Condition	Tissue	VHSV Results
11/2/06	Oden State Fish Hatchery	hatchery brown trout	K/S	negative
	Oden State Fish Hatchery	hatchery rainbow trout	K/S	negative
11/9/06	Aquaculture Facility 26, Emmet County	aquaculture-raised rainbow trout	K/S	negative
	Aquaculture Facility 30, Ogemaw County	aquaculture-raised rainbow trout	K/S	negative
	Aquaculture Facility 30, Ogemaw County	aquaculture-raised brook trout	K/S	negative
	St. Mary's River, Chippewa County	wild lake herring	K/S	negative
11/16/06	Lake Superior State University	hatchery Atlantic salmon	K/S	negative
	Lake Superior State University	hatchery spawning Atlantic salmon	OF/milt	negative
11/27/06	Aquaculture Facility 1, Clare County	aquaculture-raised brook trout	K/S	negative
	Aquaculture Facility 1, Clare County	aquaculture-raised rainbow trout	K/S	negative
	Aquaculture Facility 1, Clare County	aquaculture-raised brown trout	K/S	negative
11/29/06	Aquaculture Facility 2, Mecosta County	aquaculture-raised rainbow trout	K/S	negative
	Aquaculture Facility 2, Mecosta County	aquaculture-raised brook trout	K/S	negative
	Aquaculture Facility 2, Mecosta County	aquaculture-raised rainbow trout/brown trout	K/S	negative
	Aquaculture Facility 22, Wexford County	aquaculture-raised rainbow trout	K/S	negative
	Aquaculture Facility 22, Wexford County	aquaculture-raised brook trout	K/S	negative
11/30/06	Aquaculture Facility 13, Antrim County	aquaculture-raised rainbow trout	K/S	negative
	Aquaculture Facility 6, Alcona County	aquaculture-raised rainbow trout	K/S	negative
	Aquaculture Facility 6, Alcona County	aquaculture-raised brown trout	K/S	negative
	Aquaculture Facility 6, Alcona County	aquaculture-raised brook trout	K/S	negative
12/4/06	Aquaculture Facility 25, Kalamazoo County	aquaculture-raised koi	K/S	negative
	Aquaculture Facility 25, Kalamazoo County	aquaculture-raised hybrid bluegill	K/S	negative
12/5/06	Buck Lake, Iron County	wild bluegill	K/S	negative
	Buck Lake, Iron County	wild crappie	K/S	negative
12/6/06	Tittabawasee River, Midland County	wild walleye	K/S	negative
12/11/06	Lake Huron, Lexington	wild emerald shiner	K/S	negative
	Lake Huron, Port Sanilac	wild emerald shiner	K/S	negative

Supplementary Table S3. *Cont.*

Date	Location	Species & Rearing Condition	Tissue	VHSV Results
12/12/06	Lake Huron, Port Sanilac	wild spottail shiner	K/S	negative
	Lake Huron, Harbor Beach	wild emerald shiner	K/S	negative
12/13/06	Lake St. Clair, Michigan waters	wild spottail shiner	K/S	VHSV positive
	Lake St. Clair, Michigan waters	wild emerald shiner	K/S	VHSV positive
12/14/06	Lake Huron, Saginaw Bay, Caseville	wild spottail shiner	K/S	negative
	Lake Huron, Saginaw Bay, Caseville	wild emerald shiner	K/S	negative
	Kawkawlin River, Bay County	wild emerald shiner	K/S	negative
	Lake Huron, Saginaw Bay, Tawas	wild emerald shiner	K/S	negative
	Lake Erie, Monroe	wild emerald shiner	K/S	negative
	Detroit River, Wyandotte, Wayne County	wild emerald shiner	K/S	negative
	Lake Huron, Port Huron	wild emerald shiner	K/S	negative
12/19/06	Marquette State Fish Hatchery	hatchery spawning lake trout	OF	negative
	Marquette State Fish Hatchery	hatchery spawning brook trout	OF	negative
12/20/06	Aquaculture Facility 8, Muskegon County	aquaculture-raised brown trout	K/S	negative
	Aquaculture Facility 8, Muskegon County	aquaculture-raised golden rainbow trout	K/S	negative
	Aquaculture Facility 8, Muskegon County	aquaculture-raised rainbow trout	K/S	negative
	Aquaculture Facility 8, Muskegon County	aquaculture-raised brook trout	K/S	negative
	Aquaculture Facility 8, Muskegon County	aquaculture-raised hybrid bluegill	K/S	negative

Supplementary Table S4. List of viral hemorrhagic septicemia testing performed in Michigan in 2007 showing site locations, fish species, specimen tested, and test results. K: kidneys, S: spleen, OF: ovarian fluid, SB: swimbladder.

**Date**	**Location**	**Species & Rearing Condition**	**Tissue**	**VHSV Results**
1/4/07	Platte River State Fish Hatchery	hatchery propagated chinook salmon	K/S	negative
	Thompson State Fish Hatchery	hatchery propagated chinook salmon	K/S	negative
	Wolf Lake State Fish Hatchery	hatchery propagated chinook salmon	K/S	negative
1/9/07	Oden State Fish Hatchery	hatchery spawning brown trout	K/S	negative
	Oden State Fish Hatchery	hatchery spawning brown trout	OF	negative
1/10/07	Muskegon River & Muskegon Lake, Muskegon County	wild walleye	K/S	negative
1/11/07	Platte River State Fish Hatchery	hatchery propagated chinook salmon	K/S	negative
1/16/07	Wolf Lake State Fish Hatchery	hatchery propagated steelhead	K/S	negative
1/22/07	Harrietta State Fish Hatchery	hatchery fingerling rainbow trout	K/S	negative
	Harrietta State Fish Hatchery	hatchery fingerling brown trout	K/S	negative
	Harrietta State Fish Hatchery	hatchery brown trout	K/S	negative
1/25/07	Marquette State Fish Hatchery	hatchery fingerling lake trout	K/S	negative
	Marquette State Fish Hatchery	hatchery fingerling splake	K/S	negative
	Thompson State Fish Hatchery	hatchery fingerling brown trout	K/S	negative
	Thompson State Fish Hatchery	hatchery propagated fingerling steelhead	K/S	negative
1/29/07	Platte River State Fish Hatchery	hatchery propagated fingerling coho salmon	K/S	negative
	Platte River State Fish Hatchery	hatchery propagated fingerling chinook salmon	K/S	negative
	Oden State Fish Hatchery	hatchery fingerling brown trout	K/S	negative
	Oden State Fish Hatchery	hatchery fingerling rainbow trout	K/S	negative
	Oden State Fish Hatchery	hatchery spawning rainbow trout	OF	negative
2/1/07	Lake Michigan, Little Bay de Noc	wild walleye	K/S	negative
	Lake Michigan, Little Bay de Noc	wild northern pike	K/S	negative
	Lake Michigan, Little Bay de Noc	wild yellow perch	K/S	negative
	Lake Michigan, Little Bay de Noc	wild common white sucker	K/S	negative
2/9/07	Oden State Fish Hatchery	hatchery brown trout	K/S	negative

Supplementary Table S4. *Cont.*

Date	Location	Species & Rearing Condition	Tissue	VHSV Results
2/12/07	Marquette State Fish Hatchery	hatchery fingerling brook trout	K/S	negative
	Marquette State Fish Hatchery	hatchery fingerling lake trout	K/S	negative
	Thompson State Fish Hatchery	hatchery fingerling brown trout	K/S	negative
	Thompson State Fish Hatchery	hatchery fingerling rainbow trout	K/S	negative
	Lake Superior State University	hatchery fingerling Atlantic salmon	K/S	negative
2/13/07	Wolf Lake State Fish Hatchery	hatchery propagated fingerling steelhead	K/S	negative
	Oden State Fish Hatchery	hatchery eggs brown trout	eggs	negative
2/16/07	Seven Mile River, Kalamazoo, Calhoun County	wild sculpin spp.	K/S	negative
2/21/07	Thompson State Fish Hatchery	hatchery propagated fingerling chinook salmon	K/S	negative
	Lake Michigan, Little Bay de Noc	wild yellow perch	K/S	negative
	Lake Michigan, Little Bay de Noc	wild walleye	K/S	negative
	Lake Michigan, Little Bay de Noc	wild northern pike	K/S	negative
2/22/07	Muskegon River, Newaygo County	wild walleye	K/S	negative
	Muskegon Lake, Muskegon County	wild yellow perch	K/S	negative
2/23/07	Wolf Lake State Fish Hatchery	hatchery propagated fingerling chinook salmon	K/S	negative
3/6/07	Aquaculture Facility 32, Gogebic County	aquaculture-raised brook trout	K/S	negative
	Aquaculture Facility 32, Gogebic County	aquaculture-raised rainbow trout	K/S	negative
3/14/07	Harrietta State Fish Hatchery	hatchery brown trout	K/S	negative
3/15/07	Lake Huron, Alpena, Thunder Bay River	wild emerald shiner	K/S	negative
3/20/07	Lake Huron, Potagannissing Bay, Drummond Island	wild yellow perch	K/S	negative
3/22/07	Oden State Fish Hatchery	hatchery rainbow trout	K/S	negative
3/27/07	Sanford Lake, Midland County	wild spawning northern pike	K/S, OF/milt	negative
	Lake Huron, Thunder Bay	wild lake whitefish	K/S, skin lesions	negative
3/28/07	Marquette State Fish Hatchery	hatchery fingerling brook trout	K/S	negative
3/30/07	Oden State Fish Hatchery	hatchery brown trout	K/S	negative

Supplementary Table S4. *Cont.*

Date	Location	Species & Rearing Condition	Tissue	VHSV Results
4/3/07	Muskegon River, Newaygo County	wild spawning walleye	K/S, OF/milt	negative
4/4/07	Oden State Fish Hatchery	hatchery brown trout	K/S	negative
4/5/07	Tittabawasee River, Midland County	wild spawning walleye	K/S, OF/milt	negative
4/9/07	Little Manistee River Weir	feral spawning steelhead	K/S, OF/milt	negative
4/12/07	Lake Michigan, Little Bay de Noc	wild spawning northern pike	K/S, OF/milt	negative
4/18/07	Lake Michigan, Little Bay de Noc	wild spawning walleye	K/S, OF/milt	negative
4/23/07	Aquaculture Facility 33, Chippewa County	aquaculture-raised walleye	K/S	negative
5/1/07	Lake Michigan, Grand Haven	wild lake whitefish	K/S	negative
	Lake Michigan, Grand Haven	wild yellow perch	K/S	negative
5/2/07	Budd Lake, Clare County	wild black crappie	K/S, SB, ascitic fluid	VHSV positive
	Budd Lake, Clare County	wild bluegill	K/S	VHSV positive
	Budd Lake, Clare County	wild pumpkinseed	K/S	VHSV positive
	Budd Lake, Clare County	wild largemouth bass	K/S	VHSV positive
	Budd Lake, Clare County	wild golden shiner	K/S	negative
5/4/07	Lake Michigan, Little Bay de Noc	wild walleye	musculature, viscera	negative
5/9/07	Fire Lake, Iron County	wild common white sucker	K/S	negative
5/14/07	Lake St. Clair, Michigan waters	hatchery muskellunge	K/S	negative

Supplementary Table S4. *Cont.*

Date	Location	Species & Rearing Condition	Tissue	VHSV Results
5/15/07	Detroit River, Wayne County	wild spawning walleye	K/S, OF/milt, feces	negative
	Lake Macatawa, Ottawa County	wild gizzard shad	K/S	negative
	Spring Lake, Ottawa County	wild gizzard shad	K/S	negative
	Belleville Lake, Wayne County	wild smallmouth bass	K/S	negative
	Belleville Lake, Wayne County	wild largemouth bass	K/S	negative
	Belleville Lake, Wayne County	wild bluegill	K/S	negative
	Belleville Lake, Wayne County	wild white bass	K/S	negative
	Belleville Lake, Wayne County	wild gizzard shad	K/S	negative
	Lake Michigan, Saugatuck	wild lake whitefish	K/S	negative
	Kent Lake, Livingston County	wild largemouth bass	K/S	negative
	Lake Michigan, Arcadia	wild lake whitefish	K/S	negative
	Lake Michigan, Arcadia	wild yellow perch	K/S	negative
	Lake Michigan, Arcadia	wild alewife	K/S	negative
	Grand River, Kent County	wild walleye	K/S	negative
	Grand River, Kent County	wild shorthead redhorse	K/S	negative
	Grand River, Kent County	wild common white sucker	K/S	negative
	Lake Huron, Thunder Bay	wild lake trout	K/S	negative
	Lake Huron, Thunder Bay	wild lake whitefish	K/S	negative
	Lake Erie, Sterling State Park	wild emerald shiner	K/S	negative
	Lake St. Clair, Michigan waters	wild emerald shiner	K/S	negative
	Lake Michigan, South Haven	wild yellow perch	K/S	negative
	Bishop Lake, Livingston County	wild bluegill	K/S	negative
	Bishop Lake, Livingston County	wild bluntnose minnow	K/S	negative
	Lake Erie, Huron River	wild walleye	K/S	negative
	Hudson Lake, Washtenaw County	wild spawning muskellunge	blood, OF/milt	negative
	Thornapple Lake, Barry County	wild spawning muskellunge	non-lethal samples	negative
	Bishop Lake, Livingston County	wild largemouth bass	K/S	negative

Supplementary Table S4. *Cont.*

Date	Location	Species & Rearing Condition	Tissue	VHSV Results
5/16/07	Lake St. Clair, Michigan waters	wild freshwater drum	K/S	negative
5/18/07	Lake St. Clair, Michigan waters	wild freshwater drum	K/S	negative
5/22/07	Lake St. Clair, Michigan waters	wild freshwater drum	K/S	negative
5/24/07	Lake Michigan, Cedar River	wild lake whitefish	K/S, lesion	negative
5/29/07	Eagle Lake, Allegan County	wild bluegill	K/S	negative
	Eagle Lake, Allegan County	wild black crappie	K/S	negative
	Eagle Lake, Allegan County	wild largemouth bass	K/S	negative
5/30/07	Thompson State Fish Hatchery	hatchery brown trout	K/S	negative
5/31/07	Lake Michigan, Charlevoix	wild lake whitefish	K/S	negative
6/4/07	Aquaculture Facility 33, Chippewa County	aquaculture-raised walleye	K/S	negative
6/6/07	Budd Lake, Clare County	wild bluegill	K/S	negative
	Budd Lake, Clare County	wild largemouth bass	K/S	negative
	Budd Lake, Clare County	wild pumpkinseed	K/S	negative
	Budd Lake, Clare County	wild bluntnose minnow	K/S	negative
	Budd Lake, Clare County	wild sand shiner	K/S	negative
	Budd Lake, Clare County	wild golden shiner	K/S	negative

Supplementary Table S4. *Cont.*

Date	Location	Species & Rearing Condition	Tissue	VHSV Results
6/7/07	Lake St. Clair, Michigan waters	wild spawning muskellunge	blood, OF/milt, feces	negative
	Lake Michigan, Little Bay de Noc	wild yellow perch	K/S	negative
	Lake Michigan, Little Bay de Noc	wild round goby	K/S	negative
	Lake Michigan, Little Bay de Noc	wild spottail shiner	K/S	negative
	Lake Michigan, Grand Haven	wild alewife	K/S	negative
	Pontiac Lake, Oakland County	wild bluegill	K/S	negative
	Pontiac Lake, Oakland County	wild largemouth bass	K/S	negative
	Pontiac Lake, Oakland County	wild bluntnose minnow	K/S	negative
	Lake Huron, Lexington Harbor	wild emerald shiner	K/S	negative
	Lake St. Clair, Michigan waters	wild yellow perch	K/S	negative
	Otsego Lake, Otsego County	wild yellow perch	K/S	negative
	Otsego Lake, Otsego County	wild bluntnose minnow	K/S	negative
	Otsego Lake, Otsego County	wild rockbass	K/S	negative
	Rifle River, Arenac County	wild common white sucker	K/S	negative
	Rifle River, Arenac County	wild brown trout	K/S	negative
	Rifle River, Arenac County	feral steelhead	K/S	negative
	Sanford Lake, Midland County	wild bluegill	K/S	negative
	Sanford Lake, Midland County	wild black crappie	K/S	negative
	Hutchins Lake, Allegan County	wild bluegill	K/S	negative
	Hutchins Lake, Allegan County	wild black crappie	K/S	negative
	Hutchins Lake, Allegan County	wild yellow perch	K/S	negative
	Hutchins Lake, Allegan County	wild bluntnose minnow	K/S	negative
	Twin Lakes, Houghton County	wild bluegill	K/S	negative
	Twin Lakes, Houghton County	wild brown bullhead	K/S	negative
	Twin Lakes, Houghton County	wild rockbass	K/S	negative
	Lake Michigan, Big Bay de Noc	wild round goby	K/S	negative
	Lake Michigan, Big Bay de Noc	wild spottail shiner	K/S	negative
	Lake Michigan, Big Bay de Noc	wild emerald shiner	K/S	negative
	Sturgeon River, Baraga County	wild lake sturgeon	K/S	negative
	Lake Huron, Saginaw Bay	wild lake whitefish	K/S	negative

Supplementary Table S4. *Cont.*

Date	Location	Species & Rearing Condition	Tissue	VHSV Results
6/12/07	Aquaculture Facility 32, Gogebic County	aquaculture-raised rainbow trout	K/S	negative
6/13/07	Oden State Fish Hatchery	hatchery brown trout	K/S	negative
6/14/07	Harrietta State Fish Hatchery	hatchery brown trout	K/S	negative
6/18/07	Eagle Lake, Allegan County	wild bluegill	K/S	negative
	Lake Michigan, Saugatuck	wild round goby	K/S	negative
	Long Lake, Cass County	wild bluegill	K/S	negative
	Long Lake, Cass County	wild largemouth bass	K/S	negative
	Long Lake, Cass County	wild sand shiner	K/S	negative
	Long Lake, Cass County	wild bluntnose minnow	K/S	negative
	Long Lake, Cass County	wild spotfin shiner	K/S	negative
	Lake Michigan, South Haven	feral chinook salmon	K/S	negative
	Silver Lake, Grand Traverse County	wild walleye	K/S	negative
	Silver Lake, Grand Traverse County	wild bluntnose minnow	K/S	negative
	Silver Lake, Grand Traverse County	wild rockbass	K/S	negative
	Lake Superior, Munising	wild lake whitefish	K/S	negative
	Lake Superior, Marquette	wild lake trout	K/S	negative
	Lake Ann, Benzie County	wild smallmouth bass	K/S	negative
	Eagle Lake, Allegan County	wild black crappie	K/S	negative
	Lost Lake, Presque Isle County	wild brown bullhead	K/S	negative
	Lost Lake, Presque Isle County	wild bluntnose minnow	K/S	negative
	Lost Lake, Presque Isle County	wild bluegill	K/S	negative
	Houghton Lake, Roscommon County	wild bluegill	K/S	negative
	Houghton Lake, Roscommon County	wild pumpkinseed	K/S	negative
	Houghton Lake, Roscommon County	wild yellow perch	K/S	negative
	Higgins Lake, Roscommon County	wild brown trout	K/S	negative
	Lake Huron, 40 Mile Point	wild round goby	K/S	negative
	Corey Lake, St. Joseph County	wild bluegill	K/S	negative
	Lake St. Clair, Michigan waters	wild lake sturgeon	blood	negative
	Rainbow Lake, Gratiot County	wild bluegill	K/S	negative
	Long Lake, Cass County	wild warmouth	K/S	negative
	Silver Lake, Grand Traverse County	wild minnow sp.	K/S	negative

Supplementary Table S4. *Cont.*

Date	Location	Species & Rearing Condition	Tissue	VHSV Results
6/22/07	Aquaculture Facility 32, Gogebic County	aquaculture-raised brook trout	K/S	negative
6/26/07	Wolf Lake State Fish Hatchery	hatchery muskellunge	K/S	negative
6/27/07	Black Lake, Kalamazoo County	wild lake sturgeon	K/S	negative
7/12/07	Marquette State Fish Hatchery	hatchery brook trout	K/S	negative
7/17/07	Lake Superior State University	hatchery fingerling Atlantic salmon	K/S	negative
7/18/07	Wolf Lake State Fish Hatchery	hatchery propagated fingerling steelhead	K/S	negative
	Wolf Lake State Fish Hatchery	hatchery fingerling muskellunge	K/S	negative
	Lake Michigan, Ludington to South Haven	feral chinook salmon	K/S	negative
	Thousand Island Lake, Gogebic County	wild lake herring	K/S	negative
	Menominee River, Menominee County	wild freshwater drum	K/S	negative
	Lake Erie, Monroe	wild common carp	K/S	negative
	Lake Erie, Monroe	wild channel catfish	K/S	negative
	Lake Erie, Monroe	wild freshwater drum	K/S	negative
	Lake Huron, Port Huron	wild lake trout	K/S	negative
	Lake Superior, Ontonagon	wild lake whitefish	K/S	negative
	Pere Marquette Lake, Mason County	wild round goby	K/S	negative
	White Lake, Muskegon County	wild walleye	K/S	negative
	White Lake, Muskegon County	wild yellow perch	K/S	negative
	White Lake, Muskegon County	wild round goby	K/S	negative
	White Lake, Muskegon County	wild alewife	K/S	negative
	White Lake, Muskegon County	wild bluegill	K/S	negative
	Lake Michigan, Ludington to South Haven	feral chinook salmon	K/S	negative
	White River, Muskegon County	wild shorthead redhorse	K/S	negative
7/19/07	Harrietta State Fish Hatchery	hatchery brown trout	K/S	negative

Supplementary Table S4. *Cont.*

Date	Location	Species & Rearing Condition	Tissue	VHSV Results
7/25/07	Pere Marquette Lake, Mason County	wild yellow perch	K/S	negative
	Pere Marquette Lake, Mason County	wild rockbass	K/S	negative
	Pere Marquette Lake, Mason County	wild alewife	K/S	negative
	Lake Michigan, Little Bay de Noc	wild yellow perch	K/S	negative
	Lake Michigan, Big Bay de Noc	wild round goby	K/S	negative
	Lake Michigan, Big Bay de Noc	wild walleye	K/S	negative
	Lake Michigan, Little Bay de Noc	wild walleye	K/S	negative
	Lake Michigan, Little Bay de Noc	wild round goby	K/S	negative
	Marquette State Fish Hatchery	hatchery fingerling brook trout	K/S	negative
	Marquette State Fish Hatchery	hatchery brook trout	K/S	negative
	Harrietta State Fish Hatchery	hatchery brown trout	K/S	negative
	Lake Superior, Marquette Harbor	wild lake whitefish	K/S	negative
	Tahquamenon River, Chippewa County	wild yellow perch	K/S	negative
	Tahquamenon River, Chippewa County	wild rockbass	K/S	negative
	Tahquamenon River, Chippewa County	wild common white sucker	K/S	negative
7/31/07	Oden State Fish Hatchery	hatchery fingerling brown trout	K/S	negative
	Lake Superior, Marquette Harbor	wild lake whitefish	K/S	negative
8/2/07	Thompson State Fish Hatchery	hatchery fingerling rainbow trout	K/S	negative
	Thompson State Fish Hatchery	hatchery propagated fingerling steelhead	K/S	negative
8/6/07	Lake Huron, Hammond Bay	wild lake trout	K/S	negative
8/7/07	Platte River State Fish Hatchery	hatchery propagated steelhead	K/S	negative
8/15/07	Marquette State Fish Hatchery	hatchery brook trout	K/S	negative
	Marquette State Fish Hatchery	hatchery lake trout	K/S	negative
8/21/07	Oden State Fish Hatchery	hatchery brown trout	K/S	negative
8/22/07	Lake Michigan, Ludington	feral chinook salmon	K/S	negative
	Lake Michigan, Ludington	feral coho salmon	K/S	negative
	Menominee River, Menominee County	wild rockbass	K/S	negative
	Menominee River, Menominee County	wild yellow perch	K/S	negative

Supplementary Table S4. *Cont.*

Date	Location	Species & Rearing Condition	Tissue	VHSV Results
9/5/07	Oden State Fish Hatchery	hatchery brown trout	K/S	negative
9/11/07	Lake Huron, Saginaw Bay	wild walleye	K/S	negative
	Lake Huron, Saginaw Bay	wild common carp	K/S	negative
	Lake Huron, Saginaw Bay	wild freshwater drum	K/S	negative
	Lake Huron, Saginaw Bay	wild channel catfish	K/S	negative
9/13/07	Aquaculture Facility 33, Chippewa County	aquaculture-raised walleye	K/S	negative
9/14/07	Lake Huron, Saginaw Bay	wild yellow perch	K/S	negative
	Lake Huron, Saginaw Bay	wild round goby	K/S	negative
9/20/07	Marquette State Fish Hatchery	hatchery lake trout	K/S	negative
9/25/07	Harrietta State Fish Hatchery	hatchery brown trout	K/S	negative
9/27/07	Little Manistee River Weir	feral spawning chinook salmon	K/S, OF/milt	negative
10/4/07	Lake Huron, Les Cheneaus Islands	wild yellow perch	K/S	negative
	Lake Huron, Thunder Bay	wild lake trout	K/S	negative
	Lake Huron, Thunder Bay	wild lake whitefish	K/S	negative
10/10/07	Platte River State Fish Hatchery	hatchery propagated steelhead	K/S	negative
10/11/07	Lake Huron, outer Saginaw Bay	wild lake whitefish	K/S	negative
	Lake Huron, outer Saginaw Bay	wild lake trout	K/S	negative
10/15/07	Platte River Weir	feral spawning coho salmon	K/S, OF/milt	negative
10/17/07	Swan River Weir	feral spawning chinook salmon	K/S, OF/milt	negative
10/24/07	Platte River Weir	feral spawning coho salmon	K/S, OF/milt	negative
10/30/07	Marquette State Fish Hatchery	hatchery spawning brook trout	OF/milt	negative
10/31/07	Rifle River, Arenac County	wild brown trout	K/S	negative
	Rifle River, Arenac County	wild rainbow trout	K/S	negative
11/2/07	Oden State Fish Hatchery	hatchery brown trout	K/S	negative
	Oden State Fish Hatchery	hatchery rainbow trout	K/S	negative
11/6/07	Aquaculture Facility 33, Chippewa County	aquaculture-raised walleye	K/S	negative
11/7/07	Oden State Fish Hatchery	hatchery brown trout	K/S	negative
	Oden State Fish Hatchery	hatchery spawning brown trout	OF/milt	negative

Supplementary Table S4. *Cont.*

Date	Location	Species & Rearing Condition	Tissue	VHSV Results
11/8/07	Oden State Fish Hatchery	hatchery brown trout	K/S	negative
11/12/07	Lake Superior State University	hatchery Atlantic salmon	K/S	negative
11/29/07	Bait Collection Facility 14, Alcona County	wild emerald shiner	K/S	negative
	Bait Collection Facility 14, Alcona County	wild spottail shiner	K/S	negative
12/5/07	Bait Collection Facility 21, St. Clair County	wild emerald shiner	K/S	negative
12/12/07	Aquaculture Facility 8, Muskegon County	aquaculture-raised brown trout	K/S	negative
	Aquaculture Facility 8, Muskegon County	aquaculture-raised hybrid bluegill	K/S	negative
	Aquaculture Facility 8, Muskegon County	aquaculture-raised rainbow trout	K/S	negative
	Aquaculture Facility 8, Muskegon County	aquaculture-raised brook trout	K/S	negative
	Marquette State Fish Hatchery	hatchery spawning lake trout	OF	negative
12/27/07	Marquette State Fish Hatchery	hatchery lake trout	K/S	negative

Supplementary Table S5. List of viral hemorrhagic septicemia testing performed in Michigan in 2008 showing site locations, fish species, specimen tested, and test results. K: kidneys, S: spleen, OF: ovarian fluid.

Date	Location	Species & Rearing Condition	Tissue Examined	VHSV Results
1/15/08	Platte River State Fish Hatchery	hatchery propagated coho salmon	K/S	negative
1/23/08	Marquette State Fish Hatchery	hatchery fingerling lake trout	K/S	negative
	Marquette State Fish Hatchery	hatchery fingerling splake	K/S	negative
	Thousand Island Lake, Gogebic County	wild bluegill	K/S	negative
	Thousand Island Lake, Gogebic County	wild pumpkinseed	K/S	negative
	Thousand Island Lake, Gogebic County	wild rockbass	K/S	negative
	Lake Michigan, Pentwater	wild alewife	K/S	negative
	Lake Michigan, Pentwater	wild round goby	K/S	negative
	Lake Michigan, Grand Haven	wild alewife	K/S	negative
	Lake Michigan, Grand Haven	wild round goby	K/S	negative
	Lake Michigan, South Haven	wild round goby	K/S	negative
	Lake Michigan, South Haven	wild alewife	K/S	negative
	St. Joseph River, Calhoun County	wild creek chub	K/S	negative
	St. Joseph River, Calhoun County	wild rainbow darter	K/S	negative
	St. Joseph River, Calhoun County	wild central stoneroller	K/S	negative
	St. Mary's River, Calhoun County	wild lake herring	K/S	negative
1/29/08	Marquette State Fish Hatchery	hatchery fingerling lake trout	K/S	negative
	Marquette State Fish Hatchery	hatchery fingerling brook trout	K/S	negative
	Thompson State Fish Hatchery	hatchery fingerling brown trout	K/S	negative
	Thompson State Fish Hatchery	hatchery propagated fingerling steelhead	K/S	negative
2/4/08	Harrietta State Fish Hatchery	hatchery fingerling rainbow trout	K/S	negative
	Harrietta State Fish Hatchery	hatchery fingerling brown trout	K/S	negative
2/5/08	Oden State Fish Hatchery	hatchery spawning brown trout	OF/milt	negative
	Oden State Fish Hatchery	hatchery spawning rainbow trout	OF/milt	negative
2/6/08	Aquaculture Facility 32, Gogebic County	aquaculture-raised rainbow trout	K/S	negative
	Aquaculture Facility 32, Gogebic County	aquaculture-raised brook trout	K/S	negative
2/7/08	Oden State Fish Hatchery	hatchery fingerling brown trout	K/S	negative
	Oden State Fish Hatchery	hatchery fingerling rainbow trout	K/S	negative
	Platte River State Fish Hatchery	hatchery propagated fingerling coho salmon	K/S	negative
2/11/08	Aquaculture Facility 32, Gogebic County	aquaculture-raised rainbow trout	K/S	negative

Supplementary Table S5. *Cont.*

Date	Location	Species & Rearing Condition	Tissue Examined	VHSV Results
2/13/08	Wolf Lake State Fish Hatchery	hatchery propagated fingerling steelhead	K/S	negative
	Wolf Lake State Fish Hatchery	hatchery propagated fingerling chinook salmon	K/S	negative
2/19/08	Aquaculture Facility 2, Mecosta County	aquaculture-raised rainbow trout	K/S	negative
	Aquaculture Facility 2, Mecosta County	aquaculture-raised brook trout	K/S	negative
	Aquaculture Facility 2, Mecosta County	aquaculture-raised brown trout	K/S	negative
2/20/08	Lake Superior State University	hatchery fingerling Atlantic salmon	K/S	negative
	Platte River State Fish Hatchery	hatchery propagated fingerling chinook salmon	K/S	negative
	Thompson State Fish Hatchery	hatchery propagated fingerling chinook salmon	K/S	negative
	Thompson State Fish Hatchery	hatchery fingerling rainbow trout	K/S	negative
3/4/08	Muskegon River, Newaygo County	wild walleye	K/S	negative
	Lake Michigan, Little Bay de Noc	wild walleye	K/S	negative
	Lake Ovid, Clinton County	wild hybrid bluegill	K/S	negative
	Long Lake, Oakland County	wild bluegill	K/S	negative
	Long Lake, Oakland County	wild yellow perch	K/S	negative
	Kent Lake, Oakland County	wild yellow perch	K/S	negative
	Kent Lake, Oakland County	wild bluegill	K/S	negative
	Crescent Lake, Oakland County	wild bluegill	K/S	negative
	Crescent Lake, Oakland County	wild black crappie	K/S	negative
	Big Lake, Oakland County	wild yellow perch	K/S	negative
	Big Lake, Oakland County	wild bluegill	K/S	negative
	Big Lake, Oakland County	wild black crappie	K/S	negative
	Long Lake, Oakland County	wild pumpkinseed	K/S	negative
	Woodland Lake, Livingston County	wild yellow perch	K/S	negative
	Woodland Lake, Livingston County	wild bluegill	K/S	negative
	Woodland Lake, Livingston County	wild pumpkinseed	K/S	negative
	Crescent Lake, Oakland County	wild yellow perch	K/S	negative
	Big Lake, Oakland County	wild yellow perch	K/S	negative
	Big Lake, Oakland County	wild black crappie	K/S	negative

Supplementary Table S5. *Cont.*

Date	Location	Species & Rearing Condition	Tissue Examined	VHSV Results
3/6/08	Harrietta State Fish Hatchery	hatchery brown trout	K/S	negative
3/10/08	Aquaculture Facility 6, Alcona County	aquaculture-raised rainbow trout	K/S	negative
	Aquaculture Facility 6, Alcona County	aquaculture-raised brook trout	K/S	negative
	Aquaculture Facility 6, Alcona County	aquaculture-raised brown trout	K/S	negative
3/11/08	Aquaculture Facility 13, Antrim County	aquaculture-raised rainbow trout	K/S	negative
3/13/08	East Fish Lake, Montmorency County	wild brook trout	K/S	negative
	Fuller Pond, Montmorency County	wild brook trout	K/S	negative
3/20/08	Harrietta State Fish Hatchery	hatchery brown trout	K/S	negative
3/26/08	Aquaculture Facility 33, Chippewa County	aquaculture-raised walleye	K/S	negative
3/27/08	Belleville Lake, Wayne County	wild gizzard shad	K/S	negative
	Tittabawasee River, Midland County	wild spawning walleye	K/S, OF/milt	negative
4/1/08	Bait Collection Facility 14, Alcona County	wild emerald shiner	K/S	negative
4/2/08	Muskegon River, Newaygo County	wild spawning walleye	K/S, OF/milt	negative
	Bait Collection Facility 19, Arenac County	wild emerald shiner	K/S	negative
4/4/08	Muskegon River, Newaygo County	wild spawning walleye	K/S	negative
4/9/08	Oden State Fish Hatchery	hatchery brown trout	K/S	negative
	Thornapple Lake, Barry County	wild spawning muskellunge	blood	negative
4/10/08	Bait Collection Facility 14, Alcona County	wild emerald shiner	K/S	negative
4/11/08	Muskegon River, Newaygo County	wild spawning walleye	K/S	negative
	Thornapple Lake, Barry County	wild spawning muskellunge	OF/milt, blood	negative
	Hudson Lake, Washtenaw County	wild spawning muskellunge	OF/milt, blood	negative
4/14/08	Little Manistee River Weir	feral spawning steelhead	K/S, OF/milt	negative
4/17/08	Marquette State Fish Hatchery	hatchery lake trout	K/S	negative
4/20/08	Lake Michigan, Little Bay de Noc	wild spawning northern pike	K/S, OF/milt	negative

Supplementary Table S5. *Cont.*

Date	Location	Species & Rearing Condition	Tissue Examined	VHSV Results
4/23/08	Aquaculture Facility 33, Chippewa County	aquaculture-raised walleye	K/S	negative
	Budd Lake, Clare County	wild bluegill	K/S	negative
	Budd Lake, Clare County	wild black crappie	K/S	negative
	Budd Lake, Clare County	wild pumpkinseed	K/S	negative
	Kawkawlin River, Bay County	wild northern pike	K/S	negative
	Kawkawlin River, Bay County	wild brown bullhead	K/S	negative
	Kawkawlin River, Bay County	wild yellow perch	K/S	negative
	Kawkawlin River, Bay County	wild fathead minnow	K/S	negative
	Kawkawlin River, Bay County	wild stickleback	K/S	negative
	Tittabawasee River, Midland County	wild common white sucker	K/S	negative
	Sanford Lake, Midland County	wild spawning northern pike	OF/milt	negative
	Lake Michigan, Little Bay de Noc	wild spawning walleye	K/S	negative
	Kawkawlin River, Bay County	wild various spp.	K/S	negative
4/24/08	Lake Michigan, Little Bay de Noc	wild spawning walleye	K/S, OF/milt	negative
4/28/08	Thornapple Lake, Barry County	wild yellow perch	K/S	negative
	Eagle Lake, Allegan County	wild bluegill	K/S	negative
	Eagle Lake, Allegan County	wild yellow perch	K/S	negative
	Eagle Lake, Allegan County	wild largemouth bass	K/S	negative
	Long Lake, Oakland County	wild yellow perch	K/S	negative
	St. Joseph River, Berrien County	wild walleye	K/S	negative
	St. Joseph River, Berrien County	wild common white sucker	K/S	negative
	St. Joseph River, Berrien County	wild golden redhorse sucker	K/S	negative
	Woodland Lake, Livingston County	wild yellow perch	K/S	negative
	Woodland Lake, Livingston County	wild pumpkinseed	K/S	negative
	Kent Lake, Oakland County	wild pumpkinseed	K/S	negative
	Long Lake, Oakland County	wild yellow perch	K/S	negative
	Lake Michigan, Little Bay de Noc	wild spawning walleye	OF/milt	negative

Supplementary Table S5. *Cont.*

Date	Location	Species & Rearing Condition	Tissue Examined	VHSV Results
5/8/08	Genes Pond, Dickinson County	wild bluegill	K/S	negative
	Genes Pond, Dickinson County	wild black crappie	K/S	negative
	Genes Pond, Dickinson County	wild largemouth bass	K/S	negative
	Lake Superior, Big Bay	wild lake trout	K/S	negative
	Thompson State Fish Hatchery	hatchery walleye	K/S	negative
5/9/08	Oden State Fish Hatchery	hatchery brown trout	K/S	negative
5/15/08	Constantine Ponds, St. Joseph County	wild walleye	whole fry	negative
	Muskegon Pond, Muskegon County	wild walleye	whole fry	negative
	Deep Lake, Barry County	wild bluegill	K/S	negative
	Deep Lake, Barry County	wild black crappie	K/S	negative
	Cedar River Streamside Facility, Menominee County	wild rainbow smelt	K/S	negative
	Lake Huron, Saginaw Bay	wild lake whitefish	K/S	negative
	Lake Superior, Calumet	wild lake trout	K/S	negative
	Lake Michigan, South Haven	wild yellow perch	K/S	negative
	Lake Michigan, Saugatuck	wild yellow perch	K/S	negative
	Lake Michigan, South Haven	wild lake whitefish	K/S	negative
	Lake Michigan, Saugatuck	wild lake whitefish	K/S	negative
	Lake Michigan, South Haven	wild round goby	K/S	negative
	Lake Michigan, Saugatuck	wild round goby	K/S	negative
	Lake Michigan, Grand Haven	wild yellow perch	K/S	negative
	Lake Michigan, Grand Haven	wild lake whitefish	K/S	negative
5/16/08	Bruin Lake, Washtenaw County	wild rockbass	K/S	negative
	Bruin Lake, Washtenaw County	wild bluegill	K/S	negative

Supplementary Table S5. *Cont.*

Date	Location	Species & Rearing Condition	Tissue Examined	VHSV Results
5/20/08	Oden State Fish Hatchery	hatchery rainbow trout	K/S	negative
	Wolf Lake State Fish Hatchery	hatchery muskellunge	K/S	negative
	Hoffman Lake, Charlevoix County	wild yellow perch	K/S	negative
	Hoffman Lake, Charlevoix County	wild rockbass	K/S	negative
	Hoffman Lake, Charlevoix County	wild bluegill	K/S	negative
	Little Whitefish Lake, Montcalm County	wild bluegill	K/S	negative
	Little Whitefish Lake, Montcalm County	wild rockbass	K/S	negative
	Muskallonge Lake, Luce County	wild rockbass	K/S	negative
	Muskallonge Lake, Luce County	wild brown bullhead	K/S	negative
	Pike Lake, Luce County	wild yellow perch	K/S	negative
	Kingston Lake, Alger County	wild yellow perch	K/S	negative
	Kingston Lake, Alger County	wild pumpkinseed	K/S	negative
	Kingston Lake, Alger County	wild largemouth bass	K/S	negative
	Kingston Lake, Alger County	wild smallmouth bass	K/S	negative
5/21/08	Auburn Pond, Bay County	wild walleye	K/S	negative
	Kawkawlin River, Bay County	wild walleye	K/S	negative
5/28/08	Mason County Walleye Pond, Mason County	wild walleye	K/S	negative
5/29/08	Aquaculture Facility 33, Chippewa County	aquaculture-raised walleye	K/S	negative
	Thompson State Fish Hatchery	hatchery brown trout	K/S	negative

Supplementary Table S5. *Cont.*

Date	Location	Species & Rearing Condition	Tissue Examined	VHSV Results
5/30/08	Lake St. Clair, Michigan waters	wild spawning muskellunge	OF/milt	negative
	Sturgeon River, Baraga County	wild spawning lake sturgeon	milt	negative
	Lake Superior, Keweenaw	wild lake trout	K/S	negative
	Lake Superior, Keweenaw	wild lake whitefish	K/S	negative
	Aquaculture Facility 33, Chippewa County	aquaculture-raised fry walleye	whole fry	negative
	Hudson Lake, Washtenaw County	wild spawning muskellunge	OF/milt	negative
	Beatons Lake, Gogebic County	wild bluegill	K/S	negative
	Beatons Lake, Gogebic County	wild common white sucker	K/S	negative
	Beatons Lake, Gogebic County	wild rockbass	K/S	negative
	Fine Lake, Barry County	wild black crappie	K/S	negative
	Fine Lake, Barry County	wild bluegill	K/S	negative
	Little Whitefish Lake, Montcalm County	wild largemouth bass	K/S	negative
6/2/08	Crooked Lake, Clare County	wild yellow perch	K/S	negative
	Crooked Lake, Clare County	wild bluegill	K/S	negative
	Crooked Lake, Clare County	wild rockbass	K/S	negative
	Mott Reservoir, Genesee County	wild walleye	K/S	negative
	Mott Reservoir, Genesee County	wild round goby	K/S	negative
	Mott Reservoir, Genesee County	wild bluegill	K/S	negative
	Holloway Reservoir, Genesee County	wild bluegill	K/S	negative
	Holloway Reservoir, Genesee County	wild yellow perch	K/S	negative
6/3/08	Aquaculture Facility 32, Gogebic County	aquaculture-raised rainbow trout	K/S	negative
	Whitmore Lake, Washtenaw County	wild bluegill	K/S	negative
	Whitmore Lake, Washtenaw County	wild black crappie	K/S	negative
6/5/08	I-75 Rearing Pond, Roscommon County	wild walleye	K/S	negative
6/6/08	Peterson Pond, Menominee County	wild walleye	K/S	negative
	Square Lake Walleye Rearing Pond, Delta County	wild walleye	K/S	negative

Supplementary Table S5. *Cont.*

Date	Location	Species & Rearing Condition	Tissue Examined	VHSV Results
6/9/08	Perch Lake, Clinton County	wild bluegill	K/S	negative
	Perch Lake, Clinton County	wild largemouth bass	K/S	negative
	Perch Lake, Clinton County	wild yellow perch	K/S	negative
	Holloway Reservoir, Genesee County	wild emerald shiner	K/S	negative
6/10/08	Whitmore Lake, Washtenaw County	wild bluntnose minnow	K/S	negative
	Bruin Lake, Washtenaw County	wild bluntnose minnow	K/S	negative
6/11/08	Wolf Lake State Fish Hatchery	hatchery propagated steelhead	K/S	negative
	Square Lake Walleye Rearing Pond, Delta County	wild walleye	K/S	negative
6/17/08	Marquette State Fish Hatchery	hatchery splake	K/S	negative
	Marquette State Fish Hatchery	hatchery lake trout	K/S	negative
6/24/08	Aquaculture Facility 33, Chippewa County	aquaculture-raised walleye	K/S	negative
6/26/08	Big Blue Lake, Muskegon County	wild rockbass	K/S	negative
	Big Blue Lake, Muskegon County	wild bluegill	K/S	negative
	Big Blue Lake, Muskegon County	wild largemouth bass	K/S	negative
	Little Whitefish Lake, Montcalm County	wild largemouth bass	K/S	negative
	Lake Michigan, Little Bay de Noc	wild round goby	K/S	negative
	Lake Michigan, Big Bay de Noc	wild round goby	K/S	negative
	Deep Lake, Barry County	wild yellow perch	K/S	negative
	Lake Michigan, Arcadia	wild alewife	K/S	negative
	Fine Lake, Barry County	wild yellow perch	K/S	negative
	Lake Michigan, Arcadia	wild lake whitefish	K/S	negative
	Lake Superior, Big Bay	wild lake whitefish	K/S	negative
	Lake Huron, Au Sable Point	wild lake whitefish	K/S	negative
	Lake Huron, Au Sable Point	wild lake trout	K/S	negative
	Lake Huron, South Point	wild lake whitefish	K/S	negative
	Lake Huron, Thunder Bay	wild lake trout	K/S	negative
7/14/08	Aquaculture Facility 30, Ogemaw County	aquaculture-raised rainbow trout	K/S	negative
7/18/08	Marquette State Fish Hatchery	hatchery brook trout	K/S	negative

Supplementary Table S5. *Cont.*

Date	Location	Species & Rearing Condition	Tissue Examined	VHSV Results
7/21/08	Pigeon River, Cheboygan County	wild brook stickleback	K/S	negative
	Pigeon River, Cheboygan County	wild fathead minnow	K/S	negative
	Pigeon River, Cheboygan County	wild northern redbelly dace	K/S	negative
	Pigeon River, Cheboygan County	wild brook trout	K/S	negative
	Pigeon River, Cheboygan County	wild western blacknose dace	K/S	negative
	Pigeon River, Cheboygan County	wild creek chub	K/S	negative
	Pigeon River, Cheboygan County	wild common white sucker	K/S	negative
	Pigeon River, Cheboygan County	wild brown trout	K/S	negative
	Pigeon River, Cheboygan County	wild johnny darter	K/S	negative
7/22/08	Platte River State Fish Hatchery	hatchery propagated coho salmon	K/S	negative
7/23/08	Thousand Island Lake, Gogebic County	wild lake herring	K/S	negative
7/30/08	Kawkawlin River, Bay County	wild bluegill	K/S	negative
	Kawkawlin River, Bay County	wild yellow perch	K/S	negative
	Kawkawlin River, Bay County	wild gizzard shad	K/S	negative
7/31/08	Wolf Lake State Fish Hatchery	hatchery propagated fingerling steelhead	K/S	negative
	Wolf Lake State Fish Hatchery	hatchery fingerling muskellunge	K/S	negative
	Muskegon River, Newaygo County	wild rockbass	K/S	negative
	Muskegon River, Newaygo County	wild bluegill	K/S	negative
	Muskegon River, Newaygo County	wild yellow perch	K/S	negative
	Bear Creek, spring-fed, Allegan County	wild common white sucker	K/S	negative
	Bear Creek, spring-fed, Allegan County	wild creek chub	K/S	negative
	Bear Creek, spring-fed, Allegan County	wild bluegill	K/S	negative
	Coldwater River, Branch County	wild bluegill	K/S	negative
	Coldwater River, Branch County	wild rockbass	K/S	negative
	Coldwater River, Branch County	wild horneyhead chub	K/S	negative
	Coldwater River, Branch County	wild common shiner	K/S	negative
	Lake Manuka, Otsego County	wild bluntnose minnow	K/S	negative
	Lake Manuka, Otsego County	wild bluegill	K/S	negative
	Lake Manuka, Otsego County	wild pumpkinseed	K/S	negative

Supplementary Table S5. *Cont.*

Date	Location	Species & Rearing Condition	Tissue Examined	VHSV Results
8/5/08	Marquette State Fish Hatchery	hatchery brook trout	K/S	negative
8/6/08	Marquette State Fish Hatchery	hatchery fingerling brook trout	K/S	negative
	Thompson State Fish Hatchery	hatchery fingerling rainbow trout	K/S	negative
	Thompson State Fish Hatchery	hatchery propagated fingerling steelhead	K/S	negative
	Shiawassee River, Shiawassee County	wild rockbass	K/S	negative
	Shiawassee River, Shiawassee County	wild common white sucker	K/S	negative
	Shiawassee River, Shiawassee County	wild smallmouth bass	K/S	negative
8/7/08	Aquaculture Facility 32, Gogebic County	aquaculture-raised rainbow trout	K/S	negative
	Aquaculture Facility 32, Gogebic County	aquaculture-raised brook trout	K/S	negative
8/12/08	Lake Superior State University	hatchery fingerling Atlantic salmon	K/S	negative
8/27/08	Aquaculture Facility 33, Chippewa County	aquaculture-raised walleye	K/S	negative
8/29/08	Oden State Fish Hatchery	hatchery spawning brown trout	K/S	negative
9/3/08	Looking Glass River, Clinton County	wild bluntnose minnow	K/S	negative
	Looking Glass River, Clinton County	wild pumpkinseed	K/S	negative
	Looking Glass River, Clinton County	wild rockbass	K/S	negative
9/9/08	I-75 Rearing Pond, Roscommon County	wild walleye	K/S	negative
9/16/08	Stanley Creek, spring-fed, Benzie County	wild brown trout	K/S	negative
	Stanley Creek, spring-fed, Benzie County	wild sculpin spp.	K/S	negative
	Brundage Creek, spring-fed, Benzie County	wild brook trout	K/S	negative
	Brundage Creek, spring-fed, Benzie County	wild brown trout	K/S	negative
	Brundage Creek, spring-fed, Benzie County	wild sculpin spp.	K/S	negative
	Brundage Creek, spring-fed, Benzie County	wild rainbow trout	K/S	negative
	Kinney Creek, spring-fed, Benzie County	wild brook trout	K/S	negative
	Kinney Creek, spring-fed, Benzie County	wild sculpin spp.	K/S	negative
9/17/08	Cherry Creek, spring-fed, Marquette County	wild brook trout	K/S	negative
	Cherry Creek, spring-fed, Marquette County	wild brown trout	K/S	negative
	Cherry Creek, spring-fed, Marquette County	wild mottled sculpin	K/S	negative

Supplementary Table S5. *Cont.*

Date	Location	Species & Rearing Condition	Tissue Examined	VHSV Results
9/23/08	Lake Huron, Saginaw Bay	wild channel catfish	K/S	negative
	Lake Huron, Saginaw Bay	wild yellow perch	K/S	negative
	Lake Huron, Saginaw Bay	wild round goby	K/S	negative
9/25/08	Little Manistee River Weir	feral spawning chinook salmon	K/S, OF/milt	negative
10/1/08	Swan River Weir	feral spawning chinook salmon	K/S, OF/milt	negative
10/8/08	Aquaculture Facility 33, Chippewa County	aquaculture-raised walleye	K/S	negative
10/23/08	Platte River Weir	feral spawning coho salmon	K/S, OF/milt	negative
10/28/08	Oden State Fish Hatchery	hatchery brown trout	K/S	negative
	Oden State Fish Hatchery	hatchery rainbow trout	K/S	negative
10/30/08	Platte River State Fish Hatchery	hatchery propagated coho salmon	K/S	negative
11/6/08	Bait Collection Facility 19, Arenac County	wild emerald shiner	K/S	negative
	Bait Collection Facility 19, Arenac County	wild spottail shiner	K/S	negative
11/7/08	Bait Collection Facility 10, Lapeer County	wild emerald shiner	K/S	negative
11/20/08	Lake Superior State University	feral spawning Atlantic salmon	K/S	negative
	St. Mary's River, Chippewa County	wild lake herring	K/S	negative
11/26/08	Bait Collection Facility 14, Alcona County	wild spottail shiner	K/S	negative
	Bait Collection Facility 14, Alcona County	wild emerald shiner	K/S	negative
12/2/08	Bait Collection Facility 21, St. Clair County	wild emerald shiner	K/S	negative
12/9/08	Deadman's Lake, Luce County	wild pumpkinseed	K/S	negative
	Deadman's Lake, Luce County	wild bluegill	K/S	negative
	Pike Lake, Luce County	wild golden shiner	K/S	negative
	Pike Lake, Luce County	wild common shiner	K/S	negative
	Nawakwa Lake, Alger County	wild brown bullhead	K/S	negative
12/10/08	Bait Collection Facility 19, Arenac County	wild emerald shiner	K/S	negative
	Bait Collection Facility 19, Arenac County	wild spottail shiner	K/S	negative

Supplementary Table S6. List of viral hemorrhagic septicemia testing performed in Michigan in 2009 showing site locations, fish species, specimen tested, and test results. K: kidneys, S: spleen, OF: ovarian fluid.

Date	Location	Species & Rearing Condition	Tissue Examined	VHSV Results
1/6/09	Diamond Lake, Newaygo County	wild bluegill	K/S	negative
	Diamond Lake, Newaygo County	wild yellow perch	K/S	negative
	Muskegon Lake, Muskegon County	wild walleye	spleen	negative
1/8/09	Bait Collection Facility 10, Lapeer County	wild emerald shiner	K/S	negative
1/13/09	Marquette State Fish Hatchery	hatchery fingerling lake trout	K/S	negative
	Marquette State Fish Hatchery	hatchery fingerling splake	K/S	negative
	Marquette State Fish Hatchery	hatchery fingerling brook trout	K/S	negative
	Thompson State Fish Hatchery	hatchery fingerling brown trout	K/S	negative
	Thompson State Fish Hatchery	hatchery propagated fingerling steelhead	K/S	negative
	Thompson State Fish Hatchery	hatchery fingerling rainbow trout	K/S	negative
	Marquette State Fish Hatchery	hatchery spawning lake trout	OF/milt	negative
1/14/09	Oden State Fish Hatchery	hatchery spawning brown trout	OF/milt	negative
	Oden State Fish Hatchery	hatchery spawning rainbow trout	OF/milt	negative
2/4/09	Oden State Fish Hatchery	hatchery fingerling rainbow trout	K/S	negative
	Oden State Fish Hatchery	hatchery fingerling brown trout	K/S	negative
	Platte River State Fish Hatchery	hatchery propagated fingerling coho salmon	K/S	negative
	Harrietta State Fish Hatchery	hatchery fingerling brown trout	K/S	negative
	Harrietta State Fish Hatchery	hatchery fingerling rainbow trout	K/S	negative
	Oden State Fish Hatchery	hatchery spawning rainbow trout	OF	negative
	Oden State Fish Hatchery	hatchery spawning brown trout	OF	negative
2/9/09	Aquaculture Facility 8, Muskegon County	aquaculture-raised brook trout	K/S	negative
	Aquaculture Facility 8, Muskegon County	aquaculture-raised hybrid bluegill	K/S	negative
	Aquaculture Facility 8, Muskegon County	aquaculture-raised rainbow trout	K/S	negative
	Aquaculture Facility 8, Muskegon County	aquaculture-raised brown trout	K/S	negative
2/17/09	Wolf Lake State Fish Hatchery	hatchery propagated fingerling steelhead	K/S	negative
2/18/09	Muskegon River, Newaygo County	wild walleye	K/S	negative
2/23/09	Aquaculture Facility 2, Mecosta County	aquaculture-raised rainbow trout	K/S	negative

Supplementary Table S6. *Cont.*

Date	Location	Species & Rearing Condition	Tissue Examined	VHSV Results
2/25/09	Marquette State Fish Hatchery	hatchery fingerling brook trout	K/S	negative
	Marquette State Fish Hatchery	hatchery fingerling lake trout	K/S	negative
	Marquette State Fish Hatchery	hatchery fingerling splake	K/S	negative
2/26/09	Aquaculture Facility 22, Wexford County	aquaculture-raised rainbow trout	K/S	negative
3/3/09	Thompson State Fish Hatchery	hatchery propagated fingerling chinook salmon	K/S	negative
	Wolf Lake State Fish Hatchery	hatchery propagated fingerling chinook salmon	K/S	negative
	Aquaculture Facility 6, Alcona County	aquaculture-raised rainbow trout	K/S	negative
	Aquaculture Facility 6, Alcona County	aquaculture-raised brown trout	K/S	negative
	Aquaculture Facility 6, Alcona County	aquaculture-raised brook trout	K/S	negative
3/4/09	Oden State Fish Hatchery	hatchery fry brown trout	whole fry	negative
	Thompson State Fish Hatchery	hatchery fry brown trout	whole fry	negative
	Harrietta State Fish Hatchery	hatchery fry brown trout	whole fry	negative
	Aquaculture Facility 13, Antrim County	aquaculture-raised rainbow trout	K/S	negative
	Lake Michigan, Little Bay de Noc	wild walleye	K/S	negative
	Harrietta State Fish Hatchery	hatchery fry brown trout & rainbow trout	whole fry	negative
3/10/09	Platte River State Fish Hatchery	hatchery propagated fingerling chinook salmon	K/S	negative
3/12/09	Lake Superior State University	hatchery fingerling Atlantic salmon	K/S	negative
3/19/09	Aquaculture Facility 16, Wexford County	aquaculture-raised rainbow trout	K/S	negative
	Oden State Fish Hatchery	hatchery brown trout	K/S	negative
	Oden State Fish Hatchery	hatchery rainbow trout	K/S	negative
	Oden State Fish Hatchery	hatchery fry brown trout	whole fry	negative
	Oden State Fish Hatchery	hatchery fry rainbow trout	whole fry	negative
3/24/09	Bait Collection Facility 14, Alcona County	aquaculture-raised emerald shiner	K/S	negative
3/25/09	Tittabawasee River, Midland County	wild spawning walleye	K/S, OF/milt	negative
	Sanford Lake, Midland County	wild spawning northern pike	OF/milt	negative
	Oden State Fish Hatchery	hatchery spawning brown trout	OF	negative
3/31/09	Muskegon River, Newaygo County	wild spawning walleye	K/S, OF/milt	negative
4/3/09	Muskegon River, Newaygo County	wild spawning walleye	K/S	negative

Supplementary Table S6. *Cont.*

Date	Location	Species & Rearing Condition	Tissue Examined	VHSV Results
4/6/09	Aquaculture Facility 4, Gladwin County	aquaculture-raised rainbow trout	K/S	negative
	Aquaculture Facility 4, Gladwin County	aquaculture-raised brown trout	K/S	negative
4/8/09	Thornapple Lake, Barry County	wild spawning muskellunge	blood	negative
4/9/09	Bait Collection Facility 10, Lapeer County	wild emerald shiner	K/S	negative
	Muskegon River, Newaygo County	wild spawning walleye	K/S	negative
4/10/09	Aquaculture Facility 17, Alcona County	aquaculture-raised rainbow trout	K/S	negative
4/13/09	Little Manistee River Weir	feral spawning steelhead	K/S, OF/milt	negative
4/15/09	Lake Michigan, Little Bay de Noc	wild spawning northern pike	K/S, OF/milt	negative
	Aquaculture Facility 18, Jackson County	aquaculture-raised rainbow trout	K/S	negative
4/17/09	Aquaculture Facility 33, Chippewa County	aquaculture-raised walleye	K/S	negative
4/22/09	Lake Michigan, Little Bay de Noc	wild spawning walleye	K/S, OF/milt	negative
	Stoney Lake River, Oceana County	wild gizzard shad	K/S	negative
4/24/09	Thompson State Fish Hatchery	fry walleye	whole fry	negative
	Kalamazoo River, Allegan County	wild common white sucker	K/S	negative
	Kalamazoo River, Allegan County	wild longnose sucker	K/S	negative
	Kalamazoo River, Allegan County	wild shorthead redhorse	K/S	negative
	Grand River, Kent County	wild common white sucker	K/S	negative
	Grand River, Kent County	wild longnose sucker	K/S	negative
	Grand River, Kent County	wild shorthead redhorse	K/S	negative
	Lake Michigan, Saugatuck	wild lake whitefish	K/S	negative
	Hudson Lake, Washtenaw County	wild spawning muskellunge	OF/milt	negative
	Thornapple Lake, Barry County	wild spawning muskellunge	OF/milt	negative
4/29/09	Aquaculture Facility 25, Kalamazoo County	aquaculture-raised koi	K/S	negative
	Aquaculture Facility 25, Kalamazoo County	aquaculture-raised hybrid bluegill	K/S	negative
4/30/09	Auburn Pond, Bay County	fry walleye	whole fry	negative
	Muskegon Walleye Pond, Muskegon County	fry walleye	whole fry	negative

Supplementary Table S6. *Cont.*

Date	Location	Species & Rearing Condition	Tissue Examined	VHSV Results
5/4/09	Lake Erie, Monroe	wild white perch	K/S	negative
	Lake Erie, Monroe	wild yellow perch	K/S	negative
	Lake Erie, Monroe	wild channel catfish	K/S	negative
	Lake Erie, Monroe	wild freshwater drum	K/S	negative
5/7/09	Thompson State Fish Hatchery	fry walleye	whole fry	negative
5/11/09	Lake St. Clair, Michigan waters	wild spawning muskellunge	K/S, OF/milt	negative
	Baseline Lake, Livingston County	wild bluegill	K/S	negative
	Baseline Lake, Livingston County	wild rockbass	K/S	negative
	Baseline Lake, Livingston County	wild brown bullhead	K/S	VHSV positive
5/12/09	Beaver Island Walleye Club Pond, Charlevoix County	fry walleye	whole fry	negative
	Lake Superior, Keweenaw Bay	wild lake whitefish	K/S	negative
	Lake Michigan, Cedar River	wild smelt	K/S	negative
5/13/09	Lake St. Clair, Michigan waters	wild spawning muskellunge	K/S, OF/milt	negative
5/14/09	Belmont Walleye Pond, Mecosta County	fry walleye	whole fry	negative
	Clear Lake, St. Joseph County	wild bluegill	K/S	negative
	Clear Lake, St. Joseph County	wild black crappie	K/S	negative
	Clear Lake, St. Joseph County	wild bluntnose minnow	K/S	negative
	Kingston Lake, Alger County	wild yellow perch	K/S	negative
	Kingston Lake, Alger County	wild pumpkinseed	K/S	negative
	Kingston Lake, Alger County	wild largemouth bass	K/S	negative
	Kawkawlin River, Bay County	fry walleye	whole fry	negative
	Lake Huron, Saginaw Bay, Bay Port	wild lake whitefish	K/S	negative
	Lake Huron, Harbor Beach	wild lake trout	K/S	negative
5/18/09	Lake St. Clair, Michigan waters	wild spawning muskellunge	K/S, OF/milt	negative
	Lake St. Clair, Michigan waters	wild spawning muskellunge	OF/milt	VHSV positive
	Lake St. Clair, Michigan waters	wild rockbass	K/S	negative

Supplementary Table S6. *Cont.*

Date	Location	Species & Rearing Condition	Tissue Examined	VHSV Results
5/19/09	Oden State Fish Hatchery	hatchery brown trout	K/S	negative
	Lake Superior, Munising	wild lake trout	K/S	negative
	Lake Superior, Munising	wild lake whitefish	K/S	negative
	Lake Winyah, Alpena County	wild black crappie	K/S	negative
	Lake Winyah, Alpena County	wild yellow bullhead	K/S	negative
	Oden State Fish Hatchery	hatchery spawning brown trout	OF/milt	negative
	Mullett Lake, Cheboygan County	wild rockbass	K/S	negative
5/20/09	Aquaculture Facility 29, Iosco County	aquaculture-raised rainbow trout	K/S	negative
	Aquaculture Facility 29, Iosco County	aquaculture-raised brown trout	K/S	negative
	Wolf Lake State Fish Hatchery	hatchery fingerling lake herring	K/S	negative
	Wolf Lake State Fish Hatchery	hatchery fry muskellunge	whole fry	negative
	Lake Michigan, South Haven	wild yellow perch	K/S	negative
	Lake Michigan, South Haven	wild alewife	K/S	negative
	Lake Superior, Keweenaw Bay	wild lake trout	K/S	negative
	Lake Superior, Keweenaw Bay	wild lake whitefish	K/S	negative
	Lake Superior, Big Bay	wild lake trout	K/S	negative
	Lake Superior, Big Bay	wild lake whitefish	K/S	negative
	Lake Charlevoix, Charlevoix County	wild yellow perch	K/S	negative
5/27/09	Thompson State Fish Hatchery	fry walleye	whole fry	negative
	Lake St. Clair, Michigan waters	wild yellow perch	K/S	negative
	Lake St. Clair, Michigan waters	wild spottail shiner	K/S	negative
	Lake St. Clair, Michigan waters	wild spawning muskellunge	milt	negative
5/28/09	Mason County Walleye Pond, Mason County	fry walleye	whole fry	negative
	Aquaculture Facility 33, Chippewa County	aquaculture-raised fry walleye	whole fry	negative
6/2/09	Lake Michigan, Grand Haven	wild yellow perch	K/S	negative
	Lake Michigan, Grand Haven	wild alewife	K/S	negative
	Aquaculture Facility 30, Ogemaw County	aquaculture-raised rainbow trout	K/S	negative

Supplementary Table S6. *Cont.*

Date	Location	Species & Rearing Condition	Tissue Examined	VHSV Results
6/3/09	Harrietta State Fish Hatchery	hatchery brown trout	K/S	negative
	Harrietta State Fish Hatchery	hatchery rainbow trout	K/S	negative
6/4/09	Dewey Lake, Cass County	wild bluegill	K/S	negative
	Dewey Lake, Cass County	wild largemouth bass	K/S	negative
	Dewey Lake, Cass County	wild bluntnose minnow	K/S	negative
	Lake St. Clair, Michigan waters	wild smallmouth bass	K/S, SB, brain, intestine	VHSV positive
	Lake St. Clair, Michigan waters	wild northern pike	K/S	negative
6/5/09	Rifle Lake, Ogemaw County	wild yellow bullhead	K/S	negative
	Rifle Lake, Ogemaw County	wild rockbass	K/S	negative
	Rifle Lake, Ogemaw County	wild bluntnose minnow	K/S	negative
6/8/09	Duck Lake, Allegan County	wild black crappie	K/S	negative
	Duck Lake, Allegan County	wild bluegill	K/S	negative
	Duck Lake, Allegan County	wild largemouth bass	K/S	negative
	Duck Lake, Allegan County	wild bluntnose minnow	K/S	negative
6/9/09	Square Lake Walleye Rearing Pond, Delta County	fry walleye	whole fry	negative
	Peterson Pond, Menominee County	fry walleye	whole fry	negative
	I-75 Rearing Pond, Roscommon County	fry walleye	whole fry	negative
	Lake Superior, Keweenaw Bay	wild lake whitefish	K/S	negative
	Aquaculture Facility 33, Chippewa County	aquaculture-raised fry walleye	whole fry	negative
6/10/09	Lake Michigan, Arcadia	wild yellow perch	K/S	negative
	Lake Michigan, Arcadia	wild alewife	K/S	negative
	Lake Michigan, Arcadia	wild round goby	K/S	negative
6/12/09	Thompson State Fish Hatchery	hatchery brown trout	K/S	negative

Supplementary Table S6. *Cont.*

Date	Location	Species & Rearing Condition	Tissue Examined	VHSV Results
6/15/09	Lake St. Clair, Michigan waters	wild smallmouth bass	K/S	negative
	Lake St. Clair, Michigan waters	wild rockbass	K/S	negative
	Lake St. Clair, Michigan waters	wild yellow perch	K/S	negative
	Lake St. Clair, Michigan waters	wild bluegill	K/S	negative
	Aquaculture Facility 7, Lake County	aquaculture-raised yellow perch	K/S	negative
	Aquaculture Facility 7, Lake County	aquaculture-raised hybrid bluegill	K/S	negative
	Aquaculture Facility 7, Lake County	aquaculture-raised redear sunfish	K/S	negative
6/16/09	Eel Lake, Gogebic County	wild pumpkinseed	K/S	negative
	Eel Lake, Gogebic County	wild bluegill	K/S	negative
	Eel Lake, Gogebic County	wild yellow perch	K/S	negative
6/18/09	Long Lake, Lapeer County	wild yellow perch	K/S	negative
	Long Lake, Lapeer County	wild bluegill	K/S	negative
	Long Lake, Lapeer County	wild black crappie	K/S	negative
	Monocle Lake, Chippewa County	wild yellow perch	K/S	negative
	Monocle Lake, Chippewa County	wild rockbass	K/S	negative
	Monocle Lake, Chippewa County	wild sand shiner	K/S	negative
	Barron Lake, Cass County	wild bluegill	K/S	negative
	Bear Lake, Manistee County	wild yellow perch	K/S	negative
	Bear Lake, Manistee County	wild sand shiner	K/S	negative
	Bear Lake, Manistee County	wild bluegill	K/S	negative
	Bear Lake, Manistee County	wild rockbass	K/S	negative
6/24/09	Aquaculture Facility 28, Cass County	aquaculture-raised hybrid bluegill	K/S	negative
	Aquaculture Facility 28, Cass County	aquaculture-raised bluegill	K/S	negative
	Aquaculture Facility 28, Cass County	aquaculture-raised white crappie	K/S	negative
	Aquaculture Facility 28, Cass County	aquaculture-raised largemouth bass	K/S	negative
	Aquaculture Facility 28, Cass County	aquaculture-raised redear sunfish	K/S	negative
	Aquaculture Facility 28, Cass County	aquaculture-raised channel catfish	K/S	negative
	Aquaculture Facility 28, Cass County	aquaculture-raised golden shiner	K/S	negative

Supplementary Table S6. *Cont.*

Date	Location	Species & Rearing Condition	Tissue Examined	VHSV Results
6/29/09	Black River, Alcona County	wild lake sturgeon	K/S	negative
7/1/09	Sunday Lake, Gogebic County	wild bluegill	K/S	negative
	Sunday Lake, Gogebic County	wild pumpkinseed	K/S	negative
	Sunday Lake, Gogebic County	wild yellow perch	K/S	negative
	Chippewa Lake, Mecosta County	wild yellow perch	K/S	negative
	Chippewa Lake, Mecosta County	wild bluegill	K/S	negative
	Chippewa Lake, Mecosta County	wild pumpkinseed	K/S	negative
	Lake Huron, Black River	wild lake whitefish	K/S	negative
	Lake Huron, Au Sable Point	wild lake whitefish	K/S	negative
	Lake Huron, Au Sable Point	wild lake trout	K/S	negative
	Lake Huron, Thunder Bay	wild sand shiner	K/S	negative
	Lake Huron, Isaacsons Bay	wild sand shiner	K/S	negative
7/2/09	Payne Lake, Barry County	wild bluegill	K/S	negative
	Payne Lake, Barry County	wild black crappie	K/S	negative
	Payne Lake, Barry County	wild pumpkinseed	K/S	negative
	Fremont Lake, Newaygo County	wild yellow perch	K/S	negative
	Fremont Lake, Newaygo County	wild black crappie	K/S	negative
	Fremont Lake, Newaygo County	wild rockbass	K/S	negative
	Aquaculture Facility 32, Gogebic County	aquaculture-raised rainbow trout	K/S	negative
	Aquaculture Facility 32, Gogebic County	aquaculture-raised brown trout	K/S	negative
	Aquaculture Facility 32, Gogebic County	aquaculture-raised brook trout	K/S	negative
7/7/09	Seven Mile Creek, spring-fed, Spring Creek & Howe Lake, Calhoun County	wild blacknose dace	K/S	negative
	Seven Mile Creek, spring-fed, Spring Creek & Howe Lake, Calhoun County	wild mottled sculpin	K/S	negative
	Seven Mile Creek, spring-fed, Spring Creek & Howe Lake, Calhoun County	wild creek chub	K/S	negative

Supplementary Table S6. *Cont.*

Date	Location	Species & Rearing Condition	Tissue Examined	VHSV Results
7/8/09	Thompson State Fish Hatchery	hatchery fingerling brown trout	K/S	negative
	Oden State Fish Hatchery	hatchery fingerling brown trout	K/S	negative
7/9/09	Aquaculture Facility 16, Wexford County	aquaculture-raised rainbow trout	K/S	negative
	Aquaculture Facility 16, Wexford County	aquaculture-raised hybrid bluegill	K/S	negative
	Aquaculture Facility 16, Wexford County	aquaculture-raised largemouth bass	K/S	negative
	Aquaculture Facility 16, Wexford County	aquaculture-raised yellow perch	K/S	negative
	Aquaculture Facility 16, Wexford County	aquaculture-raised brook trout	K/S	negative
7/14/09	Aquaculture Facility 20, Van Buren County	aquaculture-raised smallmouth bass	whole fry	negative
7/15/09	Thompson State Fish Hatchery	hatchery propagated fingerling steelhead	K/S	negative
	Wolf Lake State Fish Hatchery	hatchery propagated fingerling steelhead	K/S	negative
7/16/09	Marquette State Fish Hatchery	hatchery spawning lake trout	K/S	negative
7/22/09	Platte River State Fish Hatchery	hatchery propagated fingerling coho salmon	K/S	negative
	South Branch White Creek, spring-fed, Tuscola County	wild creek chub	K/S	negative
	South Branch White Creek, spring-fed, Tuscola County	wild rainbow darter	K/S	negative
	South Branch White Creek, spring-fed, Tuscola County	wild common shiner	K/S	negative
7/29/09	Oden State Fish Hatchery	hatchery spawning brown trout	K/S	negative
7/30/09	Wolf Lake State Fish Hatchery	hatchery muskellunge	K/S	negative
8/7/09	Platte River State Fish Hatchery	hatchery propagated fingerling coho salmon	K/S	negative
8/11/09	Lake Superior State University	hatchery fingerling Atlantic salmon	K/S	negative
8/12/09	Wolf Lake State Fish Hatchery	hatchery fingerling muskellunge	K/S	negative
	Mio Impoundment, Oscoda County	wild rockbass	K/S	negative
	Mio Impoundment, Oscoda County	wild common white sucker	K/S	negative
	Mio Impoundment, Oscoda County	wild brown bullhead	K/S	negative
8/18/09	Marquette State Fish Hatchery	hatchery spawning lake trout	K/S	negative
	Marquette State Fish Hatchery	hatchery spawning brook trout	K/S	negative
	Marquette State Fish Hatchery	hatchery fingerling brook trout	K/S	negative
	Marquette State Fish Hatchery	hatchery fingerling lake trout	K/S	negative

Supplementary Table S6. *Cont.*

Date	Location	Species & Rearing Condition	Tissue Examined	VHSV Results
8/19/09	Oden State Fish Hatchery	hatchery spawning brown trout	K/S	negative
	Oden State Fish Hatchery	hatchery fingerling rainbow trout	K/S	negative
	Oden State Fish Hatchery	hatchery brown trout	K/S	negative
8/27/09	Aquaculture Facility 33, Chippewa County	aquaculture-raised walleye	K/S	negative
8/28/09	Thompson State Fish Hatchery	hatchery brown trout	K/S	negative
9/2/09	Lake Michigan, Big Bay de Noc	wild round goby	K/S	negative
	Lake Superior, Black River Harbor/Porcupine Mountains	wild lake trout	K/S	negative
	Lake Superior, Black River Harbor/Porcupine Mountains	wild lake whitefish	K/S	negative
	Lake Superior, Black River Harbor/Porcupine Mountains	wild longnose sucker	K/S	negative
	Lake Superior, Ontonagon	wild lake trout	K/S	negative
	Lake Superior, Ontonagon	wild lake whitefish	K/S	negative
9/10/09	Lake Superior State University	hatchery fingerling Atlantic salmon	K/S	negative
9/15/09	Miners River, Alger County	wild sea lamprey	K/S	negative
	Miners River, Alger County	wild American brook lamprey	K/S	negative
	Munising Falls Creek, Alger County	wild American brook lamprey	K/S	negative
	Seven Mile Creek, spring-fed, Hyde Lake & Seven Mile Lake, Alger County	feral coho salmon	K/S	negative
	Seven Mile Creek, spring-fed, Hyde Lake & Seven Mile Lake, Alger County	wild rainbow trout	K/S	negative
	Lake Michigan, Little Bay de Noc	wild round goby	K/S	negative
	Lake Michigan, Green Bay	wild round goby	K/S	negative
	Silver Creek, Silver Creek Trout Pond, Luce County	wild brook trout	K/S	negative
	Silver Creek, Silver Creek Trout Pond, Luce County	wild mottled sculpin	K/S	negative
	Fox River, Schoolcraft County	wild brook trout	K/S	negative
	Fox River, Schoolcraft County	wild mottled sculpin	K/S	negative
	Lake Michigan, Big Bay de Noc	wild common white sucker	K/S	negative

Supplementary Table S6. *Cont.*

Date	Location	Species & Rearing Condition	Tissue Examined	VHSV Results
9/17/09	Oden State Fish Hatchery	hatchery brown trout	K/S	negative
	Lake Huron, Saginaw Bay	wild yellow perch	K/S	negative
	Lake Huron, Saginaw Bay	wild freshwater drum	K/S	negative
	Lake Huron, Saginaw Bay	wild channel catfish	K/S	negative
	Lake Huron, Saginaw Bay	wild common carp	K/S	negative
	Lake Huron, Saginaw Bay	wild common white sucker	K/S	negative
9/18/09	Aquaculture Facility 31, Newaygo County	aquaculture-raised rainbow trout	K/S	negative
	Aquaculture Facility 31, Newaygo County	aquaculture-raised bluegill	K/S	negative
	Aquaculture Facility 31, Newaygo County	aquaculture-raised largemouth bass	K/S	negative
9/29/09	Little Manistee River Weir	feral spawning chinook salmon	K/S, OF/milt	negative
10/6/09	Swan River Weir	feral spawning chinook salmon	K/S, OF/milt	negative
10/19/09	Platte River Weir	feral spawning coho salmon	K/S, OF/milt	negative
	Aquaculture Facility 32, Gogebic County	aquaculture-raised rainbow trout	K/S	negative
10/29/09	Slagle Creek, spring-fed, Manistee County	wild brown trout	K/S	negative
	Slagle Creek, spring-fed, Manistee County	wild brook trout	K/S	negative
11/6/09	Oden State Fish Hatchery	hatchery spawning brown trout	K/S	negative
	Oden State Fish Hatchery	hatchery spawning rainbow trout	K/S	negative
11/10/09	Lake Superior State University	feral spawning Atlantic salmon	K/S	negative
11/11/09	Bait Collection Facility 19, Arenac County	wild emerald shiner	K/S	negative
	Bait Collection Facility 19, Arenac County	wild spottail shiner	K/S	negative
	Bait Collection Facility 14, Alcona County	wild emerald shiner	K/S	negative
11/13/09	Bait Collection Facility 10, Lapeer County	wild emerald shiner	K/S	negative
11/24/09	Oden State Fish Hatchery	hatchery spawning brown trout	OF/milt	negative
12/2/09	Bait Collection Facility 21, St. Clair County	wild emerald shiner	K/S	negative
12/4/09	Bait Collection Facility 24, Tuscola County	wild emerald shiner	K/S	negative
12/9/09	Bait Collection Facility 14, Alcona County	wild spottail shiner	K/S	negative
12/10/09	Oden State Fish Hatchery	hatchery brown trout	K/S	negative

Supplementary Table S6. *Cont.*

Date	Location	Species & Rearing Condition	Tissue Examined	VHSV Results
12/16/09	Bait Collection Facility 19, Arenac County	wild emerald shiner	K/S	negative
	Bait Collection Facility 19, Arenac County	wild spottail shiner	K/S	VHSV positive
12/18/09	Bait Collection Facility 10, Lapeer County	wild emerald shiner	K/S	negative
12/22/09	Oden State Fish Hatchery	hatchery brown trout	K/S	negative

Supplementary Table S7. List of viral hemorrhagic septicemia testing performed in Michigan in 2010 showing site locations, fish species, specimen tested, and test results. K: kidneys, S: spleen, H: heart, OF: ovarian fluid.

Date	Location	Species & Rearing Condition	Tissue Examined	VHSV Results
1/20/10	Aquaculture Facility 8, Muskegon County	aquaculture-raised rainbow trout	K/S/H	negative
	Aquaculture Facility 8, Muskegon County	aquaculture-raised brown trout	K/S/H	negative
	Aquaculture Facility 8, Muskegon County	aquaculture-raised brook trout	K/S/H	negative
	Aquaculture Facility 8, Muskegon County	aquaculture-raised hybrid bluegill	K/S/H	negative
1/28/10	Wolf Lake State Fish Hatchery	hatchery propagated fingerling steelhead	K/S	negative
	Oden State Fish Hatchery	hatchery spawning brown trout	OF/milt	negative
	Oden State Fish Hatchery	hatchery spawning rainbow trout	OF/milt	negative
	Aquaculture Facility 32, Gogebic County	aquaculture-raised rainbow trout	K/S	negative
	Aquaculture Facility 32, Gogebic County	aquaculture-raised brown trout	K/S	negative
	Aquaculture Facility 32, Gogebic County	aquaculture-raised brook trout	K/S	negative
2/2/10	Marquette State Fish Hatchery	hatchery fingerling lake trout	K/S	negative
	Marquette State Fish Hatchery	hatchery fingerling splake	K/S	negative
	Thompson State Fish Hatchery	hatchery fingerling brown trout	K/S	negative
	Thompson State Fish Hatchery	hatchery propagated fingerling steelhead	K/S	negative
2/4/10	Harrietta State Fish Hatchery	hatchery fingerling brown trout	K/S	negative
2/16/10	Oden State Fish Hatchery	hatchery fingerling brown trout	K/S	negative
	Platte River State Fish Hatchery	hatchery propagated fingerling coho salmon	K/S	negative
	Harrietta State Fish Hatchery	hatchery fingerling brown trout	K/S	negative
	Harrietta State Fish Hatchery	hatchery fingerling rainbow trout	K/S	negative
2/23/10	Muskegon River, Newaygo County	wild walleye	K/S	negative
3/2/10	Wolf Lake State Fish Hatchery	hatchery propagated fingerling chinook salmon	K/S	negative
	Marquette State Fish Hatchery	hatchery fingerling brook trout	K/S	negative
	Oden State Fish Hatchery	hatchery fingerling rainbow trout	K/S	negative
	Lake Michigan, Little Bay de Noc	wild walleye	K/S	negative
	Aquaculture Facility 13, Antrim County	aquaculture-raised rainbow trout	K/S/H	negative

Supplementary Table S7. *Cont.*

Date	Location	Species & Rearing Condition	Tissue Examined	VHSV Results
3/9/10	Platte River State Fish Hatchery	hatchery fingerling Atlantic salmon	K/S	negative
	Platte River State Fish Hatchery	hatchery propagated fingerling chinook salmon	K/S	negative
	Thompson State Fish Hatchery	hatchery propagated fingerling chinook salmon	K/S	negative
	Marquette State Fish Hatchery	hatchery fingerling brook trout	K/S	negative
3/10/10	Aquaculture Facility 6, Alcona County	aquaculture-raised rainbow trout	K/S/H	negative
	Aquaculture Facility 6, Alcona County	aquaculture-raised brown trout	K/S/H	negative
	Aquaculture Facility 6, Alcona County	aquaculture-raised brook trout	K/S/H	negative
3/11/10	Aquaculture Facility 16, Wexford County	aquaculture-raised rainbow trout	K/S/H	negative
3/23/10	Sanford Lake, County	wild spawning northern pike	OF/milt	negative
	Tittabawasee River, Midland County	wild spawning walleye	K/S, OF/milt	negative
3/24/10	Aquaculture Facility 8, Muskegon County	aquaculture-raised golden rainbow trout	K/S/H	negative
	Aquaculture Facility 8, Muskegon County	aquaculture-raised brook trout	K/S/H	negative
	Aquaculture Facility 8, Muskegon County	aquaculture-raised rainbow trout	K/S/H	negative
	Aquaculture Facility 9, Newaygo County	aquaculture-raised brook trout	K/S/H	negative
	Aquaculture Facility 9, Newaygo County	aquaculture-raised rainbow trout	K/S/H	negative
3/26/10	Bait Collection Facility 14, Alcona County	wild emerald shiner	K/S/H	negative
	Muskegon River, Newaygo County	wild spawning walleye	K/S	negative
3/30/10	Muskegon River, Newaygo County	wild spawning walleye	K/S, OF/milt	negative
3/31/10	Kent Lake, Oakland County	wild spawning northern pike	blood, OF/milt	negative
	Kent Lake, Oakland County	wild bluegill	K/S/H	negative
	Kent Lake, Oakland County	wild black crappie	K/S/H	negative
	Huron River, Wayne County	wild largemouth bass	K/S/H	negative
	Huron River, Wayne County	wild pumpkinseed	K/S/H	negative
	Huron River, Wayne County	wild common white sucker	K/S/H	negative
	Thornapple Lake, Barry County	wild spawning muskellunge	blood, musculature	negative
4/2/10	Lake Michigan, Little Bay de Noc	wild spawning walleye	K/S, OF/milt	negative
4/6/10	Lake Michigan, Little Bay de Noc	wild spawning walleye	K/S	negative
	Aquaculture Facility 33, Chippewa County	aquaculture-raised walleye	K/S/H	negative

Supplementary Table S7. *Cont.*

Date	Location	Species & Rearing Condition	Tissue Examined	VHSV Results
4/7/10	Lake Superior State University	hatchery fingerling Atlantic salmon	K/S	negative
	Hudson Lake, Washtenaw County	wild spawning muskellunge	blood, OF/milt	negative
4/13/10	Little Manistee River Weir	feral spawning steelhead	K/S/H, OF/milt	negative
	Lake Superior, Whitefish Bay, Taquamenon Bay	wild rainbow smelt	K/S/H	negative
4/14/10	Platte River State Fish Hatchery	hatchery Atlantic salmon	K/S/H	negative
4/19/10	Big Portage Lake, Washtenaw County	wild brown bullhead	K/S/H	negative
	Big Portage Lake, Washtenaw County	wild bluegill	K/S/H	negative
	Big Portage Lake, Washtenaw County	wild rockbass	K/S/H	negative
	Belleville Lake, Wayne County	wild largemouth bass	K/S/H	negative
	Belleville Lake, Wayne County	wild bluegill	K/S/H	negative
	Belleville Lake, Wayne County	wild yellow perch	K/S/H	negative
	Baseline Lake, Livingston County	wild brown bullhead	K/S/H	negative
	Baseline Lake, Livingston County	wild bluegill	K/S/H	negative
	Baseline Lake, Livingston County	wild rockbass	K/S/H	negative
	Stoney Creek Impoundment, Macomb County	wild largemouth bass	K/S/H	negative
	Stoney Creek Impoundment, Macomb County	wild bluegill	K/S/H	negative
	Stoney Creek Impoundment, Macomb County	wild yellow perch	K/S/H	negative
4/26/10	Kent Lake, Oakland County	wild largemouth bass	K/S/H	negative
	Whitmore Lake, Washtenaw County	wild redear sunfish	K/S/H	negative
	Whitmore Lake, Washtenaw County	wild bluegill	K/S/H	negative
	Whitmore Lake, Washtenaw County	wild bluntnose minnow	K/S/H	negative
4/28/10	Wolf Lake State Fish Hatchery	hatchery fingerling muskellunge	K/S	negative
	Lake Michigan, Little Bay de Noc	wild spawning northern pike	K/S/H, OF/milt	negative
	Thompson State Fish Hatchery	fry walleye	whole fry	negative
	Kalamazoo River, Allegan County	wild common white sucker	K/S	negative
	Lake Erie, Bolles Harbor	wild freshwater drum	K/S	negative
	Thornapple Lake, Barry County	wild spawning muskellunge	OF/milt	negative
	Lake Erie, Bolles Harbor	wild white perch	K/S/H	negative
	Lake Erie, Bolles Harbor	wild yellow perch	K/S/H	negative

Supplementary Table S7. *Cont.*

Date	Location	Species & Rearing Condition	Tissue Examined	VHSV Results
4/29/10	Belmont Walleye Pond, Mecosta County	fry walleye	whole fry	negative
	Muskegon Pond, Muskegon County	fry walleye	whole fry	negative
	Sanford Lake, Midland County	wild common carp	K/S/H	negative
5/4/10	Budd Lake, Clare County	wild yellow perch	K/S/H	negative
	Budd Lake, Clare County	wild bluegill	K/S/H	negative
	Budd Lake, Clare County	wild pumpkinseed	K/S/H	negative
	Barton Pond, Washtenaw County	wild bluegill	K/S/H	negative
	Barton Pond, Washtenaw County	wild bluntnose minnow	K/S/H	negative
	Barton Pond, Washtenaw County	wild largemouth bass	K/S/H	negative
	Kawkawlin River, Bay County	fry walleye	whole fry	negative
	Auburn Pond, Bay County	fry walleye	whole fry	negative
5/5/10	Lake St. Clair, Michigan waters	wild muskellunge	blood	negative
	Lake Superior, Whitefish Point	wild lake whitefish	K/S	negative
5/7/10	Tom's Lake, Schoolcraft County	wild bluegill	K/S/H	negative
	Sturgeon River, Baraga County	wild spawning lake sturgeon	OF/milt	negative
	Lake St. Clair, Michigan waters	wild spawning muskellunge	blood, OF/milt	negative
	Lake St. Clair, Michigan waters	wild common carp	blood	negative
	Lake St. Clair, Michigan waters	wild channel catfish	blood	negative
	Lake St. Clair, Michigan waters	wild northern pike	blood	negative
	Lake St. Clair, Michigan waters	wild shorthead redhorse	blood	negative
	Lake St. Clair, Michigan waters	wild pumpkinseed	blood	negative
	Lake St. Clair, Michigan waters	wild rockbass	blood	negative
	Lake St. Clair, Michigan waters	wild smallmouth bass	blood	negative
	Lake St. Clair, Michigan waters	wild silver redhorse	blood	negative
	Lake St. Clair, Michigan waters	wild white perch	blood	negative
	Lake St. Clair, Michigan waters	wild rockbass	K/S/H	negative
	Lake St. Clair, Michigan waters	wild smallmouth bass	K/S/H	negative

Supplementary Table S7. *Cont.*

Date	Location	Species & Rearing Condition	Tissue Examined	VHSV Results
5/10/10	Lake St. Clair, Michigan waters	wild spawning muskellunge	K/S, OF/milt, blood	negative
	Lake St. Clair, Michigan waters	wild common carp	blood	negative
	Lake St. Clair, Michigan waters	wild channel catfish	blood	negative
	Lake St. Clair, Michigan waters	wild common white sucker	blood	negative
	Lake St. Clair, Michigan waters	wild freshwater drum	blood	negative
	Lake St. Clair, Michigan waters	wild longnose gar	blood	negative
	Lake St. Clair, Michigan waters	wild northern pike	blood	negative
	Lake St. Clair, Michigan waters	wild shorthead redhorse	blood	negative
	Lake St. Clair, Michigan waters	wild pumpkinseed	blood	negative
	Lake St. Clair, Michigan waters	wild quillback	blood	negative
	Lake St. Clair, Michigan waters	wild rockbass	blood	negative
	Lake St. Clair, Michigan waters	wild smallmouth bass	blood	negative
	Lake St. Clair, Michigan waters	wild silver redhorse	blood	negative
	Lake St. Clair, Michigan waters	wild walleye	blood	negative
	Lake St. Clair, Michigan waters	wild white bass	blood	negative
	Lake St. Clair, Michigan waters	wild white perch	blood	negative
	Lake St. Clair, Michigan waters	wild yellow perch	blood	negative
5/11/10	Aquaculture Facility 33, Chippewa County	aquaculture-raised fry walleye	whole fry	negative
	Thompson State Fish Hatchery	fry walleye	whole fry	negative
5/12/10	I-75 Rearing Pond, Roscommon County	fry walleye	whole fry	negative
	Mason County Walleye Pond, Mason County	fry walleye	whole fry	negative
	Lake Superior, Keweenaw Bay	wild lake whitefish	K/S	negative
	Lake Superior, Keweenaw Bay	wild lake trout	K/S	negative
	Independence Lake, Washtenaw County	wild black crappie	K/S/H	negative
	Independence Lake, Washtenaw County	wild bluegill	K/S/H	negative
	Independence Lake, Washtenaw County	wild redear sunfish	K/S/H	negative
	Lake St. Clair, Michigan waters	wild spawning muskellunge	K/S, blood, OF/milt	negative

Supplementary Table S7. *Cont.*

Date	Location	Species & Rearing Condition	Tissue Examined	VHSV Results
5/18/10	Beaver Island Walleye Club Pond, Charlevoix County	fry walleye	whole fry	negative
	Lake St. Clair, Michigan waters	wild spawning muskellunge	blood, OF/milt	negative
	Lake Superior, Whitefish Bay	wild lake trout	K/S	negative
5/19/10	Lake St. Clair, Michigan waters	wild spawning muskellunge	blood, OF/milt	negative
5/20/10	Lake St. Clair, Michigan waters	wild emerald shiner	K/S/H	negative
	Peterson Pond, Menominee County	fry walleye	whole fry	negative
	Square Lake Walleye Rearing Pond, Delta County	fry walleye	whole fry	negative
5/21/10	Loon Lake, Oakland County	wild brown bullhead	K/S/H	negative
	Loon Lake, Oakland County	wild bluegill	K/S/H	negative
	Loon Lake, Oakland County	wild largemouth bass	K/S/H	negative
	Lake Michigan, Grand Haven	wild yellow perch	K/S/H	negative
	Lake Michigan, Grand Haven	wild alewife	K/S/H	negative
	Lake Michigan, Grand Haven	wild round goby	K/S/H	negative
	Lake Michigan, Grand Haven	wild alewife	K/S/H	negative
	Lake Michigan, South Haven	wild round goby	K/S/H	negative
	Thompson State Fish Hatchery	fry walleye	whole fry	negative
	Lake Michigan, Saugatuck	wild lake whitefish	K/S	negative
	Lake Michigan, Saugatuck	wild alewife	K/S/H	negative
	Lake Michigan, Saugatuck	wild yellow perch	K/S/H	negative
	Pere Marquette Lake, Mason County	wild brown trout	K/S/H	negative
5/27/10	Otter Lake, Houghton County	wild yellow perch	K/S/H	negative
	Otter Lake, Houghton County	wild black crappie	K/S/H	negative
	Otter Lake, Houghton County	wild brown bullhead	K/S/H	negative
	Lake Superior, Munising	wild lake trout	K/S	negative
	Lake Superior, Munising	wild lake whitefish	K/S	negative
5/28/10	Hoistington Lake, Livingston County	wild bluegill	K/S/H	negative
	Hoistington Lake, Livingston County	wild rockbass	K/S/H	negative
	Hoistington Lake, Livingston County	wild bluntnose minnow	K/S/H	negative

Supplementary Table S7. *Cont.*

Date	Location	Species & Rearing Condition	Tissue Examined	VHSV Results
6/1/10	Lakeville Lake, Oakland County	wild bluegill	K/S/H	negative
	Lakeville Lake, Oakland County	wild rockbass	K/S/H	negative
	Lakeville Lake, Oakland County	wild largemouth bass	K/S/H	negative
6/3/10	Platte River State Fish Hatchery	hatchery Atlantic salmon	K/S/H	negative
	Aquaculture Facility 33, Chippewa County	aquaculture-raised walleye	K/S/H	negative
6/4/10	Wolf Lake State Fish Hatchery	hatchery muskellunge	K/S/H	negative
	St. Clair River	wild lake sturgeon	blood	negative
6/10/10	Lake Michigan, Ludington	wild lake whitefish	K/S	negative
	Lake Michigan, Arcadia	wild alewife	K/S/H	negative
6/14/10	Menominee River, Menominee County	wild brown bullhead	K/S/H	negative
6/15/10	Hamlin Lake, Mason County	wild bluegill	K/S/H	negative
	Hamlin Lake, Mason County	wild rockbass	K/S	negative
	Hamlin Lake, Mason County	wild pumpkinseed	K/S	negative
	Hamlin Lake, Mason County	wild black crappie	K/S/H	negative
6/16/10	Lake Huron, Saginaw Bay, Bay Port	wild lake whitefish	K/S	negative
	Lake Huron, Black River	wild lake trout	K/S	negative
	Prickett Lake, Houghton County	wild smallmouth bass	whole fry	negative
	Prickett Lake, Houghton County	wild bluegill	K/S/H	negative
	Prickett Lake, Houghton County	wild black crappie	K/S/H	negative
	Lake Huron, Black River	wild lake whitefish	K/S	negative
6/17/10	Lake Michigan, Ludington	wild lake whitefish	K/S	negative
6/18/10	Shadow Lake, Gogebic County	wild bluegill	K/S/H	negative
6/22/10	Wolf Lake State Fish Hatchery	hatchery propagated steelhead	K/S/H	negative
6/24/10	Clinton River, Macomb County	wild common white sucker	K/S/H	negative
	Clinton River, Macomb County	wild rockbass	K/S/H	negative
	Cherry Creek, spring-fed, Marquette County	wild brook trout	K/S/H	negative
	Cherry Creek, spring-fed, Marquette County	wild brown trout	K/S/H	negative
	Cherry Creek, spring-fed, Marquette County	wild mottled sculpin	K/S/H	negative

Supplementary Table S7. *Cont.*

Date	Location	Species & Rearing Condition	Tissue Examined	VHSV Results
7/1/10	Prairie River Lake, St. Joseph County	wild black crappie	K/S/H	negative
	Prairie River Lake, St. Joseph County	wild bluegill	K/S/H	negative
	Prairie River Lake, St. Joseph County	wild yellow perch	K/S/H	negative
	Wababis Lake, Kent County	wild yellow perch	K/S/H	negative
	Wababis Lake, Kent County	wild pumpkinseed	K/S/H	negative
	Wababis Lake, Kent County	wild bluegill	K/S/H	negative
	Lake St. Clair, Michigan waters	wild yellow perch	K/S/H	negative
	Van Etten Lake, Iosco County	wild yellow perch	K/S/H	negative
	Van Etten Lake, Iosco County	wild rockbass	K/S/H	negative
	McCollum Lake, Oscoda County	wild pumpkinseed	K/S/H	negative
	McCollum Lake, Oscoda County	wild bluegill	K/S/H	negative
	Lake St. Helen, Roscommon County	wild rockbass	K/S/H	negative
	Lake St. Helen, Roscommon County	wild black crappie	K/S/H	negative
	Lake St. Helen, Roscommon County	wild bluegill	K/S/H	negative
	Avalon Lake, Montmorency County	wild rockbass	K/S/H	negative
	Avalon Lake, Montmorency County	wild yellow perch	K/S/H	negative
7/8/10	Lake Superior State University	hatchery fingerling Atlantic salmon	K/S	negative
7/20/10	Thompson State Fish Hatchery	hatchery propagated fingerling steelhead	K/S	negative
	Wolf Lake State Fish Hatchery	hatchery propagated fingerling steelhead	K/S	negative
7/21/10	Platte River State Fish Hatchery	hatchery propagated fingerling coho salmon	K/S	negative
	Oden State Fish Hatchery	hatchery fingerling brown trout	K/S	negative
7/22/10	Lake Superior, Ontonagon	wild lake sturgeon	K/S/H	negative
	Tahquamenon River, Chippewa County	wild yellow perch	K/S/H	negative
7/27/10	Brundage Creek, spring-fed, Benzie County	wild brook trout & brown trout	K/S/H	negative
7/28/10	Aquaculture Facility 6, Alcona County	aquaculture-raised brown trout	K/S/H	negative
	Stanley Creek, spring-fed, Benzie County	wild brook trout & brown trout	K/S/H	negative

Supplementary Table S7. *Cont.*

Date	Location	Species & Rearing Condition	Tissue Examined	VHSV Results
7/29/10	Aquaculture Facility 32, Gogebic County	aquaculture-raised rainbow trout	K/S/H	negative
	Aquaculture Facility 32, Gogebic County	aquaculture-raised brook trout	K/S/H	negative
	Aquaculture Facility 32, Gogebic County	aquaculture-raised brown trout	K/S/H	negative
7/30/10	Harrietta State Fish Hatchery	hatchery brown trout	K/S/H	negative
8/2/10	Kalamazoo River, Calhoun County	wild common white sucker	K/S/H	negative
	Kalamazoo River, Calhoun County	wild common shiner	K/S/H	negative
	Kalamazoo River, Calhoun County	wild golden redhorse sucker	K/S/H	negative
8/10/10	Wolf Lake State Fish Hatchery	hatchery propagated steelhead	K/S/H	negative
8/11/10	Oden State Fish Hatchery	hatchery brown trout	K/S/H	negative
8/12/10	Kalamazoo River, Kalamazoo County	wild golden redhorse sucker	K/S/H	negative
	Kalamazoo River, Kalamazoo County	wild spotfin shiner	K/S/H	negative
	Kalamazoo River, Kalamazoo County	wild sand shiner	K/S/H	negative
	Aquaculture Facility 6, Alcona County	aquaculture-raised brook trout	K/S/H	negative
8/16/10	Wolf Lake State Fish Hatchery	hatchery fingerling muskellunge	K/S	negative
8/18/10	Marquette State Fish Hatchery	hatchery fingerling brook trout	K/S	negative
	Kalamazoo River, Calhoun County	wild golden redhorse sucker	K/S/H	negative
	Kalamazoo River, Calhoun County	wild common shiner	K/S/H	negative
	Kalamazoo River, Calhoun County	wild rockbass	K/S/H	negative
8/20/10	Platte River State Fish Hatchery	hatchery propagated steelhead	K/S/H	negative
8/24/10	Thompson State Fish Hatchery	hatchery brown trout	K/S/H	negative
	Little Manistee River, Johnsons Bridge, Lake County	wild rainbow trout	K/S/H	negative
	Lake Superior, Keweenaw Bay	wild lake trout	K/S	negative
	Lake Superior, Ontonagon	wild lake trout	K/S	negative
	Lake Superior, Ontonagon	wild lake whitefish	K/S	negative
8/25/10	Marquette State Fish Hatchery	hatchery spawning brook trout	K/S/H	negative
	Marquette State Fish Hatchery	hatchery spawning lake trout	K/S/H	negative
	Jordan River, Antrim County	feral coho salmon	K/S/H	negative
	Jordan River, Antrim County	wild mottled sculpin	K/S/H	negative

Supplementary Table S7. *Cont.*

Date	Location	Species & Rearing Condition	Tissue Examined	VHSV Results
8/31/10	Lake Superior, Black River	wild longnose sucker	K/S/H	negative
	Lake Superior, Black River	wild lake trout	K/S	negative
	Lake Superior, Black River	wild lake whitefish	K/S/H	negative
9/2/10	Aquaculture Facility 33, Chippewa County	aquaculture-raised walleye	K/S/H	negative
9/9/10	Aquaculture Facility 32, Gogebic County	aquaculture-raised brook trout	K/S/H	negative
9/16/10	I-75 Rearing Pond, Roscommon County	wild walleye	K/S/H	negative
9/22/10	Lake Huron, Saginaw Bay	wild spottail shiner	K/S/H	negative
	Lake Huron, Saginaw Bay	wild round goby	K/S/H	negative
	Lake Huron, Saginaw Bay	wild walleye	K/S/H	negative
	Lake Huron, Saginaw Bay	wild gizzard shad	K/S/H	negative
	Lake Huron, Saginaw Bay	wild trout perch	K/S/H	negative
	Lake Huron, Saginaw Bay	wild yellow perch	K/S/H	negative
	Lake Huron, Saginaw Bay	wild white perch	K/S/H	negative
	Lake Huron, Saginaw Bay	wild freshwater drum	K/S/H	negative
10/7/10	Swan River Weir	feral spawning chinook salmon	K/S, OF/milt	negative
10/10/10	Lake Superior, Marquette	wild lake whitefish	K/S	negative
	Lake Superior, Marquette	wild longnose sucker	K/S	negative
10/12/10	Little Manistee River Weir	feral spawning chinook salmon	K/S, OF/milt	negative
10/15/10	Aquaculture Facility 29, Iosco County	aquaculture-raised rainbow trout	K/S/H	negative
10/18/10	Platte River Weir	feral spawning coho salmon	K/S, OF/milt	negative
10/21/10	Marquette State Fish Hatchery	hatchery lake trout	K/S/H	negative
10/22/10	Wolf Lake State Fish Hatchery	hatchery propagated steelhead	K/S/H	negative
11/3/10	Oden State Fish Hatchery	hatchery spawning brown trout	K/S/H	negative
	Oden State Fish Hatchery	hatchery spawning rainbow trout	K/S/H	negative
	Oden State Fish Hatchery	hatchery brown trout	K/S/H	negative
11/9/10	Bait Collection Facility 10, Lapeer County	wild emerald shiner	K/S/H	negative
11/10/10	Aquaculture Facility 18, Jackson County	aquaculture-raised rainbow trout	K/S/H	negative
	Oden State Fish Hatchery	hatchery spawning brown trout	OF	negative

Supplementary Table S7. *Cont.*

Date	Location	Species & Rearing Condition	Tissue Examined	VHSV Results
11/11/10	Lake Superior State University	feral spawning Atlantic salmon	K/S, OF/milt	negative
	Bait Collection Facility 19, Arenac County	wild emerald shiner	K/S/H	negative
11/16/10	St. Mary's River, Chippewa County	wild lake herring	K/S	negative
11/23/10	Bait Collection Facility 14, Alcona County	wild emerald shiner	K/S/H	negative
	Bait Collection Facility 14, Alcona County	wild spottail shiner	K/S/H	negative
11/30/10	Bait Collection Facility 21, St. Clair County	wild emerald shiner	K/S/H	negative
12/2/10	Shupac Lake, Crawford County	wild bluegill	K/S/H	negative
	Shupac Lake, Crawford County	wild largemouth bass	K/S/H	negative
	Shupac Lake, Crawford County	wild green sunfish	K/S/H	negative
	Shupac Lake, Crawford County	wild yellow perch	K/S/H	negative
	Shupac Lake, Crawford County	wild rockbass	K/S/H	negative
	Shupac Lake, Crawford County	wild smallmouth bass	K/S/H	negative
12/7/10	Oden State Fish Hatchery	hatchery spawning brown trout	OF	negative
12/8/10	Wolf Lake State Fish Hatchery	hatchery fingerling muskellunge	K/S	negative
	Bait Collection Facility 10, Lapeer County	wild emerald shiner	K/S/H	negative
12/21/10	Bait Collection Facility 19, Arenac County	wild emerald shiner	K/S/H	negative
	Bait Collection Facility 19, Arenac County	wild spottail shiner	K/S/H	negative
